# Novel empress SARANI optimization algorithm for active power loss reduction and voltage stability enhancement

**DOI:** 10.1016/j.heliyon.2024.e38984

**Published:** 2024-10-09

**Authors:** Lenin Kanagasabai

**Affiliations:** Prasad V.Potluri Siddhartha Institute of Technology, Kanuru, Vijayawada, Andhra Pradesh, 520007, India

**Keywords:** Pomarine jaeger, Tiger hunting, DesertReynard, Vixen, Lonchodidae, Caracal, Dryocopus martius, Barasingha, Amur leopard, Empress SARANI

## Abstract

This paper proposes Pomarine jaeger Optimization (PJO) algorithm, Tiger hunting Optimization (THO) Algorithm, Desert Reynard and Vixen Inspired Optimization (DRVIO) Algorithm, Lonchodidae optimization (LO) algorithm, Caracal optimization (CO) algorithm, Barasingha optimization (BO) algorithm, Amur leopard optimization (AO) algorithm and Empress SARANI Optimization Algorithm to solve the active power loss reduction problem. Regular actions of Pomarine jaeger have been emulated to model the PJO procedure. In THO algorithm, how the Tiger moves to capture the prey is imitated and formulated. In DRVIO algorithm, Desert Reynard and Vixen burrowing capability and spurt tactic from desolate slayers are imitated to formulate the algorithm. LO algorithm emulates the physiognomies of convergent progression, track reliance, populace development and rivalry in the growth of the Lonchodidae populace in environment. In CO approach, Caracal assaults the designated quarry and then quests the quarry in a dashing procedure. BO algorithm stimulated by the Barasingha existence capability in the slayer subjugated atmosphere. AO algorithm imitates the Amur leopard behaviour. Movement paths, stalking, breeding and death are the some phases in the Amur leopard life cycle. Empress SARANI Optimization Algorithm is designed by integrating Parastylotermes Empress inspired optimization (PEIO) algorithm, Dryocopus martius optimization (DMO) algorithm, Ostrya Carpinifolia Search Optimization (OCSO) Algorithm, Hermitage Activities Inspired optimization (HAIO) algorithm with SARANI algorithm. Validity of Empress SARANI Optimization Algorithm is verified in 24 benchmark functions, IEEE and Practical systems. Real power loss (MW) obtained by projected algorithms for **IEEE 57 bus system** is PJO-21.99, THO-22.79, DRVIO-21.79, LO-23.16, CO-23.92, BO-22.81, AO- 24.89 and **Empress SARANI- 20.91.** For **IEEE 300 bus system** is PJO-395.153, THO-397.398, DRVIO-394.208, LO-398.192, CO-398.397, BO-395.209, AO-399.884 and **Empress SARANI-389.217**. For **IEEE 354 bus system** is PJO-336.108, THO-339.563, DRVIO-339.099, LO-340.164, CO-340.592, BO 338.906, AO-342.184 and **Empress SARANI-334.098**. For **practical system - WDN 220 KV** is PJO-29. 008, THO-30. 929, DRVIO-28. 519, LO-31.265, CO-31. 893, BO-29.872, AO-32.899, **Empress SARANI-26.101**.

## 1 Introduction

Active power loss diminishing problem is planned as one of the remarkable conditions for compassionate and financial action of structure [[1], [2], [3], [4], [5], [6], [7]]. It is admirable by suitable unification of the network apparatus castoff to deal with up the power flow with the objective of lessening the losses and progression the power outline of the assemblage [8]. Abundantpreceding studies untied the power problem as the optimization objective tasks calculated noticeably [[9], [10], [11], [12], [13]]. Many new approaches are sequentially applied in recent years and Table 1 show the methods utilized for solving various problems around the world.

**Table 1:** Methods utilized for solving the problem.

Method	Author	Year
Chaotic Hybrid Intelligence Strategy [14]	Sheila Mahapatra et al.	2022
Hybrid cuckoo search and ant lion optimizer [15]	Sheila Mahapatra et al.	2022
Hybrid moth–flame algorithm with particle swarm optimization [16]	Muhammad Suhail Shaikh et al.	2022
Modified whale optimization algorithm [17]	Muhammad Suhail Shaikh et al.	2022
QOHS algorithm [18]	Shiva et al.	2022
Hybrid Soft Computing Techniques [19]	Sheila Mahapatra et al.	2022
Hybrid GWO-PSO [20]	Badi et al.	2022
Ameliorated Harris hawk optimizer [21]	Sheila Mahapatra et al.	2021
Hybrid Harris Hawk Particle Swarm Optimizer [22]	Swetha Shekarappa et al.	2021
Quasi-Oppositional Salp Swarm Algorithm [23]	Raj et al.	2021
Canadian Yukon Cougar Optimization Algorithm [24]	L. Kanagasabai	2022
Quantum based Northem Rockhopper Penguin Optimization Algorithm [9]	L. Kanagasabai	2022
Caulerpa Lentillifera Algorithm	K. Lenin	2022
Buoyancy based optimization algorithm [10]	K. Lenin	2022
Gradient based optimization algorithm [11]	K. Lenin	2022
Mathematics based calculation and stemonitis inspired optimization algorithms [12]	K. Lenin	2022
Slime Mould Algorithm [25]	Soleimanian Gharehchopogh, Farhad et al.	2023
Improved Harris Hawks Optimization Algorithm [26]	Soleimanian Gharehchopogh, Farhad et al.	2023
Improved Farmland Fertility Algorithm [27]	Soleimanian Gharehchopogh, Farhad et al.	2022
Quantum-inspired metaheuristic algorithms [28]	Soleimanian Gharehchopogh, Farhad et al.	2022
An improved cuckoo search optimization algorithm [29]	Soleimanian Gharehchopogh, Farhad et al.	2022
Sparrow Search Algorithm [30]	Soleimanian Gharehchopogh, Farhad et al.	2023
Farmland Fertility Algorithm [31]	Soleimanian Gharehchopogh, Farhad et al.	2022
Wrapper-based feature selection [32]	Soleimanian Gharehchopogh, Farhad et al.	2020
Multi-agent system [33]	Soleimanian Gharehchopogh, Farhad et al.	2021
Design algorithm [34]	Vodchits, Angelina et al.	2020
An improved chimp optimization algorithm [35]	Qian et al.	2024
Evolving Marine Predators Algorithm [36]	Shen B et al.	2023
Greedy opposition-based learning for chimp optimization algorithm [37]	Khishe, M	2023
Fuzzy whale optimization algorithm [38]	Saffari A et al.	2022
Evolving chimp optimization algorithm [39]	Bo Q et al.	2023
Multi-Objective Chimp Optimizer [40]	Khishe, M et al.	2022
Robust Grey Wolf Optimizer [41]	Wang B et al.	2022
Robust universal learning chimp optimization [42]	Liu L et al.	2022
Niching Chimp Optimization [43]	Gong S et al.	2022
Dynamic Levy Flight Chimp Optimization [44]	Kaidi W et al.	2021
A Weighted Chimp Optimization Algorithm [45]	Khishe, M et al.	2021
A New Binary Meta-heuristic algorithm [46]	Wang J et al.	2021

This paper proposes Pomarine jaeger Optimization (PJO) algorithm, Tiger hunting Optimization (THO) Algorithm, Desert Reynard and Vixen Inspired Optimization (DRVIO) Algorithm, Lonchodidae optimization (LO) algorithm, Caracal optimization (CO) algorithm, Barasingha optimization (BO) algorithm, Amur leopard optimization (AO) algorithm and Empress SARANI Optimization Algorithm to solve the problem.

In Pomarine jaeger Optimization (PJO) algorithm, dualistic actions of Pomarine jaeger are principally employed to augment the exploration and exploitation Chaotic sequences are integrated in the PJO algorithm and it will augment the Exploration and Exploitation process. Quantum features are reliable pounded of median and it has been integrated with Pomarine jaeger Optimization algorithm Opposition based learning is also integrated in the Pomarine jaeger Optimization algorithm and it applies the Laplace distribution to augment the exploration dexterity.

In Tiger hunting Optimization (THO) Algorithm, the populace is organized and engaged in the exploration region by means of a sequence of guidelines and tactics enthused by the projected algorithm. In iterations, the location of every associate of the populace is rationalized rendering to the guidelines of the projected algorithm, and the novel location is appraised with the objective function to progress with iterations.

In Desert Reynard and Vixen Inspired Optimization (DRVIO) Algorithm, in the segment of Exploitation (burrowing for quarry) the Desert Reynard and Vixen stalks at night-time and single-handedly. It usages the supremacy of hearing long distance, its big ears to perceive quarry underneath the Desert soil and, subsequently it, digs by using its feet to stalk its quarry. This Desert Reynard and Vixen deeds is a local search, and pretending this performance upsurges the DRVIO exploitation influence in accomplishing a solution nearer to the global optimum.

Lonchodidae optimization (LO) algorithm emulates the physiognomies of convergent progression, track reliance, populace development and rivalry in the growth of the Lonchodidae populace in environment. The Lonchodidae populace inclines to be the nearby leading populace in the development procedure, and encouraging progression drift is additional prospective to be congenital by the ensuing generation. This paper pools population development and rivalry replicas to attain the procedure. The progression of the Lonchodidae population is diligently connected to the imitate alteration to the atmosphere. The development tendency of the Lonchodidae population is to alter with ecological variations and assimilate the aforementioned into the nearby surroundings. Consequently, the drive of the solution in the result region can be observed as the evolutionary drift of the Lonchodidae populace, and the alteration in fitness rate earlier and later the solution drive can be viewed as the alteration of the atmosphere. The Lonchodidae populace is pretentious by the ecological alteration and will yield the succeeding evolutionary drift. The subsequent evolutionary drift is pretentious by numerous physiognomies. By reviewing the evolution procedure ofLonchodidae, convergent progression, evolutionary track necessity, populace alteration, populace development and rivalry replicas are defined.

CO algorithm replicates the regular performance of Caracal in environment. The important stimulation of Caracal optimization algorithm is the stalking scheme; Caracal assaults the designated quarry and then quests the quarry in a dashing procedure. Caracal is an expert hunter that quests its quarry in three phases. Principally, by means of its robust intelligence of earshot, it recognizes the location of the quarry and detects and observes the quarry for more than 16 min deprived of any sort of movement. In the second phase Caracal passages in the direction of the quarry, hurdles up in the air and bouts the quarry. In last phase dashing procedure by running and hurdling to clasp the absconding quarry, the Caracalmurders it and consumes it. These strategies are imitated to formulate the CO algorithm. In the exploration phase, Caracalutilizes its robust intelligence of earshot to recognize the position of its quarry and assault it. Initially the locations of Caracals are rationalized grounded on the imitation of these twofold schemes. This modernization grounds large alterations in the location of Caracals and tips to a comprehensive look over in examination region. Location of the population's preeminent associate is measured as the quarry location. In the exploitation phase, subsequent to confronting the quarry, the Caracal attempts to halt the quarry by hurdling in a pursuit method, then Caracalmurder the quarry and consume it. This aspect has been imitated to update the location of the Caracal. The simulation of the chase process causes small changes in the positions of the Caracals in the search space.

BO algorithm stimulated by the Barasingha existence capability in the slayer subjugated atmosphere. Each day, the Barasingha discerns that if it does not outpace and outwit its slayers, it turn out to be victim of the day for the slayer. Even with their being downcast in the nutriment cable, they are not categorized as scarce; this indicates that Barasingha is performing approximately correct. Mainly Barasingha will spurt from slayers. In the projected BO approach the Exploration segment of the procedure, the Barasingha is foraging peaceably in the absenteeism of the slayer or even though the slayer is trailing it. BO drives into the exploitation segment as soon as a slayer is mottled, it entails of the Barasingha outpacing and outwitting the slayer to an anchorage. Both the slayer and victim as considered as search agents. Since by the period a slayer is mottled trailing the Barasingha, then both run in the similar track in the direction of the harmless anchorage, and while the Barasingha spurt out, the slayer will have also discovered the exploration region. At the end iterations, the Leading Barasingha will be rationalized if the enhanced Barasingha substitutes the Leading Barasingha.

Proposed AO algorithm imitates the Amur leopard behaviour. Movement paths, stalking, breeding and death are the some phases in the Amur leopard life cycle. These phases are mathematically designed in the projected algorithm. Each Amur leopard is an associate of the procedure population. An assured quantity of Amur leopard acts as examination agents are associates of the AO algorithm.In the subsequent segment of modernizing the associates of the population, the activities of Amur leopard during stalking and confronting the prey are utilized. Fresh location of the Amur leopard subsequentto the bout on the prey is replicated. An active modernization is used in which the fresh location is adequate to the procedure associate if the rate of the objective function in the fresh location is extra suitable than the preceding location.In the death segment, even though reproduction upsurges the populace ofAmur leopard, the quantity of Amur leopard remnants constant throughout the reproduction of the procedure owing to death. In the projected AO, it is presumed that in every imitation after reproduction, Amur leopard face death accurately as the quantity of offspring's. The condition of Amur leopard death in the AO procedure is the rate of the objective functional value. Consequently, Amur leopard which possess weedier objective functional value are more inclined to demise.

SARANI algorithm is designed by integrating Sine -Cosine Optimization Algorithm (SCA), Red Dhole Optimizer Algorithm (ROA), North Sumatra Island Pongo abelii optimization Algorithm (NIA), Dryocopus martius optimization (DMO) algorithm.

Empress SARANI Optimization Algorithm is designed by integrating Parastylotermes Empress inspired optimization (PEIO) algorithm, Dryocopus martius optimization (DMO) algorithm, Ostrya Carpinifolia Search Optimization (OCSO) Algorithm, Hermitage Activities Inspired optimization (HAIO) algorithm with SARANI algorithm.

Grounded on the functions of Sine -Cosine an algorithm is sculpted and it titled as Sine Cosine Optimization Algorithm (SCA). It produces principal whimsical agent solutions and it vacillates on the external or internal in the way of the superlative solution by expending mathematical model which grounded on the functions of sine and cosine. Red Dhole Optimizer Algorithm (ROA) derived from normal activities of Red Dhole which is deceitful and highbrow. When a garden-fresh nourishment spring is found, then Red Dhole harvests an exemplary tittering sound to interrelate about the nourishment spring discoveries. Pairing between Red Dhole encompass an expanse of minute intercourse within trifling intermezzos. North Sumatra Island Pongo abelii optimization algorithm (NIA) is sculpted grounded on the distinct acumen and sensual inducement in group. Preliminary solution is anticipated to be heightened and acquainted of the scene of the objective by the accoster, hitch, trailer and teamster. In the successive segment four supplementary things are still accomplished optimum solutions are hoarded and the additional North Sumatra Island Pongo abelii are obligatory to revolutionize their specific location to the dominant places of Pongo abelii. SARANI uniting functions of sine-cosine, antagonizing feint of Red Dhole optimizer Algorithm with North Sumatra Island Pongo abelii optimization algorithm will act as initial step of the method.Primarily male Dryocopus martius will be in gargantuan number and in the length of the initial phase of association the sum of male Dryocopus martius reduces due to association. As soon as iteration upsurges exactly the population reduces and through this exploration and exploitation is balanced. In the combined SARANI approach NIA steps move in the way of spotting the optimum solution and reformed stages are smeared to accomplish the optimal in the exploration zone. This passageway has simplified promptly to complete the leading global optimal solution.

Empress SARANI Optimization Algorithm is modelled by amalgamating Parastylotermes Empress inspired optimization (PEIO) algorithm, Dryocopus martius optimization (DMO) algorithm, Ostrya Carpinifolia Search Optimization (OCSO) Algorithm, Hermitage Activities Inspired optimization (HAIO) algorithm with SARANI algorithm. PEIO algorithm is stimulated by the separation of workers in Parastylotermes populations, which distinguishes entities by individuality and modernizes their positions in dissimilar methods. PEIO algorithm owns the individualities as: Empress, hovering employee, scavenging employee, aiding employee, and combatant. The finest solution signifies the Empress, and additional candidate elucidations are distinguished by the outstanding individualities in iterations. In DMO algorithm, preliminary segment female Dryocopus martius will get charmed rendering to the intensity of Choral sound, conversely at finishing segment it will be charmed in the course of most superb male Dryocopus martius. Female Dryocopus martius first pay attention to solo male Dryocopus martius Choral sound and at the finishing segment, the male associate with the female Dryocopus martius and most superb high intensity of Choral sound. Initially male Dryocopus martius will be in gargantuan number and in the length of the initial phase of association the sum of male Dryocopus martius reduces due to association. As soon as iteration upsurges exactly the population reduces and through this exploration and exploitation is balanced. In OCSO growing segment, Ostrya Carpinifolia competes for growing and progresses their fitness rate. In the seed sprinkling segment exploration is attained through sprinkling of Ostrya Carpinifolia seeds around the region. In the root dissemination segment, exploitation is attained through the dissemination of Ostrya Carpinifolia roots around the region. In HAIO Followers, generative followers and protecting follower's actions are imitated to balance the exploration and exploitation in the procedure. Preliminary population steadily registers the data obtained at iterations and communicates it to followers and protecting followers in subsequent iteration. This process is recurrent up until the comprehensive optimum is established.

### 1.1 New contributions in the research paper

In this paper Pomarine jaeger Optimization (PJO) algorithm, Tiger hunting Optimization (THO) Algorithm, Desert Reynard and Vixen Inspired Optimization (DRVIO) Algorithm, Lonchodidae optimization (LO) algorithm, Caracal optimization (CO) algorithm, Barasingha optimization (BO) algorithm, Amur leopard optimization (AO) algorithm and Empress SARANI Optimization Algorithm. Sine -Cosine Optimization Algorithm (SCA), Red Dhole Optimizer Algorithm (ROA), North Sumatra Island Pongo abelii optimization Algorithm (NIA), Dryocopus martius optimization (DMO) algorithm are integrated and is entitled as SARANI algorithm are newly designed and formulated to solve the problem. Empress SARANI Optimization Algorithm is modelled by amalgamating Parastylotermes Empress inspired optimization (PEIO) algorithm, Dryocopus martius optimization (DMO) algorithm, Ostrya Carpinifolia Search Optimization (OCSO) Algorithm, Hermitage Activities Inspired optimization (HAIO) algorithm with SARANI algorithm.Hybridization between the algorithms has been accomplished to enhance the quality of the solutions.

### 1.2 Highlights of the paper

• In Pomarine jaeger Optimization (PJO) algorithm, dualistic actions of Pomarine jaeger are principally employed to augment the exploration and exploitation, then Chaotic sequences, Quantum features and Opposition based learning is integrated in the Pomarine jaeger Optimization algorithm.
• In Tiger hunting Optimization (THO) Algorithm, the populace is organized and engaged in the exploration region by means of a sequence of guidelines and tactics enthused by the projected algorithm.
• In Desert Reynard and Vixen Inspired Optimization (DRVIO) Algorithm, in the segment of Exploitation (burrowing for quarry) the Desert Reynard and Vixen stalks at night-time and single-handedly.
• Lonchodidae optimization (LO) algorithm emulates the physiognomies of convergent progression, track reliance, populace development and rivalry in the growth of the Lonchodidae populace in environment.
• CO algorithm replicates the regular performance of Caracal in environment. The important stimulation of Caracal optimization algorithm is the stalking scheme; Caracal assaults the designated quarry and then quests the quarry in a dashing procedure.
• BO algorithm stimulated by the Barasingha existence capability in the slayer subjugated atmosphere. Each day, the Barasingha discerns that if it does not outpace and outwit its slayers, it turn out to be victim of the day for the slayer.
• AO algorithm imitates the Amur leopard behaviour. Movement paths, stalking, breeding and death are the some phases in the Amur leopard life cycle. These phases are mathematically designed in the projected algorithm.
• Sine -Cosine Optimization Algorithm (SCA), Red Dhole Optimizer Algorithm (ROA), North Sumatra Island Pongo abelii optimization Algorithm (NIA), Dryocopus martius optimization (DMO) algorithm are integrated and entitled as SARANI algorithm.
• Empress SARANI Optimization Algorithm is designed by integrating Parastylotermes Empress inspired optimization (PEIO) algorithm, Dryocopus martius optimization (DMO) algorithm, Ostrya Carpinifolia Search Optimization (OCSO) Algorithm, Hermitage Activities Inspired optimization (HAIO) algorithm with SARANI algorithm.

Empress SARANI Optimization Algorithm performed well in solving the real power loss reduction and voltage stability enhancement.

### 1.3 Limitations of the projected algorithms

Empress SARANI Optimization Algorithm is validated in Benchmarking functions, IEEE test systems and practical 220 KV systems. Yet the algorithms have to be tuned further to apply for the real time systems of other area of engineering.

#### 1.3.1 Structure of the paper

Sections has been arranged as follows.

a. Pomarine jaeger Optimization (PJO) algorithm
b. Tiger hunting Optimization (THO) Algorithm
c. Desert Reynard and Vixen Inspired Optimization (DRVIO) Algorithm
d. Lonchodidae optimization (LO) algorithm
e. Caracal optimization (CO) algorithm
f. Barasingha optimization (BO) algorithm
g. Amur leopard optimization (AO) algorithm
h. Empress SARANI Optimization Algorithm
i. Simulation study

## 2 Problem formulation

Objective function of the problem is systematically demarcated in widespread mode by,

(1) $$Minimization\bar{F}\left(\bar{y},\bar{z}\right)$$

Subject to

(2) $$a\left(\bar{y},\bar{z}\right)=0$$

(3) $$b\left(\bar{y},\bar{z}\right)=0$$

$$Minimization\bar{F}\;define\;the\;objetcive\;function$$

(4) $$y=\left[{VG}_{1},..,{VG}_{Ng};{QC}_{1},..,{QC}_{Nc};T_{1},..,T_{N_{T}}\right]$$

(5) $$z=\left[{PG}_{slack};{VL}_{1},..,{VL}_{N_{Load}};{QG}_{1},..,{QG}_{Ng};{SL}_{1},..,{SL}_{N_{T}}\right]$$

Fitness function $\left({Fit}_{t1}\right)$ is demarcated for lessening the power loss,

(6) $${Fit}_{t1}=P_{\mathrm{Min}}=\mathrm{Min}\left[\sum_{m}^{NTL}G_{m}\left[V_{i}^{2}+V_{j}^{2}-2*V_{i}V_{j}\;\cos\;{Ø}_{ij}\right]\right]$$

Minimization of Power aberration's fitness function $\left({Fit}_{t2}\right)$ is quantified as,

(7) $${Fit}_{t2}=\mathrm{Min}\left[\sum_{i=1}^{N_{LB}}{\left|V_{Lk}-V_{Lk}^{desired}\right|}^{2}+\sum_{i=1}^{Ng}{\left|Q_{GK}-Q_{KG}^{\mathrm{Lim}}\right|}^{2}\right]$$

Power permanence fitness function $\left({Fit}_{t3}\right)$ is quantified as,

(8) $${Fit}_{3}=\mathrm{Min}\;L_{\mathrm{Max}}$$

(9) $$L_{\mathrm{Max}}=\mathrm{Max}\left[L_{j}\right];j=1;N_{LB}$$

(10) $$\left\{ \begin{array}{c} L_{j}=1-\sum_{i=1}^{NPV}F_{ji}\frac{V_{i}}{V_{j}}\\ F_{ji}=-{\left[Y_{1}\right]}^{1}\left[Y_{2}\right] \end{array}\right.$$

(11) $$L_{\mathrm{Max}}=\mathrm{Max}\left[1-{\left[Y_{1}\right]}^{-1}\left[Y_{2}\right]\times \frac{V_{i}}{V_{j}}\right]$$

Parity constraints

(12) $$0={PG}_{i}-{PD}_{i}-V_{i}\sum_{j\in N_{B}}V_{j}\left[G_{ij}\;\cos\left[{Ø}_{i}-{Ø}_{j}\right]+B_{ij}\;\sin\left[{Ø}_{i}-{Ø}_{j}\right]\right]$$

(13) $$0={QG}_{i}-{QD}_{i}-V_{i}\sum_{j\in N_{B}}V_{j}\left[G_{ij}\;\sin\left[{Ø}_{i}-{Ø}_{j}\right]+B_{ij}\;\cos\left[{Ø}_{i}-{Ø}_{j}\right]\right]$$

Disparity constraints

(14) $${\;P}_{\text{gslack}}^{\min}\leq P_{\text{gslack}}\leq P_{\text{gslack}}^{\max}$$

(15) $${\;Q}_{\text{gi}}^{\min}\leq Q_{\text{gi}}\leq Q_{\text{gi}}^{\max},i\in N_{g}$$

(16) $${\;\text{VL}}_{i}^{\min}\leq {\text{VL}}_{i}\leq {\text{VL}}_{i}^{\max},i\in \text{NL}$$

(17) $${\;T}_{i}^{\min}\leq T_{i}\leq T_{i}^{\max},i\in N_{T}$$

(18) $${\;Q}_{c}^{\min}\leq Q_{c}\leq Q_{C}^{\max},i\in N_{C}$$

(19) $$\left|{SL}_{i}\right|\leq S_{L_{i}}^{\max},i\in N_{\text{TL}}$$

(20) $${\;\text{VG}}_{i}^{\min}\leq {\text{VG}}_{i}\leq {\text{VG}}_{i}^{\max},i\in N_{g}$$

(21) $$MOFF=F_{1}+x_{i}F_{2}+yF_{3}=F_{1}+\left[\sum_{i=1}^{NL}x_{v}{\left[{VL}_{i}-{VL}_{i}^{\min}\right]}^{2}+\sum_{i=1}^{NG}x_{g}{\left[{QG}_{i}-{QG}_{i}^{\min}\right]}^{2}\right]+x_{f}F_{3}$$

(22) $${VL}_{i}^{\min}=\left\{ \begin{array}{c} {VL}_{i}^{\max},{VL}_{i}>{VL}_{i}^{\max}\\ {VL}_{i}^{\min},{VL}_{i}<{VL}_{i}^{\min} \end{array}\right.$$

(23) $${QG}_{i}^{\min}=\left\{ \begin{array}{c} {QG}_{i}^{\max},{QG}_{i}>{QG}_{i}^{\max}\\ {QG}_{i}^{\min},{QG}_{i}<{QG}_{i}^{\min} \end{array}\right.$$

## 3 Pomarine jaeger optimization algorithm

Regularactions of Pomarine jaeger have been emulated to model the Pomarine jaeger Optimization procedure. Pomarine jaeger sounds loud and does spasm to attain the prey. In commonPomarine jaeger isexplorer and predator, which consumesminorfly-fish, etc. Pomarine jaeger hasthe talent to latch the prey in rapid mode. Pomarine jaeger mobile in assemblage and the primaryloci of the Pomarine jaegerare disparatehowever it will elude the smash. PredominantlyPomarine jaegerwill hoverin the direction of the supremeresolutionaptPomarine jaegerand rendering to that enduringPomarine jaegerwill appraise their primarylocations. In the proposedPomarine jaeger Optimization (PJO) algorithm dualisticactions of Pomarine jaegerare principallyemployed to augment the exploration and exploitation. By migration Pomarine jaegerwill passage to additional regions. InassemblagePomarine jaegerwill move one location to other spot. Throughout the drivePomarine jaegerwill elude smashes and a superfluous parameter is operated for the calculation of the fresh exploration mediator.

(24) $$\vec{U_{s}}=V*\vec{W_{s}}\;\left(n\right)$$

$$where\;\vec{U_{s}}\;is\;location\;oftheexplorationagent$$

Vissuperfluousparameter

$$\vec{W_{s}}\;is\;current\;location\;oftheexplorationagent$$

nispresentiteration

The examinationmediatordriveengagements in the region is demarcated as,

(25) $$V=f_{g}-\left(n*\times \left(\frac{f_{g}}{N}\right)\right)$$

$$where\;f_{g}\;is\;frequency\;\text{governor}$$

Nismax.iter

n=0,1,2,3,..,N

$$f_{g}=1$$

$$f_{g}\;govern\;the\;vlaue\;of\;V$$

Drivein the direction of the supremefortitudeaptPomarine jaegeris defined as,

(26) $$\vec{L_{s}}=Z*\left(\left(\vec{U_{sf}}\right)\cdot \left(n\right)-\left(\vec{U_{s}}\right).\left(n\right)\right)$$

$$\vec{L_{s}}\text{Signifies}\;\text{the}\;\text{specific}\;\text{location}\;\text{of}\;\text{the}\;\text{search}\;\text{mediator}\;\text{in}\;\text{the}\;\text{course}\;\text{of}\;\vec{U_{sf}}$$

$$\vec{U_{sf}}is\;\text{supreme}\;\text{fortitude}\;\text{apt}\;\text{Pomarine}\;\text{jaeger}$$

Ztobalancebetweenexplorationandexploitation

(27) $$Z=2\times V^{2}\times r$$

whererisrandomnumber[0,1]

All examination mediators appraise the location rendering to the appropriate examination mediator's spot (fittest Pomarine jaeger location), consequently all examination mediators endure in neighbouring propinquity rendering to the appropriate examination mediator.

(28) $${\vec{Distance}}_{s}=\left|\vec{U_{s}}+\vec{E_{s}}\right|$$

$$where\;{\vec{Distance}}_{s}\;indicate\;the\;space\;between\;\text{appropriate}\;\text{examination}\;\text{mediator}\;\text{and}\;\text{others}$$

As soon as attack over the prey is insisted by the Pomarine jaeger in the midair a vortexcrusade will happen and it has been designated in a, b, c planes.

(29) $$a^{\prime }=i*\cos\left(h\right)$$

(30) $$b^{\prime }=i*\sin\left(h\right)$$

(31) $$c^{\prime }=i*h$$

(32) $$a^{\prime }=i*\cos\left(h\right)$$

(33) $$h=j*e^{mh}$$

whereispecifytheradiusofthevortexcrusade

hisrandomnumber(0,2)

jandmareconstantsinthevortexcrusade

(34) $$\vec{U_{s}}\left(n\right)=\left({\vec{Distance}}_{s}\times a^{\prime }\times b^{\prime }\times c^{\prime }\right)+\left(\vec{U_{sf}}\right)\cdot \left(n\right)$$

Drive of the procedure in pursuit of nourishment with orientation to Levy incorporated in the algorithm.

(35) L(s)∼|s|−1−βwhere0<ß<2

(36) $$L\left(s,\gamma ,\mu \right)=\left\{ \begin{array}{c} \sqrt{\frac{\gamma }{2\pi }}\\ 0\;\text{if}\;s\leq 0\; \end{array}\right.\exp\left[-\frac{\gamma }{2\left(s-\mu \right)}\right]\frac{1}{{\left(s-\mu \right)}^{3/2}}\;\text{if}\;0\;<\mu <s<\infty$$

(37) $$V_{j}^{Levy}=V_{j}+V_{j}\times \left(Levy\left(\alpha \right)\right)$$

$${where\;V}_{j}^{Levy}\;specify\;the\;location\;of\;the\;examining\;agent$$

(38) $${\vec{Distance}}_{s}.\;Levy={\vec{Distance}}_{s}+\left|\vec{U_{s}}-\vec{\vec{L_{s}}}\right|\cdot \left(Levy\left(\alpha \right)\right)$$

(39) $$\vec{U_{s}}\left(n\right)=\left({\vec{Distance}}_{s}.\;Levy\times a^{\prime }\times b^{\prime }\times c^{\prime }\right)+\left(\vec{U_{sf}}\right)\cdot \left(n\right)$$

Chaotic sequences [[9], [10], [11], [12], [13]]are integrated in the Pomarine jaeger Optimization (PJO) algorithm.

(40) $$p_{t+1}=p_{t}^{2}-q_{t}^{2}+a\cdot p_{t}+b\cdot q_{t}$$

(41) $$q_{t+1}=2p_{t}q_{t}+c\cdot p_{t}+d\cdot q_{t}$$

Quantum features [47] areintegrated with Pomarine jaeger Optimization algorithm.

(42) $${\left|\Psi \right|}^{2}\cdot dx\cdot dy\cdot dz=Q\cdot dx\cdot dy\cdot dz$$

whereΨindicateprobabilitydensity

Opposition based learning [[9], [10], [11], [12], [13]] integrated in the Pomarine jaeger Optimization algorithm.

(43) $$f\left(l\right)=\left\{ \begin{array}{c} \frac{1}{2}\;\exp\left(-\left|l-c\right|/d\right),b\leq c\\ 1-\frac{1}{2}\;\exp\left(-\left|l-c\right|/d\right),b>c \end{array}\right.$$

(44) $$S^{o}=a+b-Z$$

(45) $$S_{i}\left(iteration\right)=X_{s}*\left({\min\;imum}_{i}+{maximum}_{i}-S_{e}\left(iteration\right)\right)$$

(46) $$z=\left|f\left(S_{i}^{j}\right)-f\left(S_{c}^{j}\right)\right|/f\left(S_{c}^{j}\right)+\varphi$$

$$where\;f\left(S_{i}^{j}\right),f\left(S_{c}^{j}\right)\;defines\;the\;cost\;quantity$$

φtoevadethezeroerror

(47) $$e_{i}^{t}=1/1+e^{-c}$$

(48) $$A=\left(2\times e_{i}^{t}+1\right)\times v\times \left(1-\frac{{iteration}_{i}}{\text{maximum}iteration}\right)+n$$

**Fig. 1:**
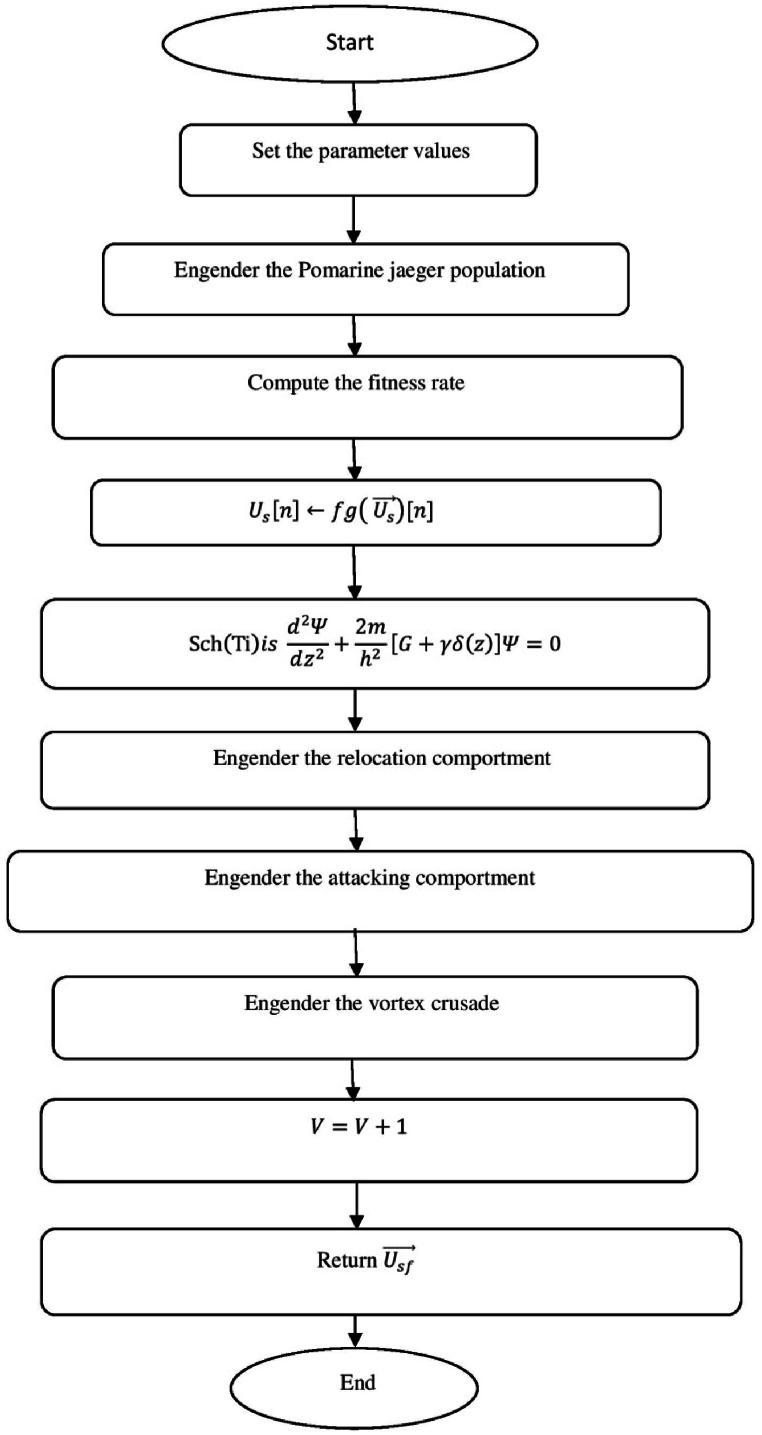
a. Schematic diagram of Pomarine jaeger Optimization (PJO) algorithm.

Fig. 1 shows the schematic diagram of Pomarine jaeger Optimization (PJO) algorithm.

a. Start
b. Set the parameter values
c. Engender the Pomarine jaegerpopulation
d. $set\;f_{g}=1$:
e. while(n<maximumiteration)do:
f. Compute the fitness rate
g. forn←1toNdo:
h. $U_{s}\left[n\right]\leftarrow fg\left(\vec{U_{s}}\right)\left[n\right]$:
i. End for
j. $\vec{U_{sf}}\leftarrow supreme\left(U\;\left[\;\right]\right)$:
k. Return $\vec{U_{sf}}$:
l. Obtainthesupreme(U[]):
m. $Supreme\leftarrow \vec{U_{sf}}\;\left[0\;\right]$:
n. forn←1toNdo:
o. $if\left(\vec{U_{sf}}\;\left[n\;\right]<\;\text{supreme}\right)then$:
p. $supreme\leftarrow \vec{U_{sf}}\;\left[n\;\right]$:
q. $\text{Sch}\left(\text{Ti}\right)is\;\frac{d^{2}\Psi }{dz^{2}}+\frac{2m}{h^{2}}\left[G+\gamma \delta \left(z\right)\right]\Psi =0$:
r. $\Psi \left(z\right)=\frac{1}{\sqrt{L}}e^{-\frac{\left|z\right|}{L}}$:
s. $Q\left(z\right)={\left|\Psi \left(z\right)\right|}^{2}=\frac{1}{\sqrt{L}}e^{-\frac{\left|z\right|}{L}}$:
t. End if
u. End for
v. Return the supreme fitness rate
w. $p_{t+1}=p_{t}^{2}-q_{t}^{2}+a\cdot p_{t}+b\cdot q_{t}$:
x. $q_{t+1}=2p_{t}q_{t}+c\cdot p_{t}+d\cdot q_{t}$:
y. $p_{t+1}^{*}=p_{t+1}-\min\left(p\right)\max\left(p\right)-minp$:
z. $f\left(l\right)=\left\{ \begin{array}{c} \frac{1}{2}\;\exp\left(-\left|l-c\right|/d\right),b\leq c\\ 1-\frac{1}{2}\;\exp\left(-\left|l-c\right|/d\right),b>c \end{array}\right.$:
aa. $S_{i}\left(iteration\right)=X_{s}*\left({minimum}_{i}+{maximum}_{i}-S_{e}\left(iteration\right)\right)$:
bb. Engender the relocationcomportment
cc. Engender the attacking comportment
dd. Engender the vortex crusade
ee. computethedistance:
ff. ${\vec{Distance}}_{s}=\left|\vec{U_{s}}+\vec{E_{s}}\right|$:
gg. $plane\leftarrow a^{\prime }\times b^{\prime }\times c^{\prime }$:
hh. $a^{\prime }=i*\cos\left(h\right)$:
ii. $b^{\prime }=i*\sin\left(h\right)$:
jj. $c^{\prime }=i*h$:
kk. $a^{\prime }=i*\cos\left(h\right)$:
ll. $h=j*e^{mh}$:
mm. $V_{j}^{Levy}=V_{j}+V_{j}\times \left(Levy\left(\alpha \right)\right)$:
nn. ${\vec{Distance}}_{s}.\;Levy={\vec{Distance}}_{s}+\left|\vec{U_{s}}-\vec{\vec{L_{s}}}\right|\cdot \left(Levy\left(\alpha \right)\right)$:
oo. Modernize the location
pp. $\vec{U_{s}}\left(n\right)=\left({\vec{Distance}}_{s}.\;Levy\times a^{\prime }\times b^{\prime }\times c^{\prime }\right)+\left(\vec{U_{sf}}\right)\cdot \left(n\right)$:
qq. V←V+1:
rr. End while
ss. Return $\vec{U_{sf}}$:
tt. End

## 4 Tiger hunting optimization algorithm

Then in this paper Tiger hunting Optimization (THO) Algorithm is applied to solve the problem. Tiger follow the prey in effective mode and it pursuits towards prey. Meanwhile the prey senses the Tiger movement and tries to flee away. The populace is organized and engaged in the exploration region by means of a sequence of guidelines and tactics enthused by the projected algorithm. In iterations, the location of every associate of the populace is rationalized rendering to the guidelines of the projected algorithm, and the novel location is appraised with the objective function to progress with iterations.

Tiger movement in order to reach the prey is imitated and formulated. Principally the primary populace is arbitrarily created,

(49) $$\left(\vec{z}\right)=\left\{{\vec{z}}_{1},{\vec{z}}_{2},{\vec{z}}_{3},..{\vec{z}}_{n}\right\}$$

Then the objective function is calculated for all associates of the population as,

(50) $$\left(\vec{U}\right)=\left\{U_{1},U_{2},U_{3},..,U_{n}\right\}$$

The location of each member in the preliminary population is engendered in the examination zone,

(51) $$z_{i}=R\left(1,d\right)*\left(\max-\min\right)+\min$$

whereR∈[0,1]

$$z_{i}\;specify\;the\;location$$

maxandmindefinethelimits

(52) $$\min=\left[\min_{1},\min_{2},\min_{3},..,\min_{d}\right]$$

(53) $$\max=\left[\max_{1},\max_{2},\max_{3},..,\max_{d}\right]$$

wheredspecifythedimensionoftheproblem

Subsequent to engendering the preliminary populace and shaping every representative's location, every solution's appropriateness is premeditated as,

$$U_{n}=f\left(\vec{z}\right)$$

Computing the appropriateness function governs the quality of the solution. Exploration and Exploitation has been balanced in the projected approach.

The Tiger will search for prey deliberately and try to fix one for further actions,

(54) $$z_{i,j}\left(t+1\right)=z_{i,j}\left(t\right)+0.50\left[\left(2GHQ_{location\left(j\right)}-z_{i,j}\left(t\right)\right)+2\left(1-G\right)H\rho_{\left(j\right)}-z_{i,j}\left(t\right)\right]$$

z(t)definetheTigerlocation

z(t+1)specifythemalenextposition

$$Q_{location}\;define\;the\;location\;of\;the\;prey$$

ρspecifythecompletelocation

Histheadaptivefactor

(55) $$Q={\vec{Random}}_{1}<G;\;Index=\left(Q==0\right)$$

(56) $$H={Random}_{2}⊗index+{\vec{Random}}_{3}⊗\left(∼index\right)$$

$$R_{1},{R_{2},R}_{3}\in \left[0,1\right]$$

(57) $$G=i-iteration\left(\frac{0.99}{maximum\;iteration}\right)$$

G→balancetheexplorationandexploitation

In order to calculate the location of the prey ρ is calculated as,

(58) $$\rho =\frac{1}{n}\sum_{i=1}^{n}{\vec{z}}_{i}$$

Then the distance is computed as follows,

(59) $${Distance}_{i}={\left(\sum_{j=1}^{d}{\left(z_{i,j}-\rho_{j}\right)}^{2}\right)}^{\frac{1}{2}}$$

(60) $$Q_{location}={\vec{z}}_{i}|i\to index\;of\;\max\left(end\right)classify\left({Distance}_{i}\right)$$

(61) $$k_{b}=r\left(G\times N\right)$$

N→numberofagents

r→round

$$k_{b}\to k\;best$$

Then the prey location is computed as,

(62) $${\vec{Q}}_{location}={\vec{z}}_{i}|i\to classified\;{Distance}_{i}\left(k_{b}\right)$$

$$initial\;phase\;of\;the\;algorithm\;\to k_{b}=N$$

$$last\;segment\;of\;the\;algorithm\;\to vlaue\;of\;k_{b}\;decreases$$

Best harmless location is the optimum global location since it will provide the prey a superior chance of endurance and sequentially the Tiger will try to identify another prey

(63) $$z_{i,j}\left(t+1\right)=T_{Location}\left(j\right)+GHcos\left(2\pi {Random}_{4}\right)*\left(T_{Location}\left(j\right)-z_{i,j}\left(t\right)\right)$$

z(t)definetheTigerlocation

z(t+1)specifytheTigernextposition

$$T_{Location}\left(j\right)\;specify\;the\;optimum\;global\;point$$

$${Random}_{4}\in \left[-1,1\right]$$

Mainly in the projected design the Tiger and the prey is chosen mathematically as follows,

(64) $$z_{i}\left(t+1\right)=\left\{ \begin{array}{c} z_{i,j}\left(t\right)+0.50\left[\left(2GHQ_{location\left(j\right)}-z_{i,j}\left(t\right)\right)+2\left(1-G\right)H\rho_{\left(j\right)}-z_{i,j}\left(t\right)\right],if{Random}_{5}<\alpha \;\\ T_{Location}\left(j\right)+GHcos\left(2\pi {Random}_{4}\right)*\left(T_{Location}\left(j\right)-z_{i,j}\left(t\right)\right)Else \end{array}\right.$$

z(t)definetheTigerlocation

z(t+1)specifytheTigernextposition

$$Q_{location}\;define\;the\;location\;of\;the\;prey$$

ρspecifythecompletelocation

Histheadaptivefactor

z(t)definetheTigerlocation

z(t+1)specifytheTigernextposition

$$T_{Location}\left(j\right)\;specify\;the\;optimum\;global\;point$$

$${Random}_{4}\in \left[-1,1\right]$$

$${Random}_{5}\in \left[0,1\right]$$

α=0.10

$$when\;the\;{Random}_{5}<\alpha \;\to serach\;agent\;is\;Tiger$$

$$when\;the\;{Random}_{5}>\alpha \;\to serach\;agent\;is\;Prey$$

**Fig. 2:**
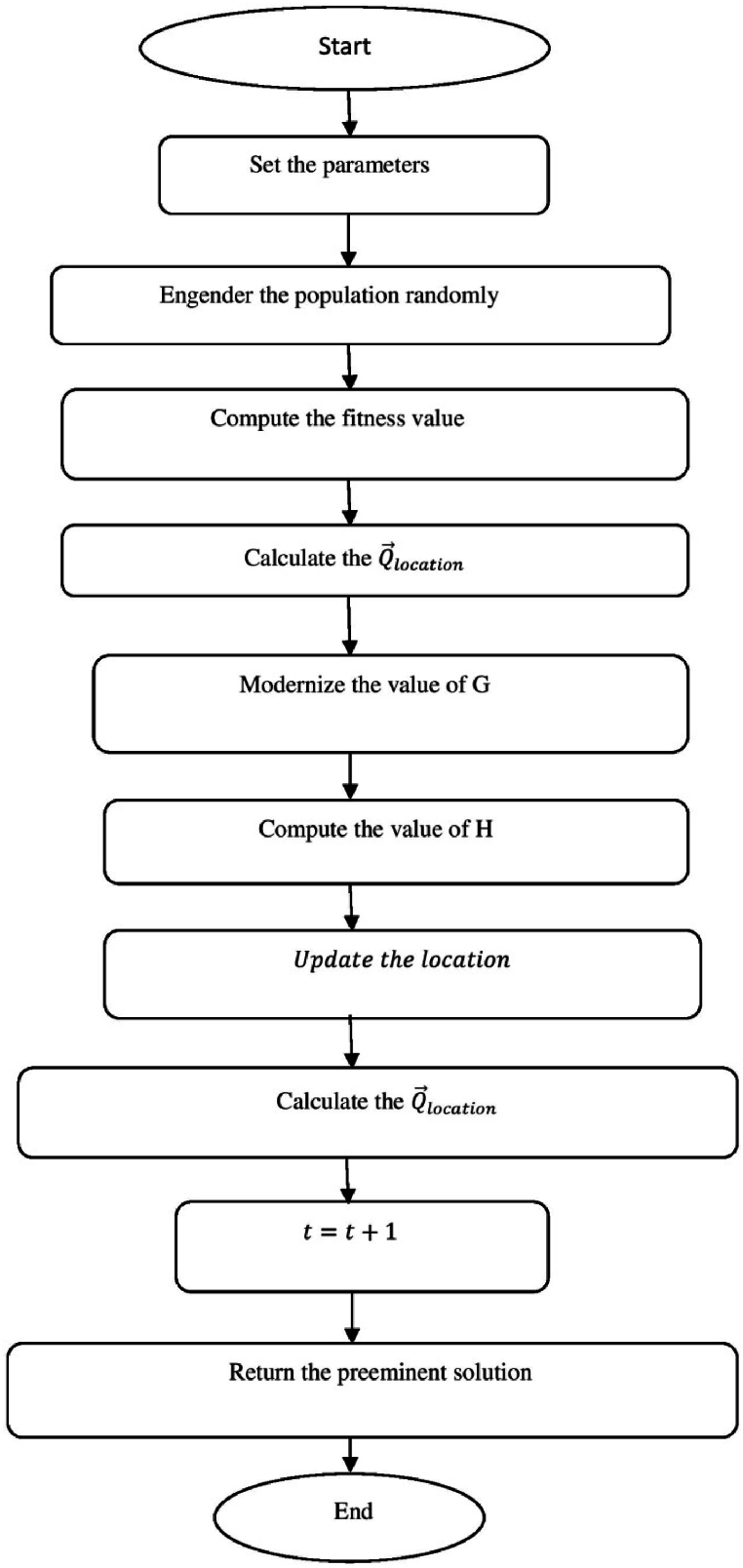
Flow chart of Tiger hunting Optimization (THO) Algorithm.

Fig. 2 shows the Flow chart of Tiger hunting Optimization (THO) Algorithm.

a. start
b. Fix the parameters
c. Engender the population randomly
d. $z_{i}=R\left(1,d\right)*\left(\max-\min\right)+\min$:
e. Compute the fitness value
f. Calculate the ${\vec{Q}}_{location}$:
g. Modernize the value of G
h. $G=i-iteration\left(\frac{0.99}{maximum\;iteration}\right)$:
i. Compute the value of H
j. $H={Random}_{2}⊗index+{\vec{Random}}_{3}⊗\left(∼index\right)$:
k. ${if\;Random}_{5}<\alpha ,then$:
l. $Compute\;{\vec{Q}}_{location}$:
m. $\rho =\frac{1}{n}\sum_{i=1}^{n}{\vec{z}}_{i}$:
n. ${\vec{Q}}_{location}={\vec{z}}_{i}|i\to classified\;{Distance}_{i}\left(k_{b}\right)$:
o. Updatethelocation:
p. $z_{i,j}\left(t+1\right)=z_{i,j}\left(t\right)+0.50\left[\left(2GHQ_{location\left(j\right)}-z_{i,j}\left(t\right)\right)+2\left(1-G\right)H\rho_{\left(j\right)}-z_{i,j}\left(t\right)\right]$:
q. Otherwise
r. Streamline the position
s. $z_{i,j}\left(t+1\right)=T_{Location}\left(j\right)+GHcos\left(2\pi {Random}_{4}\right)*\left(T_{Location}\left(j\right)-z_{i,j}\left(t\right)\right)$:
t. Compute the fitness value
u. Calculate the ${\vec{Q}}_{location}$:
v. t=t+1:
w. Output the best solution
x. End

## 5 Desert Reynard and Vixen Inspired Optimization algorithm

Then in this research work Desert Reynard and Vixen Inspired Optimization (DRVIO) Algorithm is applied to solve the problem. In the proposed algorithm Desert Reynard and Vixen burrowing capability and spurt tactic from desolate slayers are imitated to formulate the algorithm.

The Desert Reynard and Vixen possess big and it's the protuberant feature. Big ears and extraordinary hearing distance supremacy empower the Desert Reynard and Vixen to receive the sound of subversive quarry. It is a dominant miner that digs to clip these quarry in subversive. The Desert Reynard and Vixen possess tactic for hostility with slayer bouts that, by unexpectedly altering drive ways, deceive the slayer and spurt from it.The Desert Reynard and Vixen habitually stalks in restricted zones, and consequently the stalking procedure in DRVIO is replicated as a local exploration and shields only a trivial zone of the exploration region. Then the performance of Desert Reynard and Vixen while absconding from slayers is replicated to deliver a global exploration.

In DRVIO algorithm, Desert Reynard and Vixen make up exploration associates. Each Desert Reynard and Vixen signifies a contender solution then the location in the exploration region regulates the standards of the decision factors. Desert Reynard and Vixen are arbitrarily initialized in the exploration region as follows,

(65) $$Z_{i}:z_{i,j}={min}_{j}+Random\left(\max_{j}-\min_{j}\right)$$

i=1,2,3,..,N:whereN→sumofDesertReynardandVixen

j=1,2,3,..,M;whereMisdecisionfactors

$$Z_{i}\to ith\;Desert\;Reynard\;and\;Vixen$$

Random∈[0,1]

$$\max_{j}\;and\;\min_{j}define\;the\;limits$$

Then the Desert Reynard and Vixenpopulation matrix is defined as follows,

(66) $$Z={\left[\begin{array}{c} Z_{1}\\ \vdots \\ Z_{i}\\ \vdots \\ Z_{N} \end{array}\right]}_{N\times M}=\left[\begin{array}{ccc} z_{1,1} & \cdots & z_{1,M}\\ \vdots & \ddots & \vdots \\ z_{N,1} & \cdots & z_{N,M} \end{array}\right]$$

$$Z_{i}=\left(z_{i1},z_{i2},z_{i3},..,z_{iM}\right)$$

Then the vector rate is defined as,

(67) $$V={\left[\begin{array}{c} V_{1}\\ \vdots \\ V_{i}\\ \vdots \\ V_{N} \end{array}\right]}_{N\times 1}={\left[\begin{array}{c} V\left(Z_{1}\right)\\ \vdots \\ V\left(Z_{i}\right)\\ \vdots \\ V\left(Z_{N}\right) \end{array}\right]}_{N\times 1}$$

V→vectorrateoftheobjectivefunction

$$V_{i}\to ith\;Desert\;Reynard\;and\;Vixen\;objective\;functional\;value$$

Double regular deeds of Desert Reynard and Vixen are utilized to modernize the location of DRVIO associates in the exploration region. These deeds are (i) burrowing to consume quarry underneath the Desert soil (ii) fleeing from slayers.

In the segment of Exploitation (burrowing for quarry) the Desert Reynard and Vixen stalks at night-time and single-handedly. It usages the supremacy of hearing long distance, its big ears to perceive quarry underneath the Desert soil and, subsequently it, digs by using its feet to stalk its quarry. This Desert Reynard and Vixen deeds is a local search, and pretending this performance upsurges the DRVIO exploitation influence in accomplishing a solution nearer to the global optimum. To design the performance of a Desert Reynard and Vixen in the course of burrowing is done by considering vicinity round its tangible locus. The Desert Reynard and Vixen with a local examination in this zone can converge to an enhanced solution.

This segment of modernizing DRVIO associates is scientifically defined as,

(68) $$z_{i,j}^{segment1}=z_{i,j}+\left(2*Random-1\right)*{Radius}_{i,j}$$

(69) $${Radius}_{i,j}=\delta \cdot \left(1-\frac{t}{T}\right)\cdot z_{i,j}$$

(70) $$Z_{i}=\left\{ \begin{array}{c} Z_{i}^{segment1},V_{i}^{segment1}<V_{i}\\ Z_{i},Else \end{array}\right.$$

$$Z_{i}^{segment1}\;define\;the\;new\;location\;of\;Desert\;Reynard\;and\;Vixen\;in\;first\;segment$$

$$V_{i}^{segment1}\to obj.functional\;value$$

t,Tarecurrentandmaximumiterations

δ=0.2

In the segment of Exploration (flee tactics form slayers) the Desert Reynard and Vixen are exposed to bouts by desolate slayers. Desert Reynard and Vixen spurts from slayers through high speed movement and rapid variation in course of drive. In the projected scientific design, this flee tactic of the Desert Reynard and Vixen is the root of comprehensive glance over of the exploration region. Replication of this flee tactic enriches the exploration influence in the projected DRVIO algorithm and it will evade the local optima. Therefore, the arbitrary location of every candidate solution in the exploration region is considered in the design of the deeds of the Desert Reynard and Vixen for the period of its spurt. The subsequent segment of the DRVIO population modernization is scientifically replicated as follows,

(71) $$Z_{i}^{Random}:z_{i,j}^{Random}=z_{k,j}$$

k=1,2,3,..,N

(72) $$Z_{i,j}^{Segment2}=\left\{ \begin{array}{c} z_{i,j}+random\cdot \left(z_{i,j}^{Random}-Q\cdot z_{i,j}\right),V_{i}^{random}<V_{i}\\ z_{i,j}+random\cdot \left(z_{i,j}-z_{i,j}^{Random}\right),Else \end{array}\right.$$

(73) $$Z_{i}=\left\{ \begin{array}{c} Z_{i}^{segment2},V_{i}^{segment2}<V_{i}\\ Z_{i},Else \end{array}\right.$$

$$Z_{i}^{Random}\;define\;the\;aim\;location\;intended\;by\;Desert\;Reynard\;and\;Vixen\;for\;fleeing$$

$$Z_{i}^{segment2}\;define\;the\;new\;location\;of\;Desert\;Reynard\;and\;Vixen\;in\;second\;segment$$

$$V_{i}^{segment2}\to obj.functional\;value$$

Q∈{1,2}

**Fig. 3:**
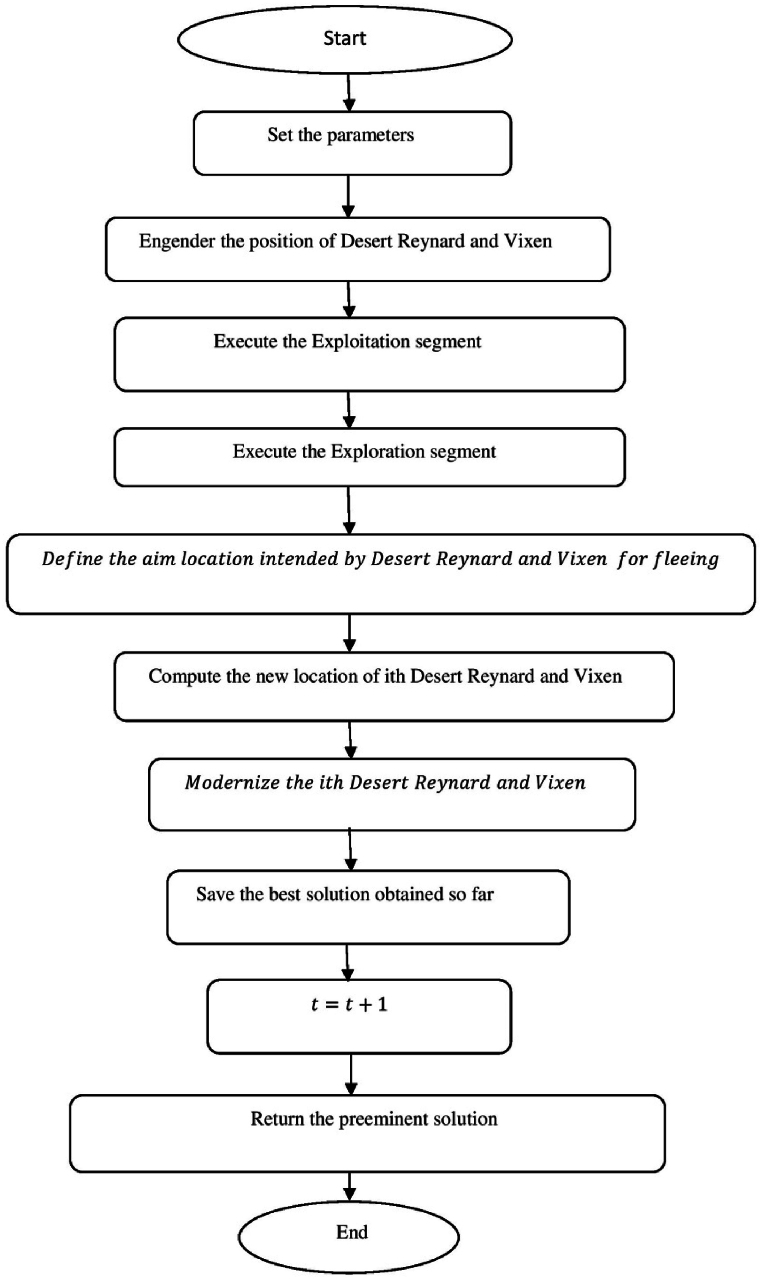
Flow chart of Desert Reynard and Vixen Inspired Optimization (DRVIO) Algorithm.

Fig. 3 shows the flow chart of Desert Reynard and Vixen Inspired Optimization (DRVIO) Algorithm.

a. Start
b. Set the parameter values
c. Engender the position of Desert Reynard and Vixen
d. Calculate the objective function
e. Fort=1:T:
f. Fori=1:N:
g. Execute the Exploitation segment
h. Compute the new location of ith Desert Reynard and Vixen
i. $z_{i,j}^{segment1}=z_{i,j}+\left(2*Random-1\right)*{Radius}_{i,j}$:
j. ${Radius}_{i,j}=\delta \cdot \left(1-\frac{t}{T}\right)\cdot z_{i,j}$:
k. ModernizetheithDesertReynardandVixen:
l. $Z_{i}=\left\{ \begin{array}{c} Z_{i}^{segment1},V_{i}^{segment1}<V_{i}\\ Z_{i},Else \end{array}\right.$:
m. Execute the Exploration segment
n. DefinetheaimlocationintendedbyDesertReynardandVixenforfleeing:
o. $Z_{i}^{Random}:z_{i,j}^{Random}=z_{k,j}$:
p. Compute the new location of ith Desert Reynard and Vixen
q. $Z_{i,j}^{Segment2}=\left\{ \begin{array}{c} z_{i,j}+random\cdot \left(z_{i,j}^{Random}-Q\cdot z_{i,j}\right),V_{i}^{random}<V_{i}\\ z_{i,j}+random\cdot \left(z_{i,j}-z_{i,j}^{Random}\right),Else \end{array}\right.$:
r. ModernizetheithDesertReynardandVixen:
s. $Z_{i}=\left\{ \begin{array}{c} Z_{i}^{segment2},V_{i}^{segment2}<V_{i}\\ Z_{i},Else \end{array}\right.$:
t. End for
u. t=t+1:
v. Output the best solution
w. End

## 6 Lonchodidae optimization algorithm

Then in this paper Lonchodidae optimization (LO) algorithm is applied to solve the problem. LO algorithm emulates the physiognomies of convergent progression, track reliance, populace development and rivalry in the growth of the Lonchodidae populace in environment. The Lonchodidae populace inclines to be the nearby leading populace in the development procedure, and encouraging progression drift is additional prospective to be congenital by the ensuing generation. This paper pools population development and rivalry replicas to attain the procedure.

The progression of the Lonchodidae population is diligently connected to the imitate alteration to the atmosphere. The development tendency of the Lonchodidae population is to alter with ecological variations and assimilate the aforementioned into the nearby surroundings. Consequently, the drive of the solution in the result region can be observed as the evolutionary drift of the Lonchodidae populace, and the alteration in fitness rate earlier and later the solution drive can be viewed as the alteration of the atmosphere. The Lonchodidae populace is pretentious by the ecological alteration and will yield the succeeding evolutionary drift. The subsequent evolutionary drift is pretentious by numerous physiognomies. By reviewing the evolution procedure ofLonchodidae, convergent progression, evolutionary track necessity, populace alteration, populace development and rivalry replicas are defined.

In convergent progression for populaces, analogous existing atmospheres are additional probable to yield analogous developments. Owing to the barricade of the topographical atmosphere, the development of the populace is nearer to the adjacent optima, but not essentially to the comprehensive optimal solution. The next is the track necessity in the progression of the populace. If the populace has improved endurance circumstances after progression, the populace will commonly endure the preceding progression drift. This is a comparatively toil tradable progression, but then again if the endurance circumstances turn out to be inferior next to the populace progresses, at that moment the populace may alter the evolutionary drift. Track necessity is analogous to the acquisitive procedure, which search for the succeeding location by enduring the preceding drive drift. The fundamental hypothesis is that the atmosphere fluctuations uninterestingly in a definite way. The track in this denotes to the evolutionary drift of the populace, and it inclines to endure the evolutionary track that attained improved consequences earlier. Every populace has double characteristics: populace amount and development degree. Populace rivalry and variations in the atmosphere will disturb the populace amount and development degree. While the populace of classes is not constrained by the atmosphere and possessions, its populace upsurges exponentially, but then again in the actual atmosphere, possessions and region are restricted, so the populace development prototype is repeatedly designated as follows,

(74) $$\frac{dQ}{dt}=aQ\left(1-\frac{Q}{E}\right)$$

Q→amountofpopulation

a→activeprogressiondegreeofpopulation

E→populationmaximumwithstandingcapabilityinatmosphericcondition

E=1

The amount of population is computed as,

(75) $$Q^{n+1}=bQ^{n}\left(1-Q^{n}\right)$$

Q∈[0,1]

b→developmentdegree

b∈[0,4]

whenb<1,populationdecreasing

1<b<3→convergetoavalueinstablemode

$$Q=\frac{\left(b-1\right)}{b}$$

b>3→populationwillbeinunstablemode

In accumulation to the influence of atmosphere and possessions on populace development, rivalry between dissimilar populaces will also disturb the amount and development degree of populaces and the deprived populaces may vanish.

(76) $$\frac{dQ}{dt}=a_{1}Q\left(1-\frac{Q}{o_{1}}-p_{1}\frac{R}{o_{2}}\right)$$

R→anotherpopulationquantity

$$a_{1}\to active\;progression\;degree\;of\;population$$

*o*_*1*_*→population ″ Q″ maximumwithstanding capability in atmospheric condition*

*o*_*2*_*→population ″ R″ maximumwithstanding capability in atmospheric condition*

$$p_{1}\to \;possessions$$

Randomly the population engendered as follows,

$$Q_{i}=\frac{1}{N_{pop}}$$

Past optima solution are stored as,

(77) $$z=\lfloor \log\left(N_{pop}\right)\rfloor +1$$

The position of the population is modernized as follows,

$$s^{n+1}=s^{n}+D$$

$$s^{n+1}\to new\;location$$

D→evolutionarytracknecessity

Initially update the amount of the population,

(78) $$Q^{n+1}=b^{n+1}Q^{n}\left(1-Q^{n}\right)$$

Then the population drift is streamlined as follows,

(79) $$D^{n+1}=\left(1-Q^{n+1}\right)H+Q^{n+1}\left(D^{n}+A\right)$$

H→rateofapproach

A→mutationfactor

$$H=\left(y\left(past,s^{t}\right)-s^{t}\right).g$$

g→influenceratecoefficient

g=0.2

$$y\left(past,s^{t}\right)\to used\;to\;obtain\;past\;optima\;solution$$

Based on the limits the population drift is updated as follows,

(80) $$D^{n+1}=Random\cdot H+\left(\max-\min\right)*0.1\cdot L$$

Random∈[0,1]

L→createdvector

maxandminarelimits

The rivalry between the populations is defined as,

(81) $$Q_{i}=Q_{i}+b_{i}Q_{i}\left(1-Q_{i}-\frac{f\left(s_{j}\right)}{f\left(s_{i}\right)}Q_{j}\right)$$

$$s_{i}\to present\;population$$

$$s_{j}\to randomly\;picked\;population$$

The space between tow populations is measured through an onset value as follows,

(82) $$M=0.1*\left(\max-\min\right)\frac{MG+1-n}{MG}$$

maxandminarelimits

MG→maximumgeneration

When the space between the present and randomly picked population is less than the onset value, then the progression drift is defined as follows,

(83) $$D^{n+1}=D^{n+1}+\frac{f\left(s_{j}\right)-f\left(s_{i}\right)}{f\left(s_{j}\right)}\left(s_{j}-s_{i}\right)$$

**Fig. 4:**
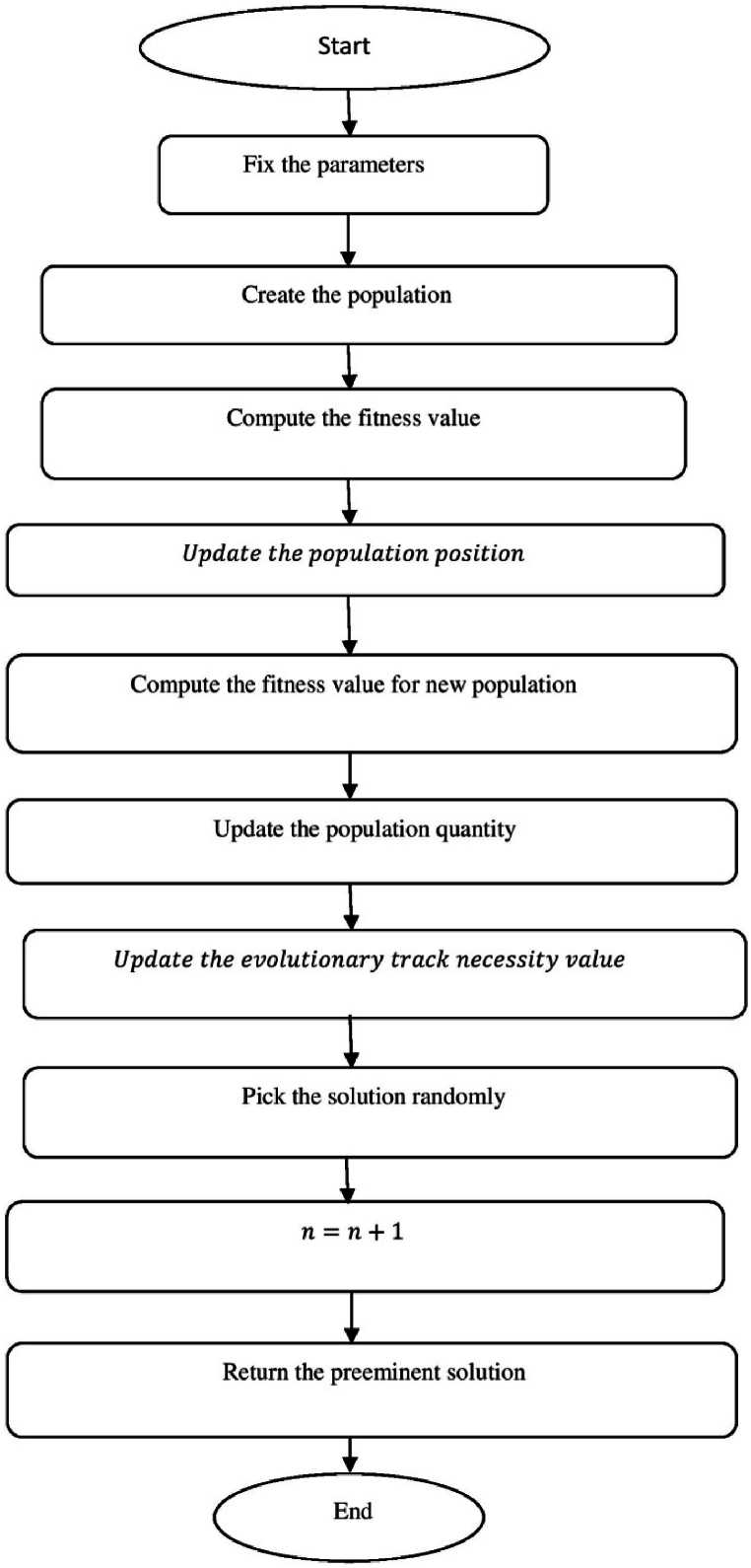
Flow chart of Lonchodidae optimization (LO) algorithm.

Fig. 4 show the flow chart of Lonchodidae optimization (LO) algorithm.

a. Start
b. Set the parameters
c. Create the population
d. $Q_{i}=\frac{1}{N_{pop}}$:
e. $z=\lfloor \log\left(N_{pop}\right)\rfloor +1$:
f. Calculate the fitness value
g. Fort=2toMGdo:
h. Updatethepopulationposition:
i. $s^{n+1}=s^{n}+D$:
j. Compute the fitness value for new population
k. $For\;i=1\;to\;N_{pop}\;do$:
l. iff(presents)≤f(s),then:
m. s=presents:
n. Update the population quantity
o. $Q^{n+1}=b^{n+1}Q^{n}\left(1-Q^{n}\right)$:
p. Updatetheevolutionarytracknecessityvalue:
q. $D^{n+1}=\left(1-Q^{n+1}\right)H+Q^{n+1}\left(D^{n}+A\right)$:
r. $H=\left(y\left(past,s^{t}\right)-s^{t}\right).g$:
s. Otherwise
t. $if\;ad<Q_{i},then$:
u. s=presents:
v. Update the population quantity
w. $Q^{n+1}=b^{n+1}Q^{n}\left(1-Q^{n}\right)$:
x. Updatetheevolutionarytracknecessityvalue:
y. $D^{n+1}=Random\cdot H+\left(\max-\min\right)*0.1\cdot L$:
z. Pick the solution randomly
aa. $if\;space\left(s_{j},s_{i}\right)<M,then$:
bb. Update $Q_{i}$:
cc. $Q_{i}=Q_{i}+b_{i}Q_{i}\left(1-Q_{i}-\frac{f\left(s_{j}\right)}{f\left(s_{i}\right)}Q_{j}\right)$:
dd. Updatetheevolutionarytracknecessityvalue:
ee. $D^{n+1}=D^{n+1}+\frac{f\left(s_{j}\right)-f\left(s_{i}\right)}{f\left(s_{j}\right)}\left(s_{j}-s_{i}\right)$:
ff. Endfor:
gg. n=n+1:
hh. Output the best solution
ii. End

## 7 Caracal optimization algorithm

Then in this paper Caracal optimization (CO) algorithm is applied to solve the problem. CO algorithm replicates the regular performance of Caracal in environment. The important stimulation of Caracal optimization algorithm is the stalking scheme; Caracal assaults the designated quarry and then quests the quarry in a dashing procedure. Caracal is an expert hunter that quests its quarry in three phases. Principally, by means of its robust intelligence of earshot, it recognizes the location of the quarry and detects and observes the quarry for more than 16 min deprived of any sort of movement. In the second phase Caracal passages in the direction of the quarry, hurdles up in the air and bouts the quarry. In last phase dashing procedure by running and hurdling to clasp the absconding quarry, the Caracalmurders it and consumes it. These strategies are imitated to formulate the CO algorithm.

(84) $$C={\left[\begin{array}{c} C_{1}\\ \vdots \\ C_{i}\\ \vdots \\ C_{N} \end{array}\right]}_{N\times D}={\left[\begin{array}{ccc} c_{1,1} & \cdots & c_{1,d}\\ \vdots & \ddots & \vdots \\ c_{N,1} & \cdots & c_{N,d} \end{array}\right]}_{N\times d}$$

C→Caracalpopulation

(85) $$c_{i,j}=\min_{j}+{random}_{i,j}\cdot \left(\max_{j}-\min_{j}\right)$$

$${random}_{i,j}\in \left[0,1\right]$$

$$\max_{j}and\;\min_{j}\;are\;the\;limits$$

$$c_{i,j}\to examination\;segment\;dimension$$

i=1,2,3,4,..,N

j=1,2,3,4,..,d

(86) $$D={\left[\begin{array}{c} D_{1}\\ \vdots \\ D_{i}\\ \vdots \\ D_{N} \end{array}\right]}_{N\times 1}={\left[\begin{array}{c} D\left(C_{1}\right)\\ \vdots \\ D\left(C_{i}\right)\\ \vdots \\ D\left(C_{N}\right) \end{array}\right]}_{N\times 1}$$

D→valueoftheobjectivefunction

In exploration phase, Caracal utilizes its robust intelligence of earshot to recognize the position of its quarry and assault it. Initially the locations of Caracals are rationalized grounded on the imitation of these twofold schemes. This modernization grounds large alterations in the location of Caracals and tips to a comprehensive look over in examination region. Location of the population's preeminent associate is measured as the quarry location.

(87) $$c_{i,j}^{A1}=c_{i,j}+{random}_{i,j}\cdot \left(Q_{j}-B_{i,j}\cdot c_{i,j}\right)$$

$${random}_{i,j}\in \left[0,1\right]$$

$$c_{i,j}\to examination\;segment\;dimension$$

i=1,2,3,4,..,N

j=1,2,3,4,..,d

A1→initialposition

(88) $$C_{i}=\left\{ \begin{array}{c} C_{i}^{A1},D_{i}^{A1}<D_{i}\\ D_{i},otherwise \end{array}\right.$$

$$c_{i,j}^{A1}\to location\;of\;Caracal\;in\;initial\;phase$$

$$D_{i}^{A1}\to objective\;functional\;value$$

$$Q_{j}\to \;dimension\;of\;the\;quarry\;position$$

$$B_{i,j}\in \left\{1,2\right\}$$

In the exploitation phase, subsequent to confronting the quarry, the Caracal attempts to halt the quarry by hurdling in a pursuit method, then Caracalmurder the quarry and consume it. This aspect has been imitated to update the location of the Caracal. The simulation of the chase process causes small changes in the positions of the Caracals in the search space.

(89) $$c_{i,j}^{A2}=c_{i,j}+\frac{{random}_{i,j}\cdot \left(\max_{j}-\min_{j}\right)}{t}$$

$${random}_{i,j}\in \left[0,1\right]$$

$$c_{i,j}\to examination\;segment\;dimension$$

i=1,2,3,4,..,N

j=1,2,3,4,..,d

A2→nextposition

(90) $$C_{i}=\left\{ \begin{array}{c} C_{i}^{A2},D_{i}^{A2}<D_{i}\\ D_{i},otherwise \end{array}\right.$$

$$c_{i,j}^{A2}\to location\;of\;Caracal$$

$$D_{i}^{A2}\to objective\;functional\;value$$

t=1,2,3,4,..,T

tandT→iterationcounterandsumofiterations

Through modernizing all Caracal grounded on the principal and subsequent parts of CO, the initial iteration of the procedure is accomplished. Grounded on the fresh locations of the Caracal and novel standards attained, the procedure passes into next iteration.

**Fig. 5:**
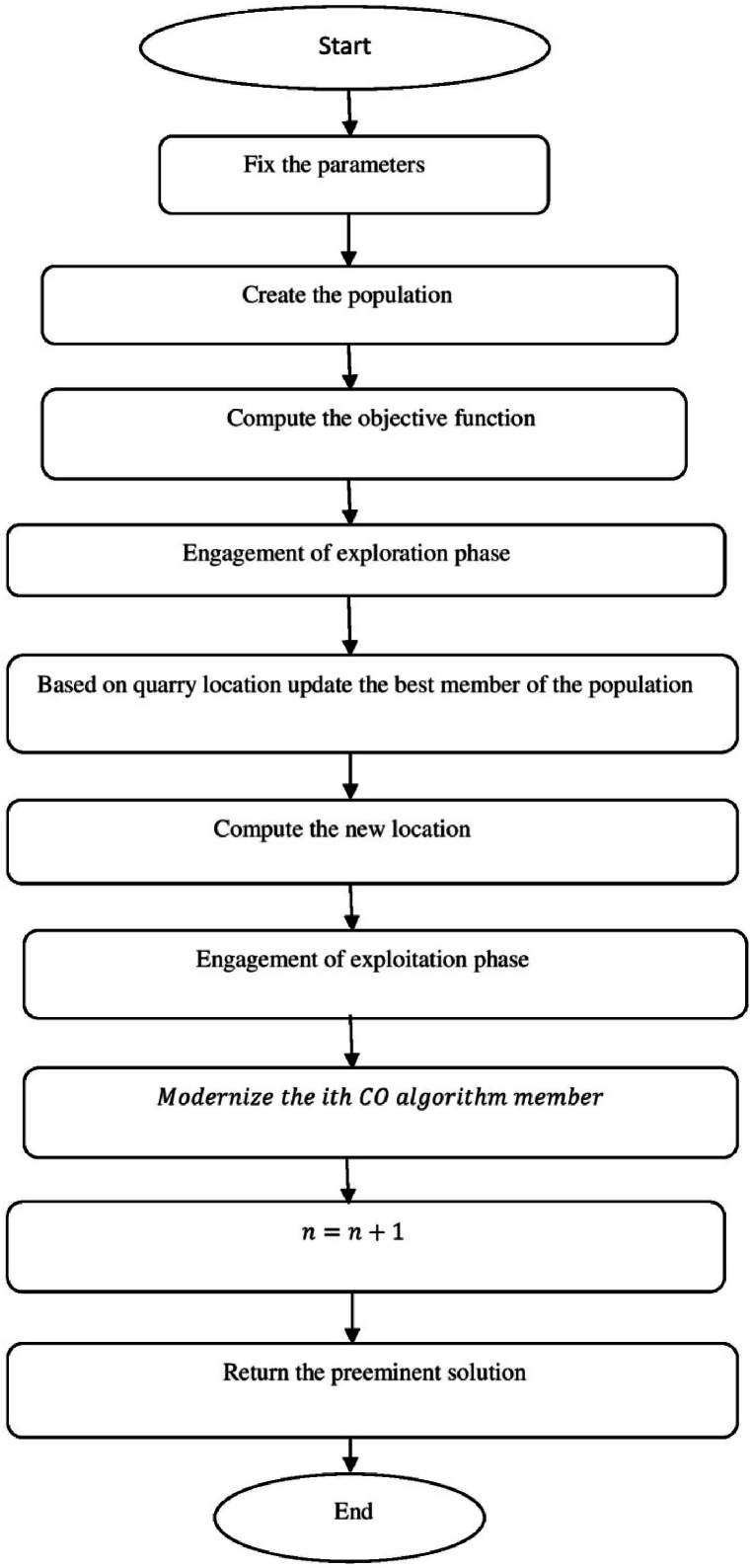
Flowchart of Caracal optimization (CO) algorithm.

Fig. 5 show flowchart of Caracal optimization (CO) algorithm.

a. Start
b. Set the parameters
c. Create the population
d. Compute the objective the function
e. Fort=1toN:
f. Fori=1toN:
g. Engagement of exploration phase
h. Based on quarry location update the best member of the population
i. Compute the new location
j. $c_{i,j}^{A1}=c_{i,j}+{random}_{i,j}\cdot \left(Q_{j}-B_{i,j}\cdot c_{i,j}\right)$:
k. ModernizetheithCOalgorithmmember:
l. $C_{i}=\left\{ \begin{array}{c} C_{i}^{A1},D_{i}^{A1}<D_{i}\\ D_{i},otherwise \end{array}\right.$:
m. Engagement of exploitation phase
n. $c_{i,j}^{A2}=c_{i,j}+\frac{{random}_{i,j}\cdot \left(\max_{j}-{min}_{j}\right)}{t}$:
o. ModernizetheithCOalgorithmmember:
p. $C_{i}=\left\{ \begin{array}{c} C_{i}^{A2},D_{i}^{A2}<D_{i}\\ D_{i},otherwise \end{array}\right.$:
q. End while
r. t=t+1:
s. Output the best solution
t. End

## 8 Barasingha optimization algorithm

Then in this paper Barasingha optimization (BO) algorithm is applied to solve the problem. BO algorithm stimulated by the Barasingha existence capability in the slayer subjugated atmosphere. Each day, the Barasingha discerns that if it does not outpace and outwit its slayers, it turn out to be victim of the day for the slayer. Even with their being downcast in the nutriment cable, they are not categorized as scarce; this indicates that Barasingha is performing approximately correct. Mainly Barasingha will spurt from slayers. In the projected BO approach the Exploration segment of the procedure, the Barasingha is foraging peaceably in the absenteeism of the slayer or even though the slayer is trailing it. BO drives into the exploitation segment as soon as a slayer is mottled, it entails of the Barasingha outpacing and outwitting the slayer to an anchorage.

Population of Barasingha is engendered as follows,

(91) $$Z=\left[\begin{array}{ccc} z_{1,1} & \cdots & z_{1,d}\\ \vdots & \ddots & \vdots \\ z_{n,1} & \cdots & z_{n,1} \end{array}\right]$$

Z→presentpopulation

n→sumofcandidatepopulation

(92) $$z_{i,j}=R\cdot \left(\max_{j}-\min_{j}\right)+\min_{j}$$

R→random∈[0,1]

$$\max_{j}and\;\min_{j}\;are\;the\;limits$$

The finest attained solution, hitherto, is considered as the optima around in iterations. Naturally resilient Barasingha in environment is extra brilliant in noticing, expressing to other Barasinghas of the delicacies and flee away from slayers. Consequently, the fitting solution is designated as a best Barasingha to create a medium named as Leading Barasingha. This medium is used for examining and discovery of the subsequent phase for theBarasingha.

(93) $$Leading\;Barasingha=\left[\begin{array}{ccc} z_{1,1}^{\prime } & \cdots & z_{1,d}^{\prime }\\ \vdots & \ddots & \vdots \\ z_{n,1}^{\prime } & \cdots & z_{n,d}^{\prime } \end{array}\right]$$

Both the slayer and victim as considered as search agents. Since by the period a slayer is mottled trailing the Barasingha, then both run in the similar track in the direction of the harmless anchorage, and while the Barasingha spurt out, the slayer will have also discovered the exploration region. At the end iterations, the Leading Barasingha will be rationalized if the enhanced Barasingha substitutes the Leading Barasingha. Then the phase length is haggard from the Gaussian probability [48] as follows,

(94) $$G_{k}\left(b\right)=\frac{1}{\sqrt{{\left(2\pi \right)}^{2}\left|C_{k}\right|}}\;\exp\;{\left(-\frac{1}{2}\left(b-\mu_{k}\right)\right)}^{T}\cdot C_{k}^{-1}\left(b-\mu_{k}\right)$$

$$\sum_{k=1}^{k}\alpha_{k}=1$$

$$Q\left(b\right)\approx \sum_{k=1}^{k}\alpha_{k}\cdot G_{k}\left(b\right)$$

Then the Lévy flight [49] is utilized for the arbitrary walking,

(95) L(s)∼|s|−1−βwhere0<ß<2

(96) $$L\left(s,\gamma ,\mu \right)=\left\{ \begin{array}{c} \sqrt{\frac{\gamma }{2\pi }}\\ 0\;\text{if}\;s\leq 0\; \end{array}\right.\exp\left[-\frac{\gamma }{2\left(s-\mu \right)}\right]\frac{1}{{\left(s-\mu \right)}^{3/2}}\;\text{if}\;0\;<\mu <s<\infty$$

(97) $$F\left(k\right)=\exp\left[-\alpha {\left|k\right|}^{\beta }\right]\;0<\beta \leq 2$$

(98) $$Z^{t+1}=Z^{t}+R\;\left(s\left(D\right)\right)⊕\text{Levy}\left(\beta \right)∼0.01\frac{u}{{\left|v\right|}^{1/\beta }}\left(Z_{j}^{t}-\text{gb}\right)$$

In the segment of Exploitation the Barasingha are presumed as foraging peaceably in the absenteeism of a slayer or even though the slayer is trailing the Barasingha. In this segment, Brownian motion is utilized by unvarying and exact phases to efficiently conceal of neighbourhood zones of the realm.

(99) $$\vec{{BB}_{t+1}}=\vec{{BB}_{t}}+g\cdot \vec{O}*\vec{O_{BM}}**\left(\vec{{LeadingBB}_{t}}-\vec{O_{BM}}\cdot \vec{{BB}_{t}}\right)$$

BB→Barasingha

$$\vec{{BB}_{t+1}}\;indicate\;the\;subsequent\;iteration\;solution$$

$$\vec{{BB}_{t}}\;is\;the\;present\;iteration\;solution$$

$$\vec{O_{BM}}\;is\;the\;random\;number\;of\;Brownian\;motion$$

O→random∈[0,1]

g→foragingrapidnessofBarasinghas

The exploration segment will be initiated once the slayer is near-sighted. The Barasingha respond to risk by riffle their tail, trudge their feet or espouse a jumping elegance as bouncing into the air of height considered as amongst 0 and 1. Lévy flight utilized in this segment of the technique, the procedure entails of captivating minute stages and infrequently extended jump. The Barasingha responds principally, and it runs by means of Lévy flight. The slayer responds after this, and it run through Brownian motion beforehand altering to Lévy flight future stage.

(100) $$\vec{{BB}_{t+1}}=\vec{{BB}_{t}}+G\cdot \sigma \cdot \vec{O}*\vec{O_{L}}*\left(\vec{{LeadingBB}_{t}}-\vec{O_{L}}\cdot \vec{{BB}_{t}}\right)$$

BB→Barasingha

$$\vec{{BB}_{t+1}}\;indicate\;the\;subsequent\;iteration\;solution$$

$$\vec{{BB}_{t}}\;is\;the\;present\;iteration\;solution$$

$$\vec{O_{L}}\;is\;the\;random\;number\;of\;Levy$$

O→random∈[0,1],σ→movementofdirection

G→highestrapidmovementwhichBarasinghascanbeattained

The slayer chasing the Barasingha is defined as,

(101) $$\vec{{BB}_{t+1}}=\vec{{BB}_{t}}+G\cdot \sigma \cdot M*\vec{O_{BM}}*\left(\vec{{LeadingBB}_{t}}-\vec{O_{L}}\cdot \vec{{BB}_{t}}\right)$$

BB→Barasingha

$$\vec{{BB}_{t+1}}\;indicate\;the\;subsequent\;iteration\;solution$$

$$\vec{{BB}_{t}}\;is\;the\;present\;iteration\;solution$$

$$\vec{O_{BM}}\;is\;the\;random\;number\;of\;Brownian\;motion$$

$$\vec{O_{L}}\;is\;the\;random\;number\;of\;Levy$$

O→random∈[0,1],σ→movementofdirection

G→highestrapidmovemnetwhichBarasinghascanbeattained

M→Movementoftheslayer

(102) $$M={\left(1-\frac{iteration}{\max\;imum\;iteration}\right)}^{\left(2\frac{iteration}{maximum\;iteration}\right)}$$

Slayer attainment rate, the consequence marks the capability of the Barasingha to spurt out; it indicates the BO algorithm evades being stuck in a local minima.

(103) $$\vec{{BB}_{t+1}}=\left\{ \begin{array}{c} \vec{{BB}_{t}}+M\left[R\cdot \left(\max_{j}-\min_{j}\right)+\min_{j}\right]*\vec{P},if\;o\leq S\\ \vec{{BB}_{t}}+\left[S\left(1-o\right)+o\right]\left(\vec{{BB}_{o1}}-\vec{{BB}_{o1}}\right),Else \end{array}\right.$$

S→Slayerattainmentrate

o∈[0,1]R→random∈[0,1]

$$\max_{j}and\;\min_{j}\;are\;the\;limits,\vec{P}\to binary\;vector$$

M→Movementoftheslayer,BB→Barasingha

$$\vec{{BB}_{t+1}}\;indicate\;the\;subsequent\;iteration\;solution$$

$$\vec{{BB}_{t}}\;is\;the\;present\;iteration\;solution$$

(104) $$\vec{P}=\left\{ \begin{array}{c} 0,if\;o<0.20\\ 1,Else \end{array}\right.$$

**Fig. 6:**
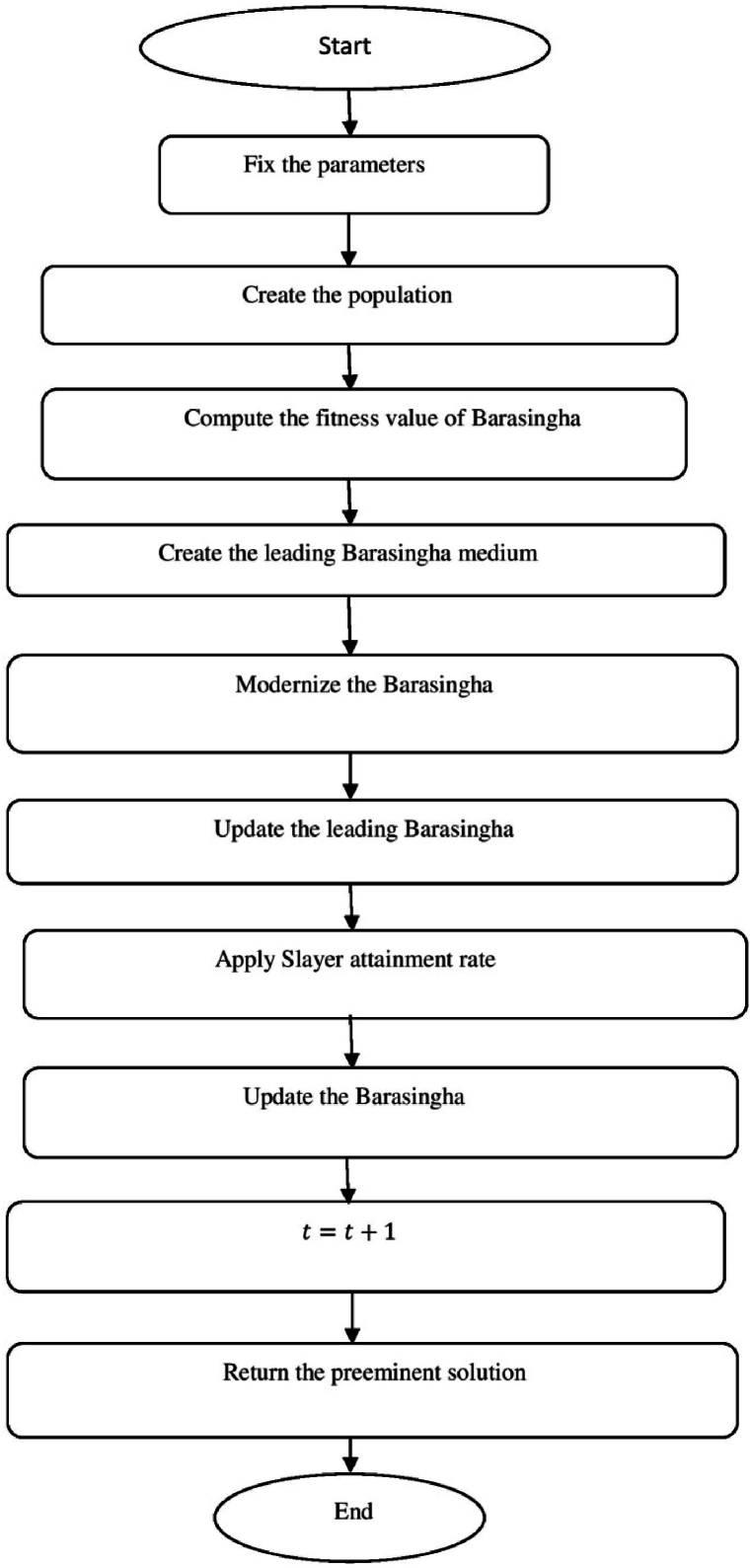
Flow chart of Barasingha optimization (BO) algorithm.

Fig. 6 shows the flow chart of Barasingha optimization (BO) algorithm.

a. Start
b. Set the parameters
c. Create the population
d. whileiteration<maximumiteration:
e. Compute the fitness value of Barasingha
f. Create the leading Barasingha medium
g. ifo<2,then:
h. Modernize the Barasingha
i. $\vec{{BB}_{t+1}}=\vec{{BB}_{t}}+g\cdot \vec{O}*\vec{O_{BM}}**\left(\vec{{LeadingBB}_{t}}-\vec{O_{BM}}\cdot \vec{{BB}_{t}}\right)$:
j. Otherwsie:
k. Ifmod(iteration,2)==0,then:
l. σ=−1:
m. Otherwise
n. σ=1:
o. Update the Barasingha population
p. $\vec{{BB}_{t+1}}=\vec{{BB}_{t}}+G\cdot \sigma \cdot \vec{O}*\vec{O_{L}}*\left(\vec{{LeadingBB}_{t}}-\vec{O_{L}}\cdot \vec{{BB}_{t}}\right)$:
q. BasedtheslayerupdatethepopulationofBarasingha:
r. $\vec{{BB}_{t+1}}=\vec{{BB}_{t}}+G\cdot \sigma \cdot M*\vec{O_{BM}}*\left(\vec{{LeadingBB}_{t}}-\vec{O_{L}}\cdot \vec{{BB}_{t}}\right)$:
s. Endif:
t. Update the leading Barasingha
u. Apply Slayer attainment rate
v. Update the Barasingha
w. $\vec{{BB}_{t+1}}=\left\{ \begin{array}{c} \vec{{BB}_{t}}+M\left[R\cdot \left(\max_{j}-\min_{j}\right)+\min_{j}\right]*\vec{P},if\;o\leq S\\ \vec{{BB}_{t}}+\left[S\left(1-o\right)+o\right]\left(\vec{{BB}_{o1}}-\vec{{BB}_{o1}}\right),Else \end{array}\right.$:
x. End while
y. t=t+1:
z. Output the best solution
aa. End

## 9 Amur leopard optimization algorithm

Then in this paper Amur leopard optimization (AO) algorithm is applied to solve the problem. Proposed AO algorithm imitates the Amur leopard behaviour. Movement paths, stalking, breeding and death are the some phases in the Amur leopard life cycle. These phases are mathematically designed in the projected algorithm.

Each Amur leopard is an associate of the procedure population. An assured quantity of Amur leopard acts as examination agents are associates of theAO algorithm. The population matrix of the AO algorithm is methodically defined as,

(105) $$A={\left[\begin{array}{c} A_{1}\\ \vdots \\ A_{i}\\ \vdots \\ A_{N} \end{array}\right]}_{N\times D}={\left[\begin{array}{ccc} a_{1,1} & \cdots & a_{1,d}\\ \vdots & \ddots & \vdots \\ a_{N,1} & \cdots & a_{N,d} \end{array}\right]}_{N\times d}$$

A→Amurleopardpopulation

(106) $$a_{i,d}={LB}_{d}+R_{i,d}\cdot \left({UB}_{d}-{LB}_{d}\right)$$

$$R_{i,d}\in \left[0,1\right]$$

$${UB}_{d}and{LB}_{d}\;\to lower\;and\;upper\;limits$$

The rate of the key objective functional value is defined as,

(107) $$B={\left[\begin{array}{c} B_{1}\\ \vdots \\ B_{i}\\ \vdots \\ B_{N} \end{array}\right]}_{N\times 1}={\left[\begin{array}{c} B\left(O_{1}\right)\\ \vdots \\ B\left(O_{i}\right)\\ \vdots \\ B\left(O_{N}\right) \end{array}\right]}_{N\times 1}$$

B→obj.functionvalue

The movement of the Amur leopard is defined as,

(108) $$a_{i,d}^{p1}=a_{i,d}+R_{i,d}\cdot \left(a_{k,d}-E\cdot a_{i,d}\right)*sign\left(B_{i}-B_{k}\right)$$

d=1,2,3,4,..,m

k=1,2,3,4,..,N

(109) $$A_{i}=\left\{ \begin{array}{c} A_{i}^{P1},B_{i}^{P1}<B_{i}\\ A_{i},Else \end{array}\right.$$

$$a_{i}^{P1}\to position\;of\;Amur\;leopard\;in\;movement\;phase$$

$$R_{i,d}\in \left[0,1\right]$$

$$A_{i}^{P1}\to updated\;position\;in\;the\;movement\;phase$$

$$B_{i}^{P1}\to obj.\;functional\;value$$

E=round(1+r)

In the subsequent segment of modernizing the associates of the population, the activities of Amur leopard during stalking and confronting the prey are utilized. Fresh location of the Amur leopard subsequentto the bout on the prey is replicated. An active modernization is used in which the fresh location is adequate to the procedure associate if the rate of the objective function in the fresh location is extra suitable than the preceding location.

(110) $$p_{i,d}=a_{i,d},d=1,2,3,4,..,m$$

(111) $$a_{i,d}^{p2}=a_{i,d}+R_{i,d}*\left(\left(p_{i,d}-a_{i,d}\right)*G+\left(p_{i,d}-{2*a}_{i,d}\right)*\left(1-G\right)\right)*\;sign\left(B_{i}-B_{k}\right)$$

$$a_{i}^{P2}\to position\;of\;Amur\;leopard\;in\;stalking\;phase,G\to location$$

(112) $$A_{i}=\left\{ \begin{array}{c} A_{i}^{P2},B_{i}^{P2}<B_{i}\\ A_{i},Else \end{array}\right.$$

$$B_{i}^{P2}\to obj.\;functional\;value$$

In breeding segment, grounded on the regular generative performance ofAmur leopard, fresh associates equivalent to partial the entire population are included to the populace of the procedure.

(113) $$O_{l}=\frac{a_{l}+A_{N-l+1}}{2}$$

$$l=1,2,3,4,..,\frac{N}{2}$$

$$O_{l}\to is\;the\;offsprings\;;\;produced\;by\;mating\;of\;male\;and\;female\;Amur\;leopard$$

In the death segment, even though reproduction upsurges the populace of Amur leopard and it remnants constant mode throughout the reproduction of the procedure owing to death. In the projectedAO, it is presumed that in every imitation after reproduction, Amur leopard face death accurately as the quantity of offspring's. The condition of Amur leopard death in the AO procedure is the rate of the objective functional value. Consequently, Amur leopard which possess weedier objective functional value are more inclined to demise. Likewise, few offspring's might expire owing to having deprived objective functional value.

**Fig. 7:**
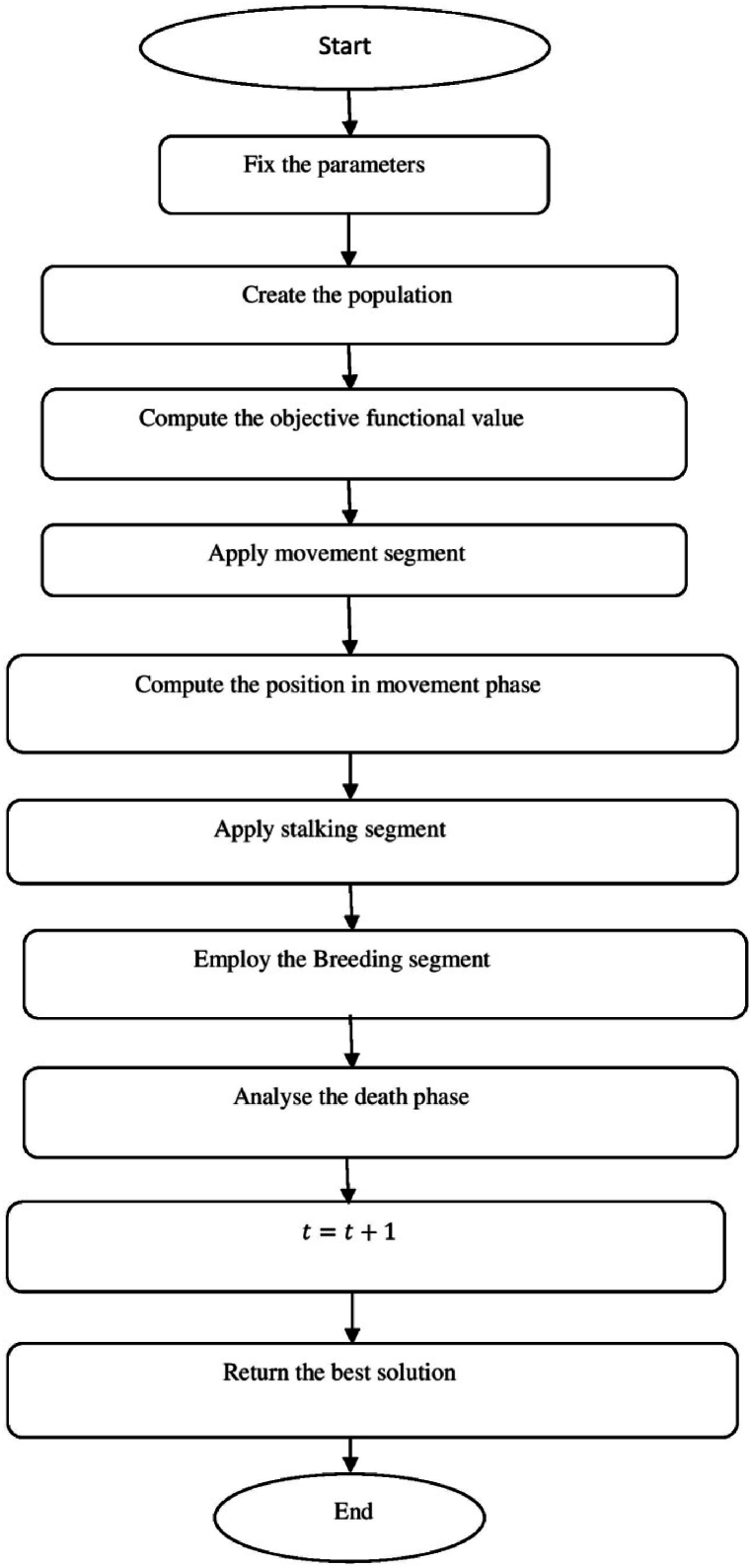
Flow chart of Amur leopard optimization (AO) algorithm.

Fig. 7 show the flow chart of Amur leopard optimization (AO) algorithm.

a. Start
b. Set the parameters
c. Create the preliminary population
d. Calculate the objective functional value
e. Fort=1:T:
f. Apply movement segment
g. Fori=1:N:
h. Ford=1:m:
i. Compute position of Amur leopard in movement phase
j. $a_{i,d}^{p1}=a_{i,d}+R_{i,d}\cdot \left(a_{k,d}-E\cdot a_{i,d}\right)*sign\left(B_{i}-B_{k}\right)$:
k. E=round(1+r):
l. End
m. Update $A_{i}$:
n. $A_{i}=\left\{ \begin{array}{c} A_{i}^{P1},B_{i}^{P1}<B_{i}\\ A_{i},Else \end{array}\right.$:
o. End
p. Apply stalking segment
q. Fori=1:N:
r. Ford=1:m:
s. Compute the position of the prey
t. $p_{i,d}=a_{i,d}$:
u. Compute the position of Amur leopard in stalking phase
v. $a_{i,d}^{p2}=a_{i,d}+R_{i,d}*\left(\left(p_{i,d}-a_{i,d}\right)*G+\left(p_{i,d}-{2*a}_{i,d}\right)*\left(1-G\right)\right)*\;sign\left(B_{i}-B_{k}\right)$:
w. End:
x. Update $A_{i}$:
y. $A_{i}=\left\{ \begin{array}{c} A_{i}^{P2},B_{i}^{P2}<B_{i}\\ A_{i},Else \end{array}\right.$:
z. End
aa. Employ the Breeding segment
bb. Forl=1:0.5×N:
cc. Engender the offspring
dd. $O_{l}=\frac{a_{l}+A_{N-l+1}}{2}$:
ee. End:
ff. Analyse the death phase
gg. Regulate the quantity of Amur leopard to N owing to death grounded on condition of the objective functional value
hh. End while
ii. t=t+1:
jj. Output the best solution
kk. End

## 10 Empress SARANI optimization algorithm

Empress SARANI Optimization Algorithm is designed by integrating Parastylotermes Empress inspired optimization (PEIO) algorithm, Dryocopus martius optimization (DMO) algorithm, Ostrya Carpinifolia Search Optimization (OCSO) Algorithm with SARANI algorithm. Sine -Cosine Optimization Algorithm (SCA), Red Dhole Optimizer Algorithm (ROA), North Sumatra Island Pongo abelii optimization Algorithm (NIA), Dryocopus martius optimization (DMO) algorithm are integrated and entitled as SARANI algorithm.

SARANI algorithm uniting functions of sine-cosine, antagonizing feint of Red Dhole Optimizer Algorithm with North Sumatra Island Pongo abelii optimization algorithm will act as initial step of the method.Primarily male Dryocopus martius will be in gargantuan number and in the length of the initial phase of association the sum of male Dryocopus martius reduces due to association. As soon as iteration upsurges exactly the population reduces and through this exploration and exploitation is balanced. Dryocopus martius optimization (DMO) algorithm features are integrated into the algorithm to balance the exploration and exploitation. In the combined SARANI approach NIA steps move in the way of spotting the optimum solution and reformed stages are smeared to accomplish the optimal in the exploration zone. This passageway has simplified promptly to complete the leading global optimal solution. At first population is primed arbitrarily. In the Valuation segment population discover the members and it appraised by the domination of the exploration planetary in the examination method. At that minute the fitness rate of every exploration companion is verified by the providing practical value and in the development of the exploration and each exploration assistant of the carton disbursements these fitness canons to location the modern examination locations. Grounded on the functions of Sine -Cosine an algorithm is sculpted and it titled as Sine Cosine Optimization Algorithm (SCA).

(114) $${\vec{\text{SC}}}_{i}^{m+1}={\vec{\text{SC}}}_{i}^{m}+R_{1}\times \mathrm{Sin}\left(R_{2}\right)\times \left|R_{3}\times E_{i}^{m}-{\vec{\text{SC}}}_{i}^{m}\right|$$

(115) $${\vec{\text{SC}}}_{i}^{m+1}={\vec{\text{SC}}}_{i}^{m}+R_{1}\times \mathrm{Cos}\left(R_{2}\right)\times \left|R_{3}\times E_{i}^{m}-{\vec{\text{SC}}}_{i}^{m}\right|$$

(116) $${\vec{\text{SC}}}_{i}^{m+1}=\left\{ \begin{array}{c} {\vec{\text{SC}}}_{i}^{m}+R_{1}\times \mathrm{Sin}\left(R_{2}\right)\times \left|R_{3}\times E_{i}^{m}-{\vec{\text{SC}}}_{i}^{m}\right|R_{4}<0.50\\ {\vec{\text{SC}}}_{i}^{m}+R_{1}\times \mathrm{Cos}\left(R_{2}\right)\times \left|R_{3}\times E_{i}^{m}-{\vec{\text{SC}}}_{i}^{m}\right|R_{4}\geq 0.50 \end{array}\right.$$

$$\text{where}\;{\vec{\text{SC}}}_{i}^{m}\;\text{is}\;\text{the}\;\text{prsent}\;\text{position}\;\text{at}\;\text{mth}\;\text{iteration}\;\text{with}{\;E}_{i}^{m}\;\text{population}$$

Red Dhole Optimizer Algorithm (ROA) derived from normal activities of Red Dhole which is deceitful and highbrow. When a garden-fresh nourishment spring is found, then Red Dhole harvests an exemplary tittering sound to interrelate about the nourishment spring discoveries. Pairing between Red Dhole encompass an expanse of minute intercourse within trifling intermezzos. Red Dhole cubs are congenital circa with matured configurations. Red Dhole does not reaffirm nourishment for their cubs and for acclaim Red Dhole exploit plentiful sensual progressions with that pronouncement creation will be completed. Methodical model of encircling the prey is designated as,

(117) $${\vec{\text{Hb}}}_{T}=\left|\vec{Q\;}\;.\;{\vec{R}}_{p}\left(z\right)-\vec{R\;}\;\left(z\right)\right|$$

(118) $$\vec{R\;}\left(z+1\right)={\vec{R}}_{p}\left(z\right)-\vec{G}\;.{\vec{\text{Hb}}}_{T}$$

$$\text{where}\;{\vec{\text{Hb}}}_{T}\;\text{symbolize}\;\text{the}\;\text{distance}\;\text{of}\;\text{Red}\;\text{Dhole}\;\text{with}\;\text{prey}$$

$${\vec{R}}_{p}\;\text{specify}\;\text{the}\;\text{location}\;\text{of}\;\text{the}\;\text{prey}$$

$$\vec{R\;}\;\text{is}\;\text{the}\;\text{spot}\;\text{of}\;\text{the}\;\text{Red}\;\text{Dhole}$$

$$\vec{Q\;}\;\text{and}\vec{G}\;\text{are}\;\text{Coefficient}\;\text{vectors}$$

(119) $$\vec{Q}=2.\vec{z}{\vec{d}}_{1}$$

(120) $$\vec{G}=2\vec{T}\;.\vec{z}{\vec{d}}_{2}-\vec{T}$$

(121) $$\vec{T}=5-\left(\text{iter}\;X\frac{5}{\mathrm{Max}\;\text{iter}}\right)$$

whereIter.=1,2,3,...,Max.Iter.

$$\vec{z}d_{1}\;\text{and}\;\vec{z}{\vec{d}}_{2}\in \left[0,1\right]$$

Stalking procedure is methodically demarcated as,

(122) $${\vec{\text{Hb}}}_{T}=\left|\vec{Q\;}\;.\;R_{T}-{\vec{R}}_{S}\right|$$

(123) $${\vec{R}}_{S}={\vec{R}}_{T}-\vec{G}\;.{\vec{\text{Hb}}}_{T}$$

(124) $${\vec{O}}_{T}={\vec{R}}_{S}+{\vec{R}}_{S+1}+..+{\vec{R}}_{S+N}$$

whereNspecifythenumberofiterations

(125) $$N=C_{N}\left({\vec{R}}_{T},{\vec{R}}_{T+1},\ldots \left({\vec{R}}_{T}+\vec{V}\right)\right)$$

$$\text{Where}\;\vec{V}\;\text{is}\;\text{an}\;\text{arbitrary}\;\text{vector}\in \left[0.5,1\right]$$

(126) $$\vec{R}\left(z+1\right)=\frac{{\vec{O}}_{T}}{N}$$

$$\text{where}\;\vec{R}\left(z+1\right)\;\text{used}\;\text{for}\;\text{streamline}\;\text{the}\;\text{solutions}$$

$$\vec{V}\;\text{specify}\;\text{the}\;\text{randomly}\;\text{initilized}\;\text{vactors}$$

$${\vec{O}}_{T}\;\text{indicate}\;\text{the}\;\text{solution}\;\text{clusters}$$

$$\vec{G}\;\text{regulate}\;\text{the}\;\text{exploration}$$

$$\vec{Q\;}\;\text{assist}\;\text{the}\;\text{exploration}$$

weight∈[0,5]

$$\text{when}\;\vec{G}<1\;exploitation\;starts\in \left[-1,1\right]$$

North Sumatra Island Pongo abelii optimization algorithm (NIA) is sculpted grounded on the distinct acumen and sensual inducement in group. Preliminary solution is anticipated to be heightened and acquainted of the scene of the objective by the accoster, hitch, trailer and teamster. In the successive segment four supplementary things are still accomplished optimum solutions are hoarded and the additional North Sumatra Island Pongo abelii are obligatory to revolutionize their specific location to the dominant places of Pongo abelii. Methodical model of the North Sumatra Island Pongo abelii optimization algorithm (NIA) is demarcated as,

(127) $$P_{s}=\left|a\;.\;b_{p}\left(t_{i}\right)-{e.b}_{P}\left(t_{i}\right)\right|$$

(128) $$b_{P}\left(t_{i}+1\right)=b_{p}-b.u$$

$$\text{where}\;t_{i}\;\text{specify}\;\text{the}\;\text{count}\;\text{of}\;\text{iterations}$$

a,eandbarecoefficients

(129) $$b=2.c.\;F_{1}-c$$

(130) $$a=2.F_{2}$$

$$F_{1},F_{2}\in \left[0,1\right]$$

c→2.5to0

(131) $$P_{\text{accoster}}=\left|a_{1}\;\cdot b_{\text{accoster}}-e_{1}.x\right|$$

(132) $$P_{\text{hitch}}=\left|a_{2}\cdot \;b_{\text{hitch}}-e_{2}.x\right|$$

(133) $$P_{\text{trailer}}=\left|a_{3}\cdot \;b_{\text{trailer}}-e_{2}.x\right|$$

(134) $$P_{\text{teamster}}=\left|a_{4}\cdot b_{\text{teamster}}-e_{4}.x\right|$$

Succeeding position of the North Sumatra Island Pongo abelii demarcated as follows,

(135) $$x_{1}=b_{\text{accoster}}-b_{1}.d_{\text{accoster}}$$

(136) $$x_{2}=b_{\text{hitch}}-b_{2}.d_{\text{hitch}}$$

(137) $$x_{3}=b_{\text{trailer}\;}-b_{3}.d_{\text{trailer}\;}$$

(138) $$x_{4}=b_{\text{teamster}}-b_{4}.d_{\text{teamster}}$$

Throughout the exploration the North Sumatra Island Pongo abelii change the location and it designated as,

(139) $$x\left(t_{i}+1\right)=\frac{x_{1}+x_{2}+x_{3}+x_{4}}{4}$$

Then the position of the North Sumatra Island Pongo abelii is modernized by,

(140) $$b_{P}\left(t_{i}+1\right)=\left\{ \begin{array}{c} b_{p}\left(t_{i}\right)-x.d,\text{if}\emptyset <0.5\\ \;\text{Ch},\text{if}\;\text{if}\emptyset >0.5 \end{array}\right.$$

Extreme learning machine [47] is incorporated in the proposed algorithm.

(141) $$\left(x_{i},E_{i}\right)\;;\;x_{i}={\left[x_{i1},x_{i2},..,x_{idn}\right]}^{E}\in {SU}^{dn},E_{i}={\left[E_{i1},E_{i2},..,E_{idn}\right]}^{E}\in {SU}^{dn}$$

(142) $$\sum_{i=1}^{N}\beta_{i}\cdot kn\left(\omega_{i}x_{j}+a_{i}\right)=E_{j},j=1,2,3,..,N$$

(143) Outputmatrix(O)·Outputweight(β)=E

(144) $$O\left(x_{1},..x_{L};\omega_{1},..,\omega_{L};a_{1},..,a_{l}\right)=\left[\begin{array}{ccc} kn\left(\omega_{1}x_{1}+a_{1}\right) & \cdots & kn\left(\omega_{L}x_{1}+a_{L}\right)\\ \vdots & \ddots & \vdots \\ kn\left(\omega_{1}x_{N}+a_{1}\right) & \cdots & kn\left(\omega_{L}x_{N}+a_{L}\right) \end{array}\right]$$

(145) $$\beta =O^{-1}\cdot E$$

In the Exploration Division North Sumatra Island Pongo abelii will consider the location to chase for succeeding head-to-head prey site. From side to side the support of this transformation, North Sumatra Island Pongo abelii will be in least number of iterations and definitely be fixed in optima solutions in the multi-layered area. These will support to reduce the computing epoch and once watching for the succeeding outstanding exploration agent situation.

**Fig. 8:**
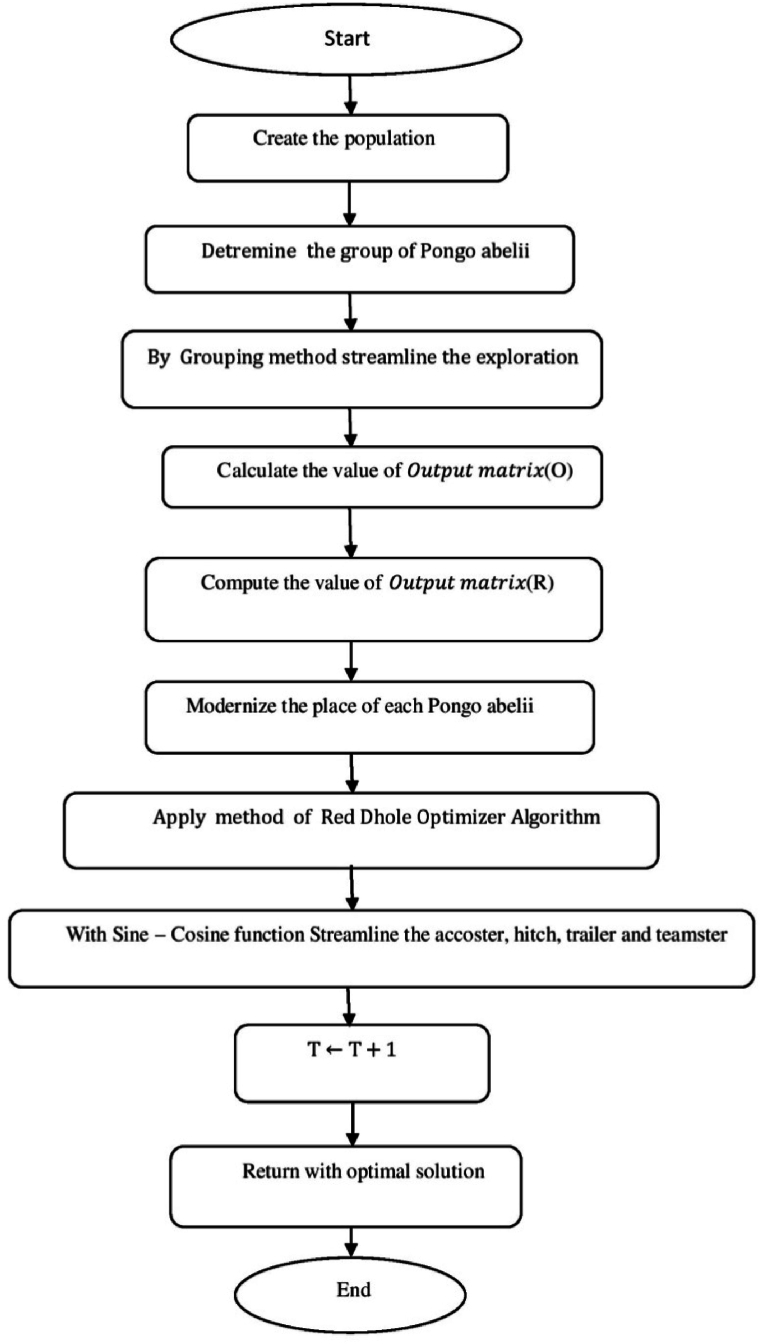
Flow chart of SARANI algorithm.

Fig. 8 shows the flow chart of SARANI algorithm.

a. Start
b. SetN:
c. T←1:
d. Create the population
e. whileT<maxiterdo:
f. ForeachPongoabeliido:
g. DetreminethegroupofPongoabelii:
h. ByGroupingmethodstreamlinetheexploration:
i. End for
j. Foreachexplorationdo:
k. End for
l. Foreveryexplorationdo:
m. if∅<0.5then:
n. ifT<1then:
o. With alignment to the “training set” of data - calculate the value of Outputmatrix(O):
p. $U\left(x_{1},..x_{L};\omega_{1},..,\omega_{L};a_{1},..,a_{l}\right)=\left[\begin{array}{ccc} k\left(\omega_{1}x_{1}+a_{1}\right) & \cdots & k\left(\omega_{L}x_{1}+a_{L}\right)\\ \vdots & \ddots & \vdots \\ k\left(\omega_{1}x_{N}+a_{1}\right) & \cdots & k\left(\omega_{L}x_{N}+a_{L}\right) \end{array}\right]$:
q. Determine the rate of weight (output)
r. $\beta =u^{-1}\cdot E$:
s. With alignment to the “test set” of data - estimate the value of Outputmatrix(R):
t. $U\left(x_{1},..x_{L};\omega_{1},..,\omega_{L};a_{1},..,a_{l}\right)=\left[\begin{array}{ccc} k\left(\omega_{1}x_{1}+a_{1}\right) & \cdots & k\left(\omega_{L}x_{1}+a_{L}\right)\\ \vdots & \ddots & \vdots \\ k\left(\omega_{1}x_{N}+a_{1}\right) & \cdots & k\left(\omega_{L}x_{N}+a_{L}\right) \end{array}\right]$:
u. Estimate the actual value by βandR:
v. Modernize the place of each Pongo abelii
w. $b_{P}\left(t_{i}+1\right)=b_{p}-b.u$:
x. OrElseifT>1then:
y. Erratically prefer the exploration
z. End if
aa. Otherwiseif∅>0.5then:
bb. Renovate the place of each Pongo abelii
cc. $b_{P}\left(t_{i}+1\right)=\left\{ \begin{array}{c} b_{p}\left(t_{i}\right)-x.d,\text{if}\emptyset <0.5\\ \;\text{Ch},\text{if}\;\text{if}\emptyset >0.5 \end{array}\right.$:
dd. End if
ee. End for
ff. ApplymethodofRedDholeOptimizerAlgorithm:
gg. Parameters values are verified
hh. Streamline the accoster, hitch, trailer and teamster
ii. T←T+1:
jj. End while
kk. Return with optimal solution
ll. End

Empress SARANI Optimization Algorithm is designed by integrating Parastylotermes Empress inspired optimization (PEIO) algorithm, Dryocopus martius optimization (DMO) algorithm, Ostrya Carpinifolia Search Optimization (OCSO) Algorithm with SARANI algorithm.

PEIO algorithm is designed based on the deeds of Parastylotermes is an extensively scattered totally societal creature, disseminated transversely in terrain's zone, rigorous in stifling and subtropical provinces. Parastylotermes owns a partition of workforce counting an Empress, employees, and combatants. The Empress puts offspring in the bubble all her lifecycle; employees discover nutrition, oblige the Empress, and renovation the bubble; and combatants with gigantic chins preserve the cluster in safe mode. Aimed at the persistence of the cluster, Parastylotermes inhabitants display the actions of nuptial flying. Beginning April over June every year, a huge quantity of Parastylotermes nurture their wings beneath the biological guidelines of the Empress, hovering out of their bubble in pursuit of a fresh one. This performance enlarges Parastylotermes terrain and evades inbreeding, and it can yield feeble entities. Enthused by the partition of workers and nuptial flying of Parastylotermes, a new algorithm has been developed.

Through Confined planning standard and engendering, zone will be alienated rendering to the comparative location in every facet. The median of the facet is defined as,

(146) $${Median}_{j}={Upper\;bound}_{j}+{Lower\;bound}_{j}/2$$

Then the confined directory for every facet is computed by,

(147) $$C_{j}=\left\{ \begin{array}{c} 1,{Median}_{j}\leq z_{j}\leq {Upper\;bound}_{j}\\ -1,{Lower\;bound}_{j}\leq z_{j}\leq {Median}_{j} \end{array}\right.$$

$$where\;C_{j}\;specify\;the\;confined\;location$$

$$z_{j},C_{j}\;signify\;the\;coordinate\;and\;confined\;directory$$

Upper and lower limits of confined planning engendering are premeditated grounded on the analogous confined directory. For $C_{j}=1\text{,}$ the upper limit of coordinates in *j*th facet is the higher limit of the entire zone, and lower limit is the median. Parastylotermes Empress inspired optimization (PEIO) algorithm every candidate solution is presumed to be a Parastylotermes, whose populace is articulated mathematically as follows,

(148) $$Z={\left[\begin{array}{ccc} Z_{1,1} & \cdots & Z_{1,M}\\ \vdots & \ddots & \vdots \\ Z_{N,1} & \cdots & Z_{N,M} \end{array}\right]}_{N\times M}$$

whereMisthemagnitudeorfacet

NisthenumberofParastylotermes

The limits of the region defined as,

(149) $$Limits\;of\;the\;region=\left\{ \begin{array}{c} \vec{UB}=\left[{UB}_{1},{UB}_{2},..,{UB}_{M}\right]\\ \vec{LB}=\left[{LB}_{1},{LB}_{2},..,{LB}_{M}\right] \end{array}\right.$$

whereUB,LBareupperandlowerboundlimits

Subsequent to Engendering, Z is appraised, and Parastylotermes with the optimum fitness rate is castoff as the present optimum solution. PEIO algorithm is stimulated by the separation of workers in Parastylotermes populations, which distinguishes entities by individuality and modernizes their positions in dissimilar methods. PEIO algorithm owns the individualities as: Empress, hovering employee, scavenging employee, aiding employee, and combatant. The finest solution signifies the Empress, and additional candidate elucidations are distinguished by the outstanding individualities in iterations. The separation encounters the guarantee of nutrition spring, N/2 scavenging employees are continued in iterations and outstanding Parastylotermes are alienated into aiding employees and combatants in 4.0:1.0 proportion. If nuptial flying happens, all entities other than scavenging employees turn out to be hovering employees. Rendering to Scavenging employees if Fitness $\left(F_{i}\right)$ of the *i*th Parastylotermes is a lesser amount of than the moderate fitness $\left(F_{i}\right)$, the Parastylotermesis distinguished as a scavenging employee. Scavenging performance is a backward and forward drive, either far away from or close to the Empress. The position of scavenging employees are rationalized as follows,

(150) $${\vec{Z}}_{i}=\vec{Aim\;}\times R_{1}+{\vec{Z}}_{i}\times \left(1-R_{1}\right)$$

$$where\vec{Aim\;}\;specify\;the\;target\;of\;the\;present\;drive$$

$$R_{1}\in \left[0,1\right]$$

(151) $$\vec{O.B.Z}=\vec{UB}+\vec{LB}-\vec{B.Z}$$

$$where\;\vec{O.B.Z}\;specify\;the\;opposite\;position\;of\;the\;Empress\;Location$$

$$\vec{B.Z}\;indicate\;the\;Empress\;Location$$

UB,LBareupperandlowerlimits

$$when\;R_{1}>0.50\;then\;the\;modernized\;point\;nearby\;to\;the\;\vec{Aim\;}$$

$$when\;R_{1}<0.50\;then\;the\;modernized\;point\;nearby\;to\;the\;{\vec{Z}}_{i}$$

Rendering to Combatants: Parastylotermes with fitness superior than fitness $\left(F_{i}\right)$, is distinguished as a combatant or aiding employee with corresponding possibilities of 20%and80%. A combatant displays nearby actions centred on the Empress, with its location modernized as,

(152) $${\vec{Z}}_{i}=\left(2.0\cdot {\vec{Z}}_{t}-{\vec{Z}}_{i}\right)*R_{2}+{\vec{Z}}_{i}\times \left(1-R_{2}\right)$$

$$where\;{\vec{Z}}_{t}\;specify\;the\;axis\;of\;equilibrium\;of\;the\;exploration\;zone$$

$$R_{2}\in \left[0,1\right]$$

$$when\;R_{2}>0.50\;then\;the\;modernized\;point\;nearby\;to\;the\;axis\;of\;equilibrium$$

$$when\;R_{2}<0.50\;then\;the\;modernized\;point\;nearby\;to\;the\;{\vec{Z}}_{i}$$

(153) $${\vec{Z}}_{t}=R*\left(\vec{UB}-\vec{LB}\right)+\vec{LB}$$

R∈[0,1]

Rendering to aiding employees; Empress passages her gigantic physique with strain, and aiding employees are accountable for aiding Empress to consume and fresh her physique. The procedure can be perceived as aiding employees oncoming the Empress from dissimilar orders, with locations rationalized as,

(154) $$Z_{i,j}=\left\{ \begin{array}{c} {B.Z}_{j}+e_{1}\left({b.z}_{j}-{B.Z}_{j}\right)e_{2},R_{3}>0.50\\ {B.Z}_{j}-e_{1}\left({b.z}_{j}-{B.Z}_{j}\right)e_{2},R_{3}\leq 0.50 \end{array}\right.$$

$$where\;e_{1}\;and\;e_{2}\;used\;to\;regulate\;the\;Exploitation\;intensity$$

$${b.z}_{j}\;is\;the\;arbitrary\;region$$

$$R_{3}\in \left[0,1\right]$$

$$e_{1}\;and\;e_{2}\;is\;computed\;as\;follows\text{,}$$

(155) $$S\left(z\right)=1/1+e^{z}$$

(156) $$e_{1}={\left(1-\left(S\left(\tan\left(-\frac{\pi }{2}+\pi {\left(\frac{t}{T}\right)}^{7}\right)\right)\right)\right)}^{1/1000}*\left(\cos\left(\frac{\pi t}{T}\right)+1\right)$$

(157) $$e_{2}=R^{2.0-e_{1}}$$

R∈[0,1]

$${where\;R}_{3}\;regulate\;the\;course\;of\;exploration\;actions$$

Rendering to hovering employees; to fall into confined (local) optimum is analogous in certain means to inbreeding in Parastylotermes populaces. Parastylotermes evade this confined (local) optimum by nuptial flying, and PEIO algorithm integrates this exercise. In nuptial flying, a great quantity of Parastylotermes flying employees hovers to a fresh bubble, with their locations streamlined as follows,

(158) $$Z_{i,j}=\left\{ \begin{array}{c} L.Z_{j}+e_{1}\left({l.z}_{j}-{L.Z}_{j}\right)e_{2},R_{4}>0.50\\ L.Z_{j}-e_{1}\left({l.z}_{j}-{L.Z}_{j}\right)e_{2},R_{4}\leq 0.50 \end{array}\right.$$

whereLspecifythelevy[36,37]

$$R_{4}\in \left[0,1\right]$$

(159) V(i+1)=C(i)−|d(t)|×E×L(d)

L(s)∼|s|−1−βwhere0<ß<2

(160) $$L\left(s,\gamma ,\mu \right)=\left\{ \begin{array}{c} \sqrt{\frac{\gamma }{2\pi }}\\ 0\;\text{if}\;s\leq 0\; \end{array}\right.\exp\left[-\frac{\gamma }{2\left(s-\mu \right)}\right]\frac{1}{{\left(s-\mu \right)}^{3/2}}\;\text{if}\;0\;<\mu <s<\infty$$

(161) $$F\left(k\right)=\exp\left[-\alpha {\left|k\right|}^{\beta }\right]\;0<\beta \leq 2\text{,}$$

whereα∈[−1,1]

β∈[0,2]

(162) $$Z^{t+1}=Z^{t}+\alpha ⊕\text{Levy}\;\left(\beta \right)$$

(163) $$Z^{t+1}=Z^{t}+R\;\left(\text{size}\left(D\right)\right)⊕\text{Levy}\left(\beta \right)$$

(164) $$Z^{t+1}=Z^{t}+R\;\left(\text{size}\left(D\right)\right)⊕\text{Levy}\left(\beta \right)∼0.01\frac{u}{{\left|v\right|}^{1/\beta }}\left(Z_{j}^{t}-\text{gb}\right)$$

$$u∼N\left(0,\sigma_{u}^{2}\right)\;v∼N\left(0,\sigma_{v}^{2}\right)$$

(165) σu={Γ(1+β)sin(πβ/2)Γ[(1+β)/2]β2(β−1)/2}1β,σv=1

(166) $$\Gamma \left(Z_{G}^{t}\right)=\left(Z_{G}^{t}-1\right)!$$

Levy flight rationalized as follows,

(167) $$L.Z_{j}=B.Z_{j}$$

a. Start
b. Set the parameters
c. Engender the Parastylotermes population
d. Calculate the fitness value of each Parastylotermes
e. Find $\vec{B.Z}$:
f. $Obtain\;B.\;\text{fitness}\;\left(F_{i}\right)$:
g. while(t<T):
h. For each Parastylotermes: division done based on individualities
i. Modernize the positions of Parastylotermes
j. ${\vec{Z}}_{i}=\vec{Aim\;}\times R_{1}+{\vec{Z}}_{i}\times \left(1-R_{1}\right)$:
k. $\vec{O.B.Z}=\vec{UB}+\vec{LB}-\vec{B.Z}$:
l. $when\;R_{1}>0.50\;then\;the\;modernized\;po\mathrm{int}\;nearby\;to\;the\;\vec{Aim\;}$:
m. $when\;R_{1}<0.50\;then\;the\;modernized\;point\;nearby\;to\;the\;{\vec{Z}}_{i}$:
n. ${\vec{Z}}_{i}=\left(2.0\cdot {\vec{Z}}_{t}-{\vec{Z}}_{i}\right)*R_{2}+{\vec{Z}}_{i}\times \left(1-R_{2}\right)$:
o. $when\;R_{2}>0.50\;then\;the\;modernized\;point\;nearby\;to\;the\;axis\;of\;equilibrium$:
p. $when\;R_{2}<0.50\;then\;the\;modernized\;point\;nearby\;to\;the\;{\vec{Z}}_{i}$:
q. ${\vec{Z}}_{t}=R*\left(\vec{UB}-\vec{LB}\right)+\vec{LB}$:
r. $Z_{i,j}=\left\{ \begin{array}{c} {B.Z}_{j}+e_{1}\left({b.z}_{j}-{B.Z}_{j}\right)e_{2},R_{3}>0.50\\ {B.Z}_{j}-e_{1}\left({b.z}_{j}-{B.Z}_{j}\right)e_{2},R_{3}\leq 0.50 \end{array}\right.$:
s. $S\left(z\right)=1/1+e^{z}$:
t. $e_{1}={\left(1-\left(S\left(\tan\left(-\frac{\pi }{2}+\pi {\left(\frac{t}{T}\right)}^{7}\right)\right)\right)\right)}^{1/1000}*\left(\cos\left(\frac{\pi t}{T}\right)+1\right)$:
u. $e_{2}=R^{2.0-e_{1}}$:
v. $Z_{i,j}=\left\{ \begin{array}{c} L.Z_{j}+e_{1}\left({l.z}_{j}-{L.Z}_{j}\right)e_{2},R_{4}>0.50\\ L.Z_{j}-e_{1}\left({l.z}_{j}-{L.Z}_{j}\right)e_{2},R_{4}\leq 0.50 \end{array}\right.$:
w. Modify the Parastylotermes based on upper and lower limits
x. Streamline $e_{1}$:
y. Calculate the fitness value of each Parastylotermes
z. Update $\vec{B.Z}$:
aa. $Modernize\;B.\;\text{fitness}\;\left(F_{i}\right)$:
bb. t=t+1:
cc. Output the best solution
dd. End

Dryocopus martius optimization (DMO) algorithm is modelled on the association actions between male and female Dryocopus martius. Male Dryocopus martius will fascinate the female through special choral sound. Rendering to the choral sound Female Dryocopus martius will headway in the course of the male Dryocopus martius. Intonation in the choral sound will fluctuate with epoch of interval and this choral sound deliberation persuades the Female Dryocopus martius to headway steadily in the course of the Male Dryocopus martius for association. Numerous Choral sound produced by Male Dryocopus martius will charm the Female Dryocopus martius and this deed is equivalent to information aid in various swarm based approaches. Obviously numerous male Dryocopus martius will engender the choral sound to fascinate the Female Dryocopus martius for association. Principally there will be choral sound disparity between Male Dryocopus martius and this consequently adapts the Female Dryocopus martius course of passage in the direction of male Dryocopus martius which based on the variation in choral sound.

Choral sound deliberation is demarcated as,

(168) $$CSD=\frac{Intensity\;of\text{Choral}\;\text{sound}}{Region\;}$$

CSD→Choralsounddeliberation

Increasing intensity of the choral sound is termed as,

(169) $$CSD=\frac{\text{origin}\;\text{of}\;\text{Increasing}\;\text{intensity}\;\text{of}\;\text{the}\;\text{choral}\;\text{sound}}{{4\pi r}^{2}}$$

Grounded on the distance, intensity of the Choral sound is computed as,

(170) $$D=\left\|\left(L_{cs}\right)-\left(L^{a}\right)\right\|$$

D→distance

$$L_{cs}\to choral\;sound\;location$$

$$L^{a}\to Location\;of\;Dryocopus\;martius\;and\;attention\;to\;the\;choral\;sound$$

Rendering to Intensity of choral sound, enthrallment will happen between the male and female Dryocopus martius which leads to a close association. Dryocopus martius fitness rate is computed. Female Dryocopus martius will get charmed to most superb male Dryocopus martius and the attractiveness is scrupulous as source comparable to fitness rate. Choral sound spring is very significant, for the reason that trivial distance will enlarge the power of Choral sound which is analogous to Choral sound emanation. Based on the distance of the region where the male Dryocopus martius positioned intensity of the choral sound will be. Male and female Dryocopus martius is measured as population. Primarily male Dryocopus martius will be in gargantuan number and in the length of the initial phase of association the sum of male Dryocopus martius reduces due to association. As soon as iteration upsurges exactly the population reduces and through this exploration and exploitation is balanced. Dryocopus martius exist in Bird sanctuary (Shuka Vana) Mysore, India [50,51].

In the preliminary segment female Dryocopus martius will get charmed rendering to the intensity of Choral sound, conversely at finishing segment it will be charmed in the course of most superb male Dryocopus martius. Female Dryocopus martius first pay attention to solo male Dryocopus martius Choral sound and at the finishing segment, the male associate with the female Dryocopus martius and most superb high intensity of Choral sound. Dryocopus martius altered on the foundation of objective function. In the exploration region male Dryocopus martius are the supreme location recognized and Female Dryocopus martius are the foremost examining agent. Position of the female Dryocopus martius is completely grounded on the male Dryocopus martius. As soon as an improved candidate solution accomplished, consequently there will be transformation of the male Dryocopus martius. Dryocopus martius capriciously prompts and every Dryocopus martius is acting as candidate solution. Population and fitness rate of Dryocopus martius is evaluated. Supreme male Dryocopus martius is measured as S-population and it will be primarily outstanding male Dryocopus martius, increasingly female Dryocopus martius swing near to specific supreme male Dryocopus martius.

Progress of the Dryocopus martius is modernized by,

(171) $$L_{i}^{t+1}=L_{i}^{t}+Rand*\frac{\vartheta_{i}^{t}*\left(*\left(L_{S\_pop}^{t}-L_{i}^{t}\right)+\alpha_{malej}*\left(L_{malej}^{t}-L_{i}^{t}\right)\right)}{2}$$

$$L_{i}^{t}\to \;\text{Previous}\;\text{position}\;\text{of}\;\text{Dryocopus}\;\text{martius}$$

$$L_{S-pop}^{t}\to \;\text{supreme}\;\text{location}\;\text{of}\;\text{Dryocopus}\;\text{martius}$$

$$z_{malej}^{t}\;\to \text{male}\;\text{location}\;\text{of}\;\text{Dryocopus}\;\text{martius}$$

$$\vartheta_{i}^{t}\;\to \text{coefficient}\;\text{of}\;\text{Dryocopus}\;\text{martius}\;\text{in}\;\text{th}\;\text{iteration}$$

Random∈[0,1]

$$\vartheta_{i}^{t}=Random\times G$$

G={0,0.8}

$$\vartheta_{i}^{t}>1\;and\vartheta_{i}^{t}\leq 1\to define\;the\;association\;of$$

maleandfemaleDryocopusmartius

(172) $$\alpha =\frac{1}{1+intensity\;of\;{choral\;sound\;}_{j}^{i}}$$

$$\alpha \to \text{charm}\;\text{level}\;\text{based}\;\text{on}\;intensity\;of\;{choral\;sound\;}_{j}^{i}$$

Tangent sigmoid is engaged in the process

(173) $$S=T\;\left(1-\frac{n}{N}\right)$$

nandNarepresentandmaximum

numberofiterations

Amount of Male Dryocopus martius in iteration is demarcated as.

(174) $$\text{sum}\;\text{of}\;\text{male}\;\text{Dryocopus}\;\text{martius}=\left\{Round\left(\frac{M}{2}*\left(1-\frac{n}{N}\right)\right)+1\right\}$$

M→maximumsumofDryocopusmartius

Supreme Male Dryocopus martius is grounded on S-population,

(175) $$L_{i}^{t+1}=L_{i}^{t}+Random*\vartheta_{i}^{t}*\left(L_{S\_pop}^{t}-L_{i}^{t}\right)+\alpha_{S\_pop}$$

Modification in the track and site is grounded on the intensity of choral sound and furthermore if certain endangerment passes by others, then Dryocopus martius will change from the current location.

(176) $$O_{\alpha }=0.8*\frac{\sum_{b}^{b-1}\alpha_{S\_pop}^{i}}{b-1}$$

$$O_{\alpha }\;\to Onset\;value$$

(177) $$L_{change}^{i}=\min-\left(\max-\min\right)*Random$$

Grounded on Choral sound malleability of the Dryocopus martius discovered and immediately there is raised intensity of Choral sound from the supreme male Dryocopus martius consequently the female Dryocopus martius will headway in the direction of male.

(178) $$Q_{S-pop.H\;}=\delta *\left(1-\frac{n}{N}\right)$$

$$Q_{S-pop.H\;}\to \;headway\;value$$

(179) $$S_{pop.\;H}=\left\{ \begin{array}{c} 1\;if\;D\leq Q_{S-pop.H\;}\\ 0\;Else \end{array}\right.$$

Consequently the position of the female Dryocopus martius is designated as,

(180) $$L_{S_{pop.H}}^{i}=L_{i}^{t}+S_{pop.H}*\left\{\left(L_{S\_pop}^{t}-L_{D}\right)*Random\right\}$$

Random∈[0,1]

a. Begin
b. Engender the Dryocopus martius population
c. Fitness value of Dryocopus martius calculated
d. Compute $O_{\alpha }$:
e. $O_{\alpha }=0.8*\frac{\sum_{b}^{b-1}\alpha_{S\_pop}^{i}}{b-1}$:
f. while(iteration<Maximumnumberofiterations):
g. $\text{Sum}\;\text{of}\;\text{male}=\left\{Round\left(\frac{M}{2}*\left(1-\frac{n}{N}\right)\right)+1\right\}$:
h. Classify the Dryocopus martius
i. $\alpha =\frac{1}{1+intensity\;of\;{choral\;sound\;}_{j}^{i}}$:
j. $\vartheta_{i}^{t}=Random\times G$:
k. $\mu_{i}^{t}=R\times Factor\;vaue$:
l. Update the position of the Dryocopus martius
m. $L_{i}^{t+1}=L_{i}^{t}+Random*\frac{\vartheta_{i}^{t}*\left(*\left(L_{S\_pop}^{t}-L_{i}^{t}\right)+\alpha_{malej}*\left(L_{malej}^{t}-L_{i}^{t}\right)\right)}{2}$:
n. Compute the Headway of Dryocopus martius
o. $S_{pop.\;H}=\left\{ \begin{array}{c} 1\;if\;D\leq Q_{S-pop.H\;}\\ 0\;Else \end{array}\right.$:
p. $L_{S_{pop.H}}^{i}=L_{i}^{t}+S_{pop.H}*\left\{\left(L_{S\_pop}^{t}-L_{D}\right)*Random\right\}$:
q. End if
r. Change the position of female Dryocopus martius
s. If outstanding solution recognized, then
t. Update S_pop:
u. End for
v. N=N+1:
w. Output the best solution
x. End

Ostrya Carpinifolia Search Optimization (OCSO) Algorithm imitates the Ostrya Carpinifolia growing, seed sprinkling, and root dissemination. Ostrya Carpinifolia grows various regions in the world like Europe, Asia and Caucasus area. In India, in states of Arunachal Pradesh, Assam and SGS Botanical garden, Mysore, India Ostrya Carpinifolia has been found [50,51].

In the growing segment, Ostrya Carpinifolia competes for growing and progresses their fitness rate. In theseed sprinkling segment exploration is attained through sprinkling of Ostrya Carpinifolia seeds around the region. In the root dissemination segment, exploitation is attained through the dissemination of Ostrya Carpinifolia roots around the region.

The population of OCSO algorithm is initialized as follows,

(181) $$O_{i}=\left[o_{i1},o_{i2},o_{i3},\ldots ,o_{iD}\right]$$

$$Each\;entity\;o_{ij}\in O_{i}\;is\;engendered\;as\;follows\text{,}$$

(182) $$o_{ij}=UD*\left(\max_{j}-\min_{j}\right)+\min_{j}$$

UD→uniformlydistributednumber[0,1]

$$\max_{j}\;and\;\min_{j}\to limits$$

Each Ostrya Carpinifolia has a fitness ratewhich has been found by assessing the fitness value. The fitness of each Ostrya Carpinifolia is associated with objective function. Ostrya Carpinifolia which located in upright zones raises well and attains advanced fitness rate.

Growing conditions of Ostrya Carpinifolia differs, rendering to the region, since few places competition between the Ostrya Carpinifolia prevails because all the Ostrya Carpinifolia has to utilize the available resources in that region and in few places there won't be any competition. The growing conditions of Ostrya Carpinifolia is defined as,

(183) $$O_{i}^{t+1}=O_{i}^{t}+\left(\beta .gwc+\left(1-\beta \right)\frac{1}{1+\tau }gwc\right)$$

$$O_{i}^{t}\to location\;of\;Ostrya\;Carpinifolia$$

β=1

gwc−growingconditions

τ−conditionofcompetition

whenβ=0,thengrowingratedecreaseswithτ

(184) $$gwc=\left(1-e^{-{\left(\frac{t}{T}\right)}^{2}}\right)*Z$$

t,T→currentandmaximumiterations

(185) Z=B(−α,α)

α∈[0,1]

The condition of competition is defined as,

(186) $$\tau =\sum_{j=1}^{A_{i}}\left(\frac{f_{i}^{t}}{f_{j}^{t}}\right)*\arctan\left(\frac{f_{j}^{t}}{d_{ij}^{t}}\right)$$

$$A_{i}\to neighbouring\;Ostrya\;Carpinifolia$$

f→fitnessrate

d→distanceamongOstryaCarpinifolia

(187) $$d_{ij}^{t}=\left\|O_{j}^{t}-O_{i}^{t}\right\|$$

(188) $$A_{i}=\lceil \sigma_{i}*N\rceil$$

N→sizeofthepopulation

$$\sigma_{i}\to normalized\;fitness\;rate\;of\;O_{i}$$

(189) $$\sigma_{i}=\frac{m\left(B\right)-f_{i}}{\sum_{k=1}^{N}\left(m\left(B\right)-f_{i}\right)}$$

m→maximum

$$B=\left\{f_{k}|k=1,\ldots ,N\right\}$$

The neighbouring Ostrya Carpinifolia population is defined as,

(190) $$C_{i}=\left[O_{1},O_{2},\ldots ,O_{A_{i}}\right]$$

Ostrya Carpinifolia growing conditions will differ zone to zone rendering to competition factors.

Then the seed sprinkling segment is defined as,

(191) $$Q^{t}=\lfloor e^{t}*N\rfloor$$

$$e^{t}\to \;Ostrya\;Carpinifolia\;seed\;sprinkling\;rate$$

Aggregating the seed sprinklingrate upsurges the procedure's autonomy to explore external to the present area of solution region; nevertheless, it upsurges the computational complication.

(192) $$Q_{i}=O_{i}^{t}+L\left(H_{i}^{t}-H_{best}^{t}\right)$$

L→levy

(193) L(s)∼|s|−1−βwhere0<ß<2

(194) $$L\left(s,\gamma ,\mu \right)=\left\{ \begin{array}{c} \sqrt{\frac{\gamma }{2\pi }}\\ 0\;\text{if}\;s\leq 0\; \end{array}\right.\exp\left[-\frac{\gamma }{2\left(s-\mu \right)}\right]\frac{1}{{\left(s-\mu \right)}^{3/2}}\;\text{if}\;0\;<\mu <s<\infty$$

(195) $$F\left(k\right)=\exp\left[-\alpha {\left|k\right|}^{\beta }\right]\;0<\beta \leq 2\text{,}$$

(196) $$L\left(\beta \right)∼0.01\frac{u}{{\left|v\right|}^{1/\beta }}\left(Q_{j}^{t}-c\right)$$

$$u∼N\left(0,\sigma_{u}^{2}\right)\;v∼N\left(0,\sigma_{v}^{2}\right)$$

(197) σu={Γ(1+β)sin(πβ/2)Γ[(1+β)/2]β2(β−1)/2}1β,σv=1

(198) $$O_{i}^{t+1}=\left\{ \begin{array}{c} Q_{i}\;if\;f\left(Q_{i}\right)>f\left(O_{i}^{t}\right)\\ O_{i}^{t}\;Otherwise\; \end{array}\right.$$

Root dissemination factor is projected to upsurge the exploitation capability and convergence speed of the procedure by carrying out the local search. It is significant that, local search is executed only round the present fittest Ostrya Carpinifolia.

(199) $$H_{j}=\left\{ \begin{array}{c} o_{best,j}^{t}+{radius}^{t}\left(\max_{j}-\min_{j}\right)\left(v^{t}-0.5\right),j==k\\ o_{best,j}^{t}\;j\neq k \end{array}\right.$$

$$H_{j}\to Root\;of\;Ostrya\;Carpinifolia\;location$$

$$v^{t}\to chaotic\;variable$$

$$\max_{j}\;and\;\min_{j}\to limits$$

(200) $${radius}^{t}=\frac{1}{e^{\frac{t}{T}}}$$

In every generation Root dissemination factor engenders new solutions as follows,

(201) $$H_{best}=m\left\{H_{1},H_{2},\ldots ,H_{D}\right\}$$

When $H_{best}$ is better than present best ${\;O}_{best}$, then swapping occurs and it will be taken as best solution for the subsequent generation.

(202) $$O_{best}^{t+1}=\left\{ \begin{array}{c} H_{best}\;if\;f\left({\;H}_{best}\right)>f\left(O_{best}^{t}\right)\\ O_{best}^{t}\;otherwise \end{array}\right.$$

a. Start
b. Set the parameters
c. Engender the preliminary population
d. Compute the fitness value
e. $Obtain\;the\;O_{best}in\;the\;population$:
f. while(t≤T)do:
g. Apply the growing segment
h. Fori=1toNdo:
i. Calculate the growing rate
j. $gwc=\left(1-e^{-{\left(\frac{t}{T}\right)}^{2}}\right)*Z$:
k. Binaryrandomnumberengenderedbyβ:
l. if(β==0)then:
m. Recognize the neighbour
n. $A_{i}=\lceil \sigma_{i}*N\rceil$:
o. $\sigma_{i}=\frac{m\left(B\right)-f_{i}}{\sum_{k=1}^{N}\left(m\left(B\right)-f_{i}\right)}$:
p. $C_{i}=\left[O_{1},O_{2},\ldots ,O_{A_{i}}\right]$:
q. Calculatetheconditionofcompetition:
r. $\tau =\sum_{j=1}^{A_{i}}\left(\frac{f_{i}^{t}}{f_{j}^{t}}\right)*\arctan\left(\frac{f_{j}^{t}}{d_{ij}^{t}}\right)$:
s. Computethegrowth:
t. $O_{i}^{t+1}=O_{i}^{t}+\left(\beta .gwc+\left(1-\beta \right)\frac{1}{1+\tau }gwc\right)$:
u. Compute the fitness value
v. Apply seed sprinkling segment
w. Rendering to fitness classification done in the population
x. Calculate the seed sprinkling rate
y. $Q^{t}=\lfloor e^{t}*N\rfloor$:
z. $For\;i=1\;to\;Q^{t}\;do$:
aa. $Q_{i}=O_{i}^{t}+L\left(H_{i}^{t}-H_{best}^{t}\right)$:
bb. Computethefitnessvalue:
cc. $O_{i}^{t+1}=\left\{ \begin{array}{c} Q_{i}\;if\;f\left(Q_{i}\right)>f\left(O_{i}^{t}\right)\\ O_{i}^{t}\;Otherwise\; \end{array}\right.$:
dd. Apply root dissemination segment
ee. Obtain the present best solution
ff. Fork=1toDdo:
gg. Calculate the location of root
hh. $H_{j}=\left\{ \begin{array}{c} o_{best,j}^{t}+{radius}^{t}\left(\max_{j}-\min_{j}\right)\left(v^{t}-0.5\right),j==k\\ o_{best,j}^{t}\;j\neq k \end{array}\right.$:
ii. Compute the fitness value
jj. Modernize the radius value
kk. ${radius}^{t}=\frac{1}{e^{\frac{t}{T}}}$:
ll. $H_{best}=m\left\{H_{1},H_{2},\ldots ,H_{D}\right\}$:
mm. Comparethefitnessvalues:
nn. $O_{best}^{t+1}=\left\{ \begin{array}{c} H_{best}\;if\;f\left({\;H}_{best}\right)>f\left(O_{best}^{t}\right)\\ O_{best}^{t}\;otherwise \end{array}\right.$:
oo. t=t+1:
pp. Output the best solution
qq .End

**Fig. 9:**
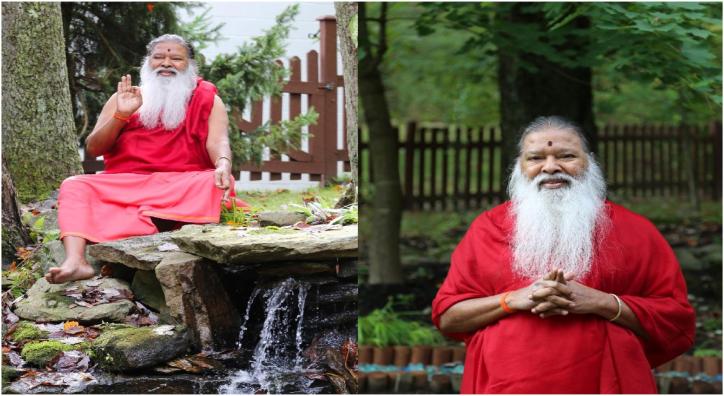
Hermit in hermitage [50,51].

Hermitage Activities Inspired optimization (HAIO) algorithm imitates the activities of a hermitage, where a Hermit reside for religious reasons and through the miraculous powers erase the difficulty of all beings. Many Hermits establish Hermitage where the followers and dedicated service oriented people will dwell in certain distance of the same hermitage compound. Around the hermit dwelling place after some distance - few establishments are erected for staying of the followers and dedicated service oriented people. The followers will perform the basic works, including obtaining the necessary things to maintain the day to day activities and spreading the branches of the Hermitage. Then certain generative followers will be there in the hermitage and they will act in time when the followers unable to perform the duties. Sequentially protecting followers will be there in order to protect the hermitage. Hermit [50] is staying in the hermitage (Fig. 9).

Followers, generative followers and protecting follower's actions are imitated to balance the exploration and exploitation in the procedure. Preliminary population steadily registers the data obtained at iterations and communicates it to followers and protecting followers in subsequent iteration. This process is recurrent up until the comprehensive optimum is established. Lévy flight is applied to enhance the solution. The actions of a Hermitage is mathematically defined as,

(203) $$Z\left(n\right)=\sum_{i=1}^{n}M_{i}$$

(204) $$M_{i}=M_{1},M_{2}+\ldots +M_{n}$$

$$M_{i}\to actions\;of\;a\;Hermitage$$

Actions of a Hermitage is defined as,

(205) $$M=\frac{U}{{\left|V\right|}^{\frac{1}{\beta }}}$$

β→Levydistribution;1≤β≤2

$$u∼N\left(0,\sigma_{u}^{2}\right),v∼N\left(0,\sigma_{v}^{2}\right)$$

(206) σu={Γ(1+β)sin(πβ/2)Γ[(1+β)/2]β2(β−1)/2}1β,σv=1

Preliminary population of the Hermitage will be in the N size and the quantity of the followers in Hermitage is,

$$Z_{i,followers},1\leq i\leq 0.70N$$

1≤i≤0.70N→positionoffollowers

The important duty of the followers in the Hermitage is to perform the basic works and expand the branches of the hermitage in multiple directions. The position update is defined as,

(207) $$Z_{i,followers}^{k+1}=Z_{i,followers}^{k}+\emptyset_{1}\left(dZ_{i,followers}^{k+1}\right)$$

(208) $$\emptyset_{1}=-1+2Random\left(1\right)$$

$$\emptyset_{1}=\left[-1,1\right]$$

$$dZ_{i,followers}^{k+1}\to direction\;of\;movement\;and\;actions\;of\;followers$$

$$\emptyset_{1}<0,\emptyset_{1}>0\;;Movement\;directions\;of\;followers$$

Hermitage followers will passage in multiple directions for obtaining the basic needs and expanding the hermitage,

(209) $$dZ_{i,followers}^{k+1}=dM\left(k+1\right)⊗\left|Z_{best}^{k}-Z_{i,followers}^{k}\right|$$

(210) dM(k+1)=(Random(1,Dim)+M(k+1))

dM(k+1)→movementchange

Dim→Dimension

Random(1,Dim)=[0,1]

The position update of each Hermitage follower is defined as,

(211) $$Z_{i,follower}^{k+1}=Z_{i,follower}^{k}+\left(-1+2r\left(1\right)\right)\left[r\left(1,\mathrm{Dim}\right)+M\left(k+1\right)⊗\left|Z_{best}^{k}-Z_{i,followers}^{k}\right|\right]$$

r→random

dM(k+1)→willcreatewideandnarrowsearchspace

In the last segment of iterations dM(k+1) will engender the narrow search space and from the start it will create the wide search space, then convergence rate will be in better mode.

β→increasefrom1.5to2withreferencetoiterations

**Fig. 10:**
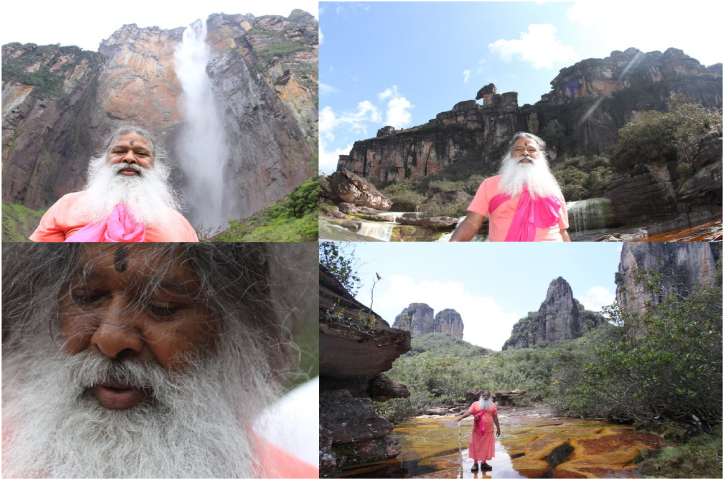
Hermit in angel falls, Venezuela [52].

**Fig. 11:**
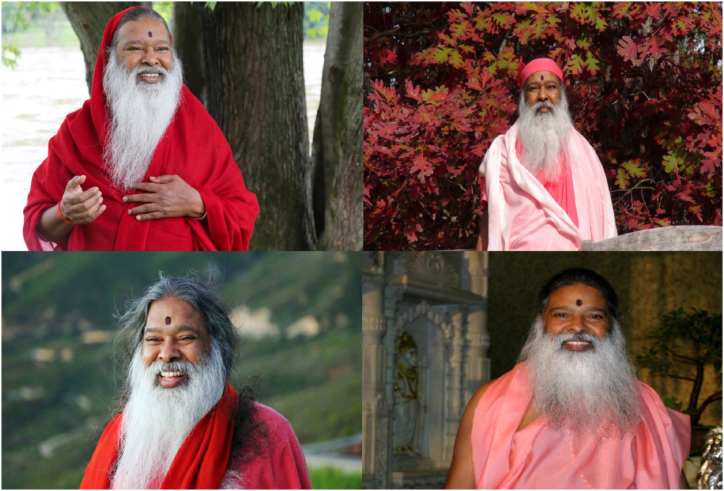
Hermit at various locations around the world [50,51].

The generative followers in Hermitage will construct new hermitage, when followers in the Hermitage unable to expand the branches through their actions. Fig. 10 shows the Hermit movement in Angel falls, Venezuela [45] and Fig. 11 show the hermit position in various locations around the world [50,51].

τ−theparameterwilldefinetheactionsofgenerativefollowers

ifT≥τ,then

Position identified through new hermitage by generative followers is defined as,

(212) $$Z_{i,g-\text{followers}}^{k+1}=Z_{best}+M\left(k+1\right)$$

g−followers→generativefollowers

Quantity of the protecting follower's in Hermitage is defined as,

$$Z_{i,p-followers},0.70N\leq i\leq N$$

p−followers→protectingfollower’s

The main goal of the protecting follower's in Hermitage is to protect the hermitage. The location of the protecting follower's in hermitage is defined as,

(213) $$Z_{i,p-followers}^{k+1}=Z_{best}+dZ_{best}$$

$$dZ_{best}\to \;Movement\;directions\;of\;p-followers$$

(214) $$dZ_{best}=\emptyset_{2}\left|Z_{best}⊗M\left(k+1\right)-Z_{i,p-followers}^{k}\right|$$

(215) $$\emptyset_{2}=-1+2Random\left(1\right)$$

$$\emptyset_{2}\to direction\;of\;safeguarding\;by\;p-followers$$

(216) $$dZ_{best}=-1+2Random\left(1\right)\left|Z_{best}⊗M\left(k+1\right)-Z_{i,p-followers}^{k}\right|$$

a. Start
b. Set the parameters
c. Engender the Hermitage population
d. Compute the objective functional value for each Hermitage
e. Modernize the best solution
f. Engender the τvalue:
g. $For\;k=1;K_{maximum}$:
h. Computethevalueofβ:
i. Compute M value
j. $M=\frac{U}{{\left|V\right|}^{\frac{1}{\beta }}}$:
k. $\text{For}\;\text{each}\;\text{Hermitage}\;\text{workforce}\;Z_{i}\left(1\leq i\leq 0.70N\right)$:
l. Update the location of Hermitage followers
m. $Z_{i,followers}^{k+1}=Z_{i,followers}^{k}+\emptyset_{1}\left(dZ_{i,followers}^{k+1}\right)$:
n. ComputetheobjectivefunctionalvalueforeachHermitagefollowers:
o. Modernize the best solution
p. Else
q. T(i)=T(i)+1:
r. Endif:
s. ifT≥τ,then:
t. Generativefollowerswillexplorenewpossibleplaces:
u. $Z_{i,g-\text{followers}}^{k+1}=Z_{best}+M\left(k+1\right)$:
v. Compute the objective functional value for each Generative follower Hermitage
w. Modernize the best solution
x. T(i)=0:
y. End if
z. End for
aa. $\text{For}\;\text{each}\;\text{Hermitage}\;\text{protecting}\;\text{follower}’sZ_{i}\left(0.70N\leq i\leq N\right)$:
bb. Update the position of Hermitage protecting follower's
cc. $Z_{i,p-\text{follower}’s\;}^{k+1}=Z_{best}+dZ_{best}$:
dd. Compute the objective functional value for each Hermitage protecting follower
ee. Modernize the best solution
ff. End for
gg. t=t+1:
hh. Output the best solution
ii. End

**Fig. 12:**
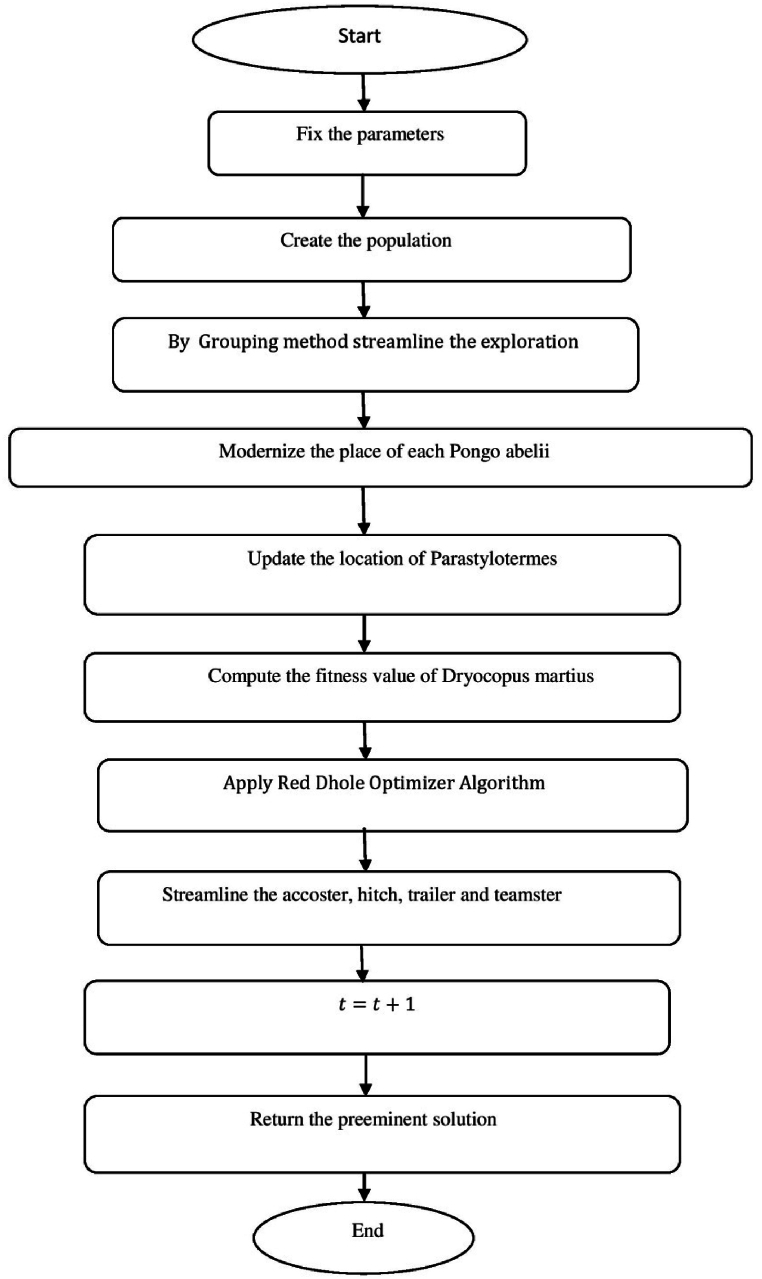
Flow chart of Empress SARANI Optimization Algorithm.

Empress SARANI Optimization Algorithm is designed by integrating Parastylotermes Empress inspired optimization (PEIO) algorithm, Dryocopus martius optimization (DMO) algorithm, Ostrya Carpinifolia Search Optimization (OCSO) Algorithm, Hermitage Activities Inspired optimization (HAIO) algorithm with SARANI algorithm. PEIO algorithm is stimulated by the separation of workers in Parastylotermes populations, which distinguishes entities by individuality and modernizes their positions in dissimilar methods. PEIO algorithm owns the individualities as: Empress, hovering employee, scavenging employee, aiding employee, and combatant. The finest solution signifies the Empress, and additional candidate elucidations are distinguished by the outstanding individualities in iterations.In DMO algorithm, preliminary segment female Dryocopus martius will get charmed rendering to the intensity of Choral sound, conversely at finishing segment it will be charmed in the course of most superb male Dryocopus martius. Female Dryocopus martius first pay attention to solo male Dryocopus martius Choral sound and at the finishing segment, the male associate with the female Dryocopus martius and most superb high intensity of Choral sound. Initially male Dryocopus martius will be in gargantuan number and in the length of the initial phase of association the sum of male Dryocopus martius reduces due to association. As soon as iteration upsurges exactly the population reduces and through this exploration and exploitation is balanced.In OCSO growing segment, Ostrya Carpinifolia competes for growing and progresses their fitness rate. In theseed sprinkling segment exploration is attained through sprinkling of Ostrya Carpinifolia seeds around the region. In the root dissemination segment, exploitation is attained through the dissemination of Ostrya Carpinifolia roots around the region. In HAIO Followers, generative followers and protecting follower's actions are imitated to balance the exploration and exploitation in the procedure. Preliminary population steadily registers the data obtained at iterations and communicates it to followers and protecting followers in subsequent iteration. This process is recurrent up until the comprehensive optimum is established. Fig. 12 shows the flow chart of Empress SARANI Optimization Algorithm.

Empress SARANI Optimization Algorithm.

a. Start
b. SetN:
c. T←1:
d. Create the population
e. whileT<maxiterdo:
f. ForeachPongoabeliido:
g. DetreminethegroupofPongoabelii:
h. ByGroupingmethodstreamlinetheexploration:
i. End for
j. Foreachexplorationdo:
k. End for
l. Foreveryexplorationdo:
m. if∅<0.5then:
n. ifT<1then:
o. $U\left(x_{1},..x_{L};\omega_{1},..,\omega_{L};a_{1},..,a_{l}\right)=\left[\begin{array}{ccc} k\left(\omega_{1}x_{1}+a_{1}\right) & \cdots & k\left(\omega_{L}x_{1}+a_{L}\right)\\ \vdots & \ddots & \vdots \\ k\left(\omega_{1}x_{N}+a_{1}\right) & \cdots & k\left(\omega_{L}x_{N}+a_{L}\right) \end{array}\right]$:
p. $\beta =u^{-1}\cdot E$:
q. $U\left(x_{1},..x_{L};\omega_{1},..,\omega_{L};a_{1},..,a_{l}\right)=\left[\begin{array}{ccc} k\left(\omega_{1}x_{1}+a_{1}\right) & \cdots & k\left(\omega_{L}x_{1}+a_{L}\right)\\ \vdots & \ddots & \vdots \\ k\left(\omega_{1}x_{N}+a_{1}\right) & \cdots & k\left(\omega_{L}x_{N}+a_{L}\right) \end{array}\right]$:
r. Estimate the actual value by βandR:
s. Modernize the place of each Pongo abelii
t. $b_{P}\left(t_{i}+1\right)=b_{p}-b.u$:
u. OrElseifT>1then:
v. Erratically prefer the exploration
w. For each Parastylotermes: division done based on individualities
x. Modernize the positions of Parastylotermes
y. ${\vec{Z}}_{i}=\vec{Aim\;}\times R_{1}+{\vec{Z}}_{i}\times \left(1-R_{1}\right)$:
z. $\vec{O.B.Z}=\vec{UB}+\vec{LB}-\vec{B.Z}$:
aa. $when\;R_{1}>0.50\;then\;the\;modernized\;point\;nearby\;to\;the\;\vec{Aim\;}$:
bb. $when\;R_{1}<0.50\;then\;the\;modernized\;point\;nearby\;to\;the\;{\vec{Z}}_{i}$:
cc. ${\vec{Z}}_{i}=\left(2.0\cdot {\vec{Z}}_{t}-{\vec{Z}}_{i}\right)*R_{2}+{\vec{Z}}_{i}\times \left(1-R_{2}\right)$:
dd. $when\;R_{2}>0.50\;then\;the\;modernized\;point\;nearby\;to\;the\;axis\;of\;equilibrium$:
ee. $when\;R_{2}<0.50\;then\;the\;modernized\;point\;nearby\;to\;the\;{\vec{Z}}_{i}$:
ff. ${\vec{Z}}_{t}=R*\left(\vec{UB}-\vec{LB}\right)+\vec{LB}$:
gg. $Z_{i,j}=\left\{ \begin{array}{c} {B.Z}_{j}+e_{1}\left({b.z}_{j}-{B.Z}_{j}\right)e_{2},R_{3}>0.50\\ {B.Z}_{j}-e_{1}\left({b.z}_{j}-{B.Z}_{j}\right)e_{2},R_{3}\leq 0.50 \end{array}\right.$:
hh. $S\left(z\right)=1/1+e^{z}$:
ii. $e_{1}={\left(1-\left(S\left(\tan\left(-\frac{\pi }{2}+\pi {\left(\frac{t}{T}\right)}^{7}\right)\right)\right)\right)}^{1/1000}*\left(\cos\left(\frac{\pi t}{T}\right)+1\right)$:
jj. $e_{2}=R^{2.0-e_{1}}$:
kk. $Z_{i,j}=\left\{ \begin{array}{c} L.Z_{j}+e_{1}\left({l.z}_{j}-{L.Z}_{j}\right)e_{2},R_{4}>0.50\\ L.Z_{j}-e_{1}\left({l.z}_{j}-{L.Z}_{j}\right)e_{2},R_{4}\leq 0.50 \end{array}\right.$:
ll. Modify the Parastylotermes based on upper and lower limits
mm. Engender the Dryocopus martius population
nn. Compute the fitness value of Dryocopus martius
oo. Compute $O_{\alpha }$:
pp. $O_{\alpha }=0.8*\frac{\sum_{b}^{b-1}\alpha_{S\_pop}^{i}}{b-1}$:
qq. while(iteration<Maximumnumberofiterations):
rr. $\text{Sum}\;\text{of}\;\text{male}=\left\{Round\left(\frac{M}{2}*\left(1-\frac{n}{N}\right)\right)+1\right\}$:
ss. Classify the Dryocopus martius
tt. $\alpha =\frac{1}{1+intensity\;of\;{choral\;sound\;}_{j}^{i}}$:
uu. $\vartheta_{i}^{t}=Random\times G$:
vv. $\mu_{i}^{t}=R\times Factor\;vaue$:
ww. Update the position of the Dryocopus martius
xx. $L_{i}^{t+1}=L_{i}^{t}+Random*\frac{\vartheta_{i}^{t}*\left(*\left(L_{S\_pop}^{t}-L_{i}^{t}\right)+\alpha_{malej}*\left(L_{malej}^{t}-L_{i}^{t}\right)\right)}{2}$:
yy.Compute the Headway of Dryocopus martius
zz $S_{pop.\;H}=\left\{ \begin{array}{c} 1\;if\;D\leq Q_{S-pop.H\;}\\ 0\;Else \end{array}\right.$:
aaa .$L_{S_{pop.H}}^{i}=L_{i}^{t}+S_{pop.H}*\left\{\left(L_{S\_pop}^{t}-L_{D}\right)*Random\right\}$:
bbb. End if
ccc. Otherwiseif∅>0.5then:
ddd. Renovate the place of each Pongo abelii
eee. $b_{P}\left(t_{i}+1\right)=\left\{ \begin{array}{c} b_{p}\left(t_{i}\right)-x.d,\text{if}\emptyset <0.5\\ \;\text{Ch},\text{if}\;\text{if}\emptyset >0.5 \end{array}\right.$:
fff. Apply the growing segment
ggg. Fori=1toNdo:
hhh. Calculate the growing rate
iii. $gwc=\left(1-e^{-{\left(\frac{t}{T}\right)}^{2}}\right)*Z$:
jjj. Binaryrandomnumberengenderedbyβ:
kkk. if(β==0)then:
lll. Recognize the neighbour
mmm. $A_{i}=\lceil \sigma_{i}*N\rceil$:
nnn. $\sigma_{i}=\frac{m\left(B\right)-f_{i}}{\sum_{k=1}^{N}\left(m\left(B\right)-f_{i}\right)}$:
ooo. $C_{i}=\left[O_{1},O_{2},\ldots ,O_{A_{i}}\right]$:
ppp. Calculatetheconditionofcompetition:
qqq. $\tau =\sum_{j=1}^{A_{i}}\left(\frac{f_{i}^{t}}{f_{j}^{t}}\right)*\arctan\left(\frac{f_{j}^{t}}{d_{ij}^{t}}\right)$:
rrr. Computethegrowth:
sss. $O_{i}^{t+1}=O_{i}^{t}+\left(\beta .gwc+\left(1-\beta \right)\frac{1}{1+\tau }gwc\right)$:
ttt. Compute the fitness value
uuu. Apply seed sprinkling segment
vvv. Rendering to fitness classification done in the population
www. Calculate the seed sprinkling rate
xxx. $Q^{t}=\lfloor e^{t}*N\rfloor$:
yyy. $For\;i=1\;to\;Q^{t}\;do$:
zzz. $Q_{i}=O_{i}^{t}+L\left(H_{i}^{t}-H_{best}^{t}\right)$:
aaaa. Computethefitnessvalue:
bbbb. $O_{i}^{t+1}=\left\{ \begin{array}{c} Q_{i}\;if\;f\left(Q_{i}\right)>f\left(O_{i}^{t}\right)\\ O_{i}^{t}\;Otherwise\; \end{array}\right.$:
cccc. Apply root dissemination segment
dddd. Obtain the present best solution
eeee. Fork=1toDdo:
ffff. Calculate the location of root
gggg. $H_{j}=\left\{ \begin{array}{c} o_{best,j}^{t}+{radius}^{t}\left(\max_{j}-\min_{j}\right)\left(v^{t}-0.5\right),j==k\\ o_{best,j}^{t}\;j\neq k \end{array}\right.$:
hhhh. Compute the fitness value
iiii. Modernize the radius value
jjjj. ${radius}^{t}=\frac{1}{e^{\frac{t}{T}}}$:
kkkk. $H_{best}=m\left\{H_{1},H_{2},\ldots ,H_{D}\right\}$:
llll. Comparethefitnessvalues:
mmmm. $O_{best}^{t+1}=\left\{ \begin{array}{c} H_{best}\;if\;f\left({\;H}_{best}\right)>f\left(O_{best}^{t}\right)\\ O_{best}^{t}\;otherwise \end{array}\right.$:
nnnn. Engender the τvalue:
oooo. $For\;k=1;K_{maximum}$:
pppp. Computethevalueofβ:
qqqq. Compute M value
rrrr. $M=\frac{U}{{\left|V\right|}^{\frac{1}{\beta }}}$:
ssss. $\text{For}\;\text{each}\;\text{Hermitage}\;\text{workforce}\;Z_{i}\left(1\leq i\leq 0.70N\right)$:
tttt. Update the location of Hermitage followers
uuuu. $Z_{i,followers}^{k+1}=Z_{i,followers}^{k}+\emptyset_{1}\left(dZ_{i,followers}^{k+1}\right)$:
vvvv. ComputetheobjectivefunctionalvalueforeachHermitagefollowers:
ww ww. Modernize the best solution
xxxx. Else
yyyy. T(i)=T(i)+1:
zzzz. Endif:
aaaaa. ifT≥τ,then:
bbbbb. Generativefollowerswillexplorenewpossibleplaces:
ccccc. $Z_{i,g-\text{followers}}^{k+1}=Z_{best}+M\left(k+1\right)$:
ddddd. Compute the objective functional value for each Generative follower Hermitage
eeeee. Modernize the best solution
fffff. T(i)=0:
ggggg. End if
hhhhh. End for
iiiii. $\text{For}\;\text{each}\;\text{Hermitage}\;\text{protecting}\;\text{follower}’sZ_{i}\left(0.70N\leq i\leq N\right)$:
jjjjj. Update the position of Hermitage protecting follower's
kkkkk. $Z_{i,p-\text{follower}’s\;}^{k+1}=Z_{best}+dZ_{best}$:
lllll. Compute the objective functional value for each Hermitage protecting follower
mmmmm. Modernize the best solution
nnnnn. End if
ooooo. End for
ppppp. ApplymethodofRedDholeOptimizerAlgorithm:
qqqqq. Parameters values are verified
rrrrr. Streamline the accoster, hitch, trailer and teamster
sssss. T←T+1:
ttttt. End while
uuuuu. Return with optimal solution
vvvvv. End

Computation complexity

O(P)=O(O)+O(i)+O(f)+O(s)

O(1)

O(b∗d)

O(U∗f∗n)

O(U∗a∗b∗d)

O(Q)=O(1+b∗d+U∗f∗b+U∗a∗b∗d)

## 11 Simulation results

Projected Pomarine jaeger Optimization (PJO) algorithm, Tiger hunting Optimization (THO) Algorithm, Desert Reynard and Vixen Inspired Optimization (DRVIO) Algorithm, Lonchodidae optimization (LO) algorithm, Caracal optimization (CO) algorithm, Barasingha optimization (BO) algorithm, Amur leopard optimization (AO) algorithm and Empress SARANI Optimization Algorithmare corroborated in 24 benchmark functions [24]. G01 – G024 Functions are utilized.

(217) $$G01\;Function:F\left(x\right)=5\sum_{i=1}^{4}x_{i}-5\sum_{i=1}^{4}x_{i}^{2}-\sum_{i=5}^{13}x_{i}$$

(218) $$G02\;Function:F\left(x\right)=-\left|\frac{\sum_{i=1}^{n}\cos^{4}\left(x_{i}\right)-2{ℿ}_{i=1}^{n}\;\cos^{2}\left(x_{i}\right)}{\sqrt{\sum_{i=1}^{n}ix_{i}^{2}}}\right|$$

(219) $$G03\;Function:F\left(x\right)=-{\left(\sqrt{n}\right)}^{n}{ℿ}_{i=1}^{n}x_{i}$$

(220) $$G04\;Function:F\left(x\right)=5.35x_{3}^{2}+0.83x_{1}x_{5}+37.2x_{1}-40.792.1$$

(221) $$G05\;Function:F\left(x\right)=3x_{1}+0.00000\;x_{1}^{3}+2x_{2}+\left(\frac{0.000002}{3}\right)x_{2}^{3}$$

(222) $$G06\;Function:F\left(x\right)={\left(x_{1}-10\right)}^{3}+{\left(x_{2}-20\right)}^{3}$$

(223) $$G07\;Function:F\left(x\right)=x_{1}^{2}+x_{2}^{2}+x_{1}x_{2}-14x_{1}-16x_{2}+{\left(x_{3}-10\right)}^{2}+4{\left(x_{4}-5\right)}^{2}+{\left(x_{5}-3\right)}^{2}+2{\left(x_{6}-1\right)}^{2}+5\;x_{7}^{2}+7{\left(x_{8}-11\right)}^{2}+{\left(x_{9}-10\right)}^{2}+{\left(x_{10}-7\right)}^{2}+45$$

(224) $$G08\;Function:F\left(x\right)=-\sin^{3}\left(2\pi x_{1}\right)\sin\left(2\pi x_{2}\right)/x_{1}^{3}\left(x_{1}+x_{2}\right)$$

(225) $$G09\;Function:F\left(x\right)={\left(x_{1}-10\right)}^{2}+5{\left(x_{2}-12\right)}^{2}+x_{4}^{3}+3\;{\left(x_{4}-11\right)}^{2}+10x_{5}^{6}+7x_{6}^{2}+x_{7}^{4}-4x_{6}x_{7}-10x_{6}-{8x}_{7}$$

(226) $$G10\;Function:F\left(x\right)=x_{1}+x_{2}+x_{3}$$

(227) $$G11\;Function:F\left(x\right)=x_{1}^{2}+{\left(x_{2}-1\right)}^{2}$$

(228) $$G12\;Function:F\left(x\right)=-100\left(-{\left(x_{1}-5\right)}^{2}-{\left(x_{2}-5\right)}^{2}-{\left(x_{3}-5\right)}^{2}\right)/100$$

(229) $$G13\;Function:F\left(x\right)=e^{x_{1}x_{2}x_{3}x_{4}x_{5}}$$

(230) $$G14\;Function:F\left(x\right)=\sum_{i=1}^{10}x_{i}\left(c_{i}+In\frac{x_{i}}{\sum_{j=1}^{10}x_{j}}\right)$$

(231) $$G15\;Function:F\left(x\right)=1000-x_{1}^{2}-2x_{2}^{2}-x_{3}^{2}-x_{1}x_{2}-x_{1}x_{3}$$

(232) $$G16\;Function:F\left(x\right)=0.0001y_{14}+0.1365+0.000023y_{13}+0.0000015y_{16}+0.03y_{12}+0.0043y_{5}+0.0001\frac{c_{15}}{c_{16}}+37.48\frac{y_{2}}{c_{12}}-0.00000058y_{17}$$

(233) $$G17\;Function:F\left(x\right)=f_{1}\left(x_{1}\right){+f}_{2}\left(x_{2}\right)$$

(234) $$G18\;Function:F\left(x\right)=-0.5(x_{1}x_{4}-x_{2}x_{3}+x_{3}x_{9}-x_{5}x_{9}+x_{5}x_{8}-x_{6}x_{7}$$

(235) $$G19\;Function:F\left(x\right)=\sum_{j=1}^{5}\sum_{i=1}^{5}c_{ij}x_{\left(10+i\right)}x_{\left(10+j\right)}+2\sum_{j=1}^{5}d_{j}x_{\left(10+j\right)}^{3}-\sum_{i=1}^{10}b_{i}x_{i}$$

(236) $$G20\;Function:F\left(x\right)=\sum_{i=1}^{24}a_{i}x_{i}$$

(237) $$G21\;Function:F\left(x\right)=x_{1}$$

(238) $$G22\;Function:F\left(x\right)=x_{1}$$

(239) $$G23\;Function:F\left(x\right)=-9x_{5}-15x_{8}+6x_{1}+16x_{2}+10\left(x_{6}+x_{7}\right)$$

(240) $$G24\;Function:F\left(x\right)=-x_{1}-x_{2}$$

Pomarine jaeger Optimization (PJO) algorithm, Tiger hunting Optimization (THO) Algorithm, Desert Reynard and Vixen Inspired Optimization (DRVIO) Algorithm, Lonchodidae optimization (LO) algorithm, Caracal optimization (CO) algorithm, Barasingha optimization (BO) algorithm, Amur leopard optimization (AO) algorithm and Empress SARANI Optimization Algorithmperformed well in evaluating the G01 – G024 Functions and results are shown below in Table 2.

**Table 2:**
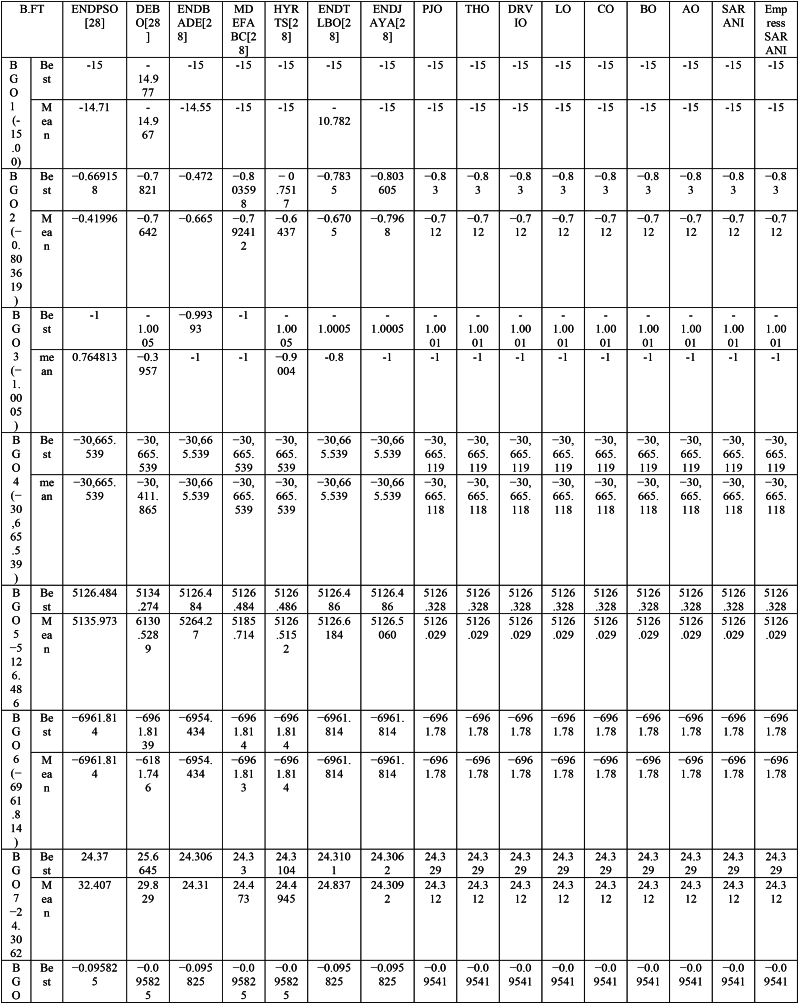

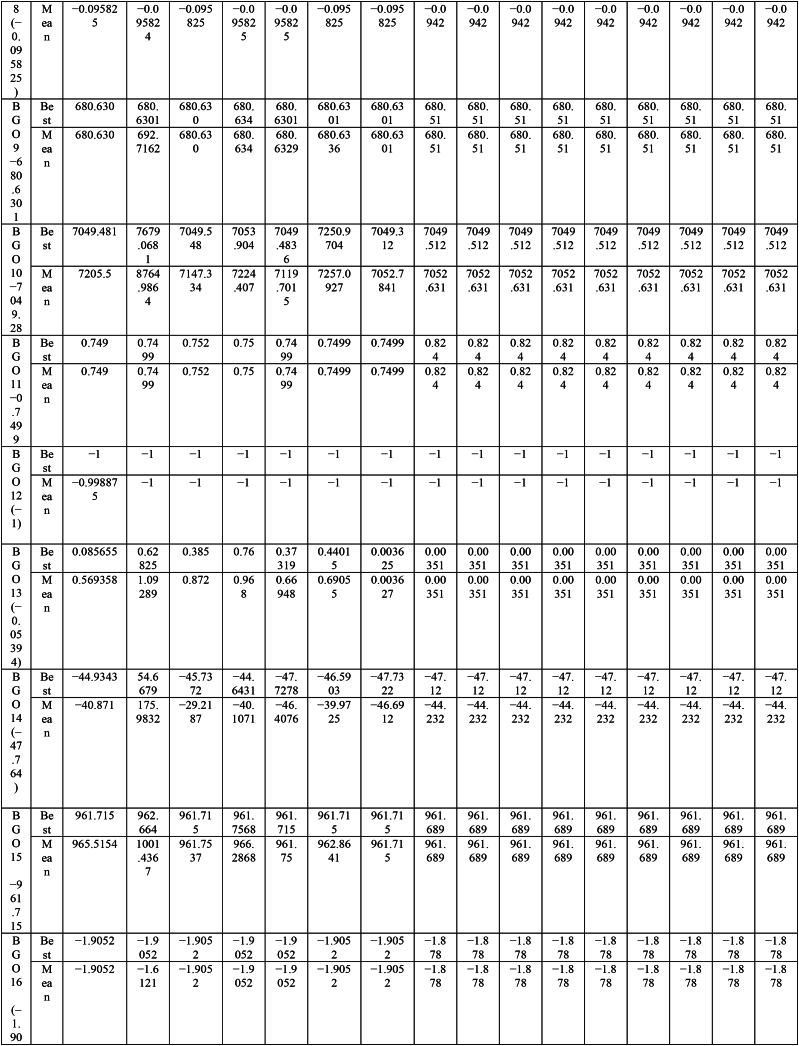

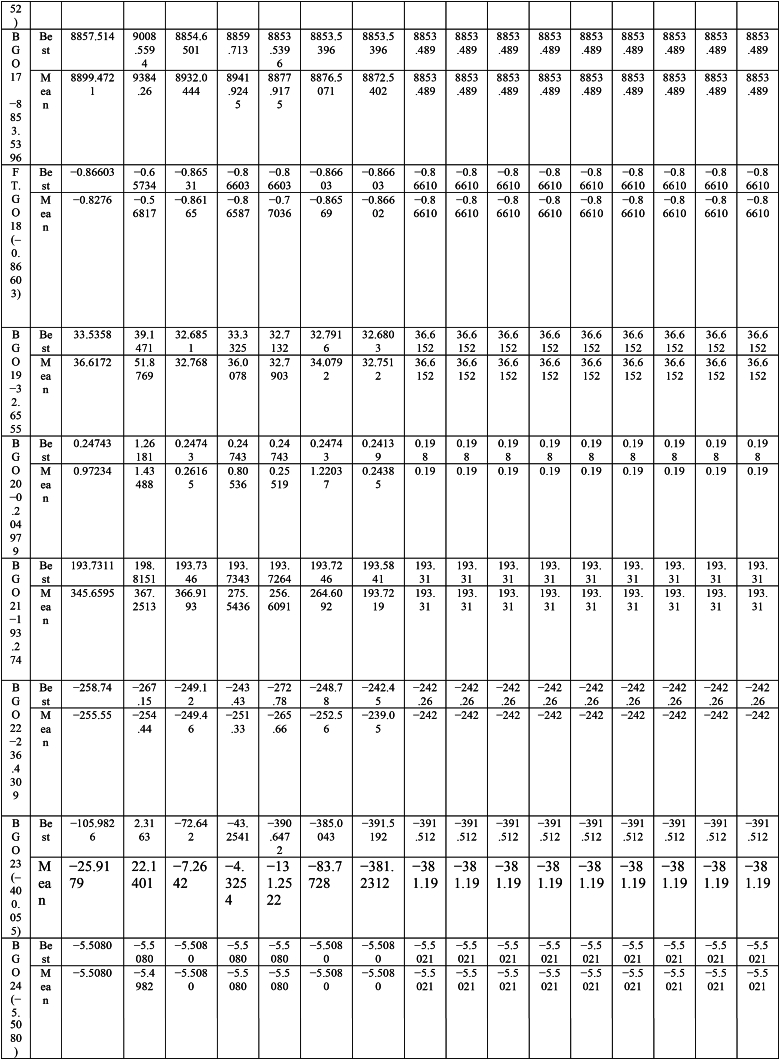
Comparative result of benchmark functions.

**Fig. 13:**
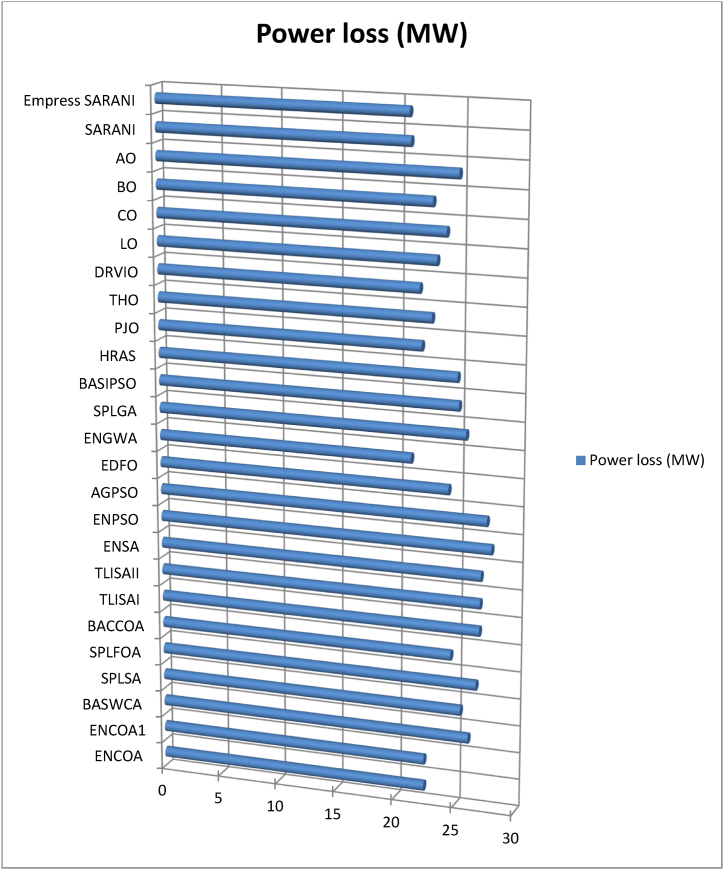
Appraisal of loss - IEEE 57 bus system (Projected algorithms-PJO, THO, DRVIO, LO, CO, BO, AO and Empress SARANI).

**Fig. 14:**
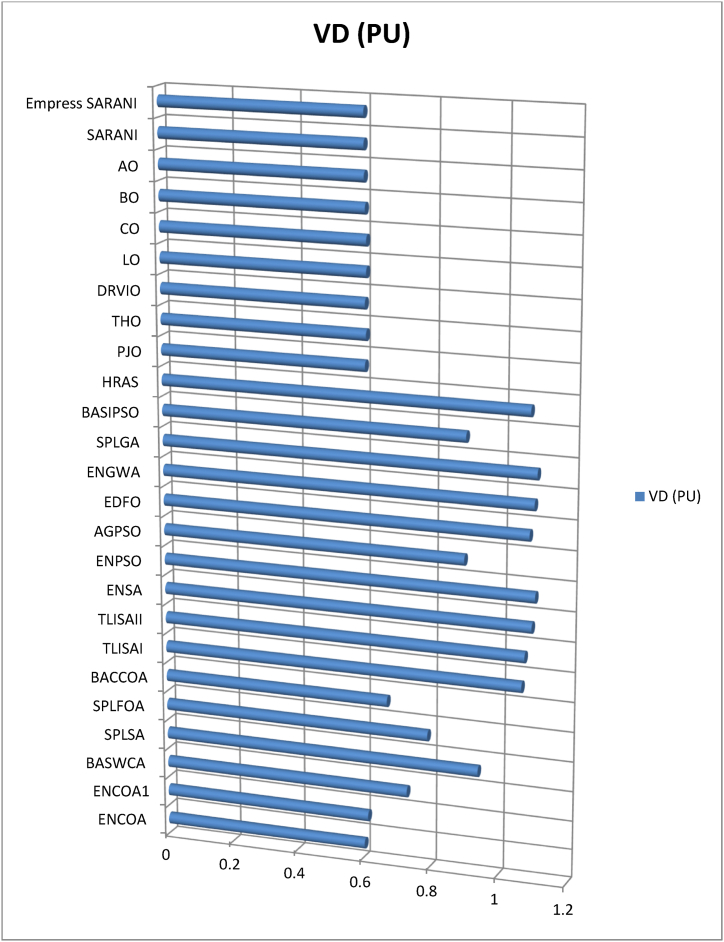
Appraisal of power deviance - IEEE 57 bus system (Projected algorithms-PJO, THO, DRVIO, LO, CO, BO, AO and Empress SARANI).

**Fig. 15:**
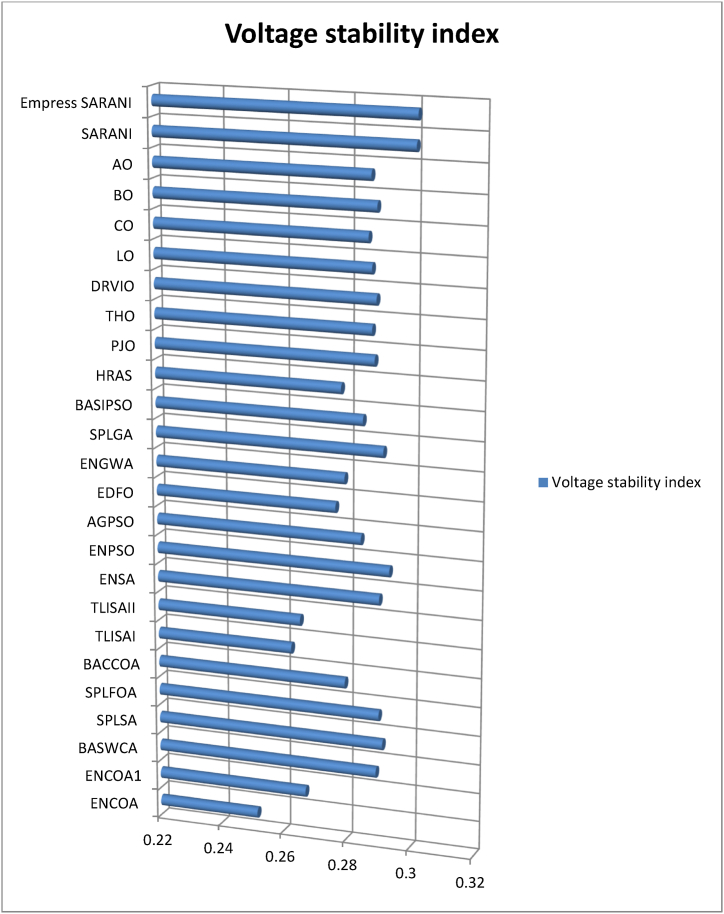
Appraisal of power permanence - IEEE 57 bus system (Projected algorithms-PJO, THO, DRVIO,LO, CO, BO, AO and Empress SARANI).

Pomarine jaeger Optimization (PJO) algorithm, Tiger hunting Optimization (THO) Algorithm, Desert Reynard and Vixen Inspired Optimization (DRVIO) Algorithm, Lonchodidae optimization (LO) algorithm, Caracal optimization (CO) algorithm, Barasingha optimization (BO) algorithm, Amur leopard optimization (AO) algorithm and Empress SARANI Optimization Algorithm are corroborated in IEEE 57 bus system (IEEE 57-bus test case system has 57 buses, 7 generators, and 42 loads) [20]. Table 3 shows the comparison of values. Fig. 13, Fig. 14, Fig. 15 give the assessment.

**Table 3:** Appraisal of power loss (IEEE 57 bus system).

Method	Power loss (MW)	VD (PU)	V.Stability (PU)
ENCOA [53]	22.376	0.6051	0.2516
ENCOA1 [53]	22.383	0.6155	0.2671
BASWCA [53]	26.0402	0.7309	0.2893
SPLSA [53]	25.3854	0.94	0.2913
SPLFOA [53]	26.6541	0.7913	0.2901
BACCOA [53]	24.5358	0.6711	0.2795
TLISAI [54]	26.88	1.0642	0.2626
TLISAII [54]	26.92	1.072	0.2654
ENSA [55]	26.97	1.0912	0.2901
ENPSO [55]	27.83	1.10	0.2932
AGPSO [55]	27.42	0.896	0.2844
EDFO [56]	24.25	1.082	0.2765
ENGWA [57]	21.171	1.095	0.2793
SPLGA [58]	25.64	1.102	0.2912
BASIPSO [58]	25.03	0.899	0.2849
HRAS [59]	24.90	1.082	0.2782
PJO	21.99	0.6062	0.2884
THO	22.79	0.6093	0.2876
DRVIO	21.79	0.6061	0.2889
LO	23.16	0.6098	0.2875
CO	23.92	0.6099	0.2864
BO	22.81	0.6061	0.2890
AO	24.89	0.6106	0.2872
SARANI	21.02	0.6023	0.3006
Empress SARANI	20.91	0.6018	0.3010

**Fig. 16:**
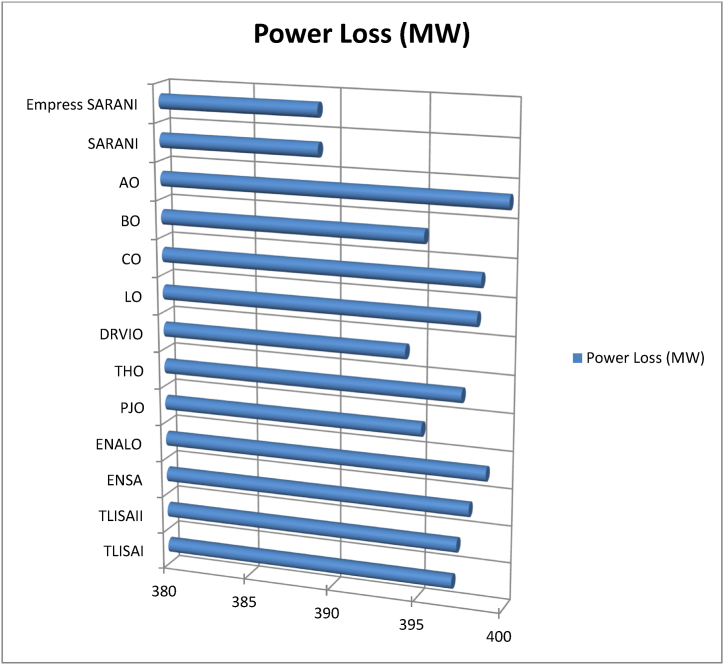
Appraisal of power loss- IEEE 300 bus system (Projected algorithms-PJO, THO, DRVIO, LO, CO, BO, AO and Empress SARANI).

**Fig. 17:**
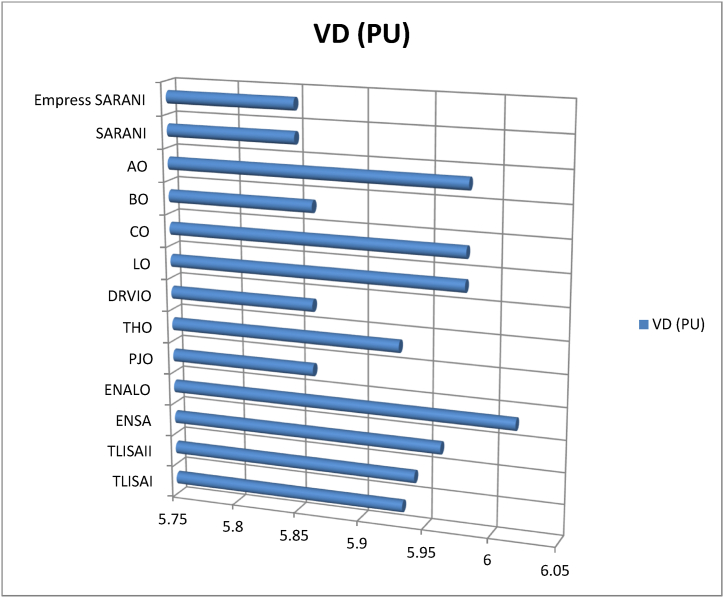
Appraisal of power deviance IEEE 300 bus system (Projected algorithms-PJO, THO, DRVIO, LO, CO,BO, AO and Empress SARANI).

Pomarine jaeger Optimization (PJO) algorithm, Tiger hunting Optimization (THO) Algorithm, Desert Reynard and Vixen Inspired Optimization (DRVIO) Algorithm, Lonchodidae optimization (LO) algorithm, Caracal optimization (CO) algorithm, Barasingha optimization (BO) algorithm, Amur leopard optimization (AO) algorithm and Empress SARANI Optimization Algorithm are corroborated in IEEE 300 bus system (IEEE 300-bus system contains 69 generators, 60 LTCs, 304 transmission lines, and 195 loads) [21]. Table 4 shows the comparison and Fig. 16, Fig. 17 give the appraisal between the approaches.

**Table 4:** Loss appraisal (IEEE 300 bus system).

Method	Power Loss (MW)	VD (PU)
TLISAI [10]	396.983	5.9324
TLISAII [10]	397.236	5.9416
ENSA [11]	397.902	5.9613
ENALO [12]	398.853	6.0169
PJO	395.153	5.8629
THO	397.398	5.9293
DRVIO	394.208	5.8629
LO	398.192	5.9782
CO	398.397	5.9788
BO	395.209	5.8631
AO	399.884	5.9801
SARANI	389.232	5.8501
Empress SARANI	389.217	5.8498

**Fig. 18:**
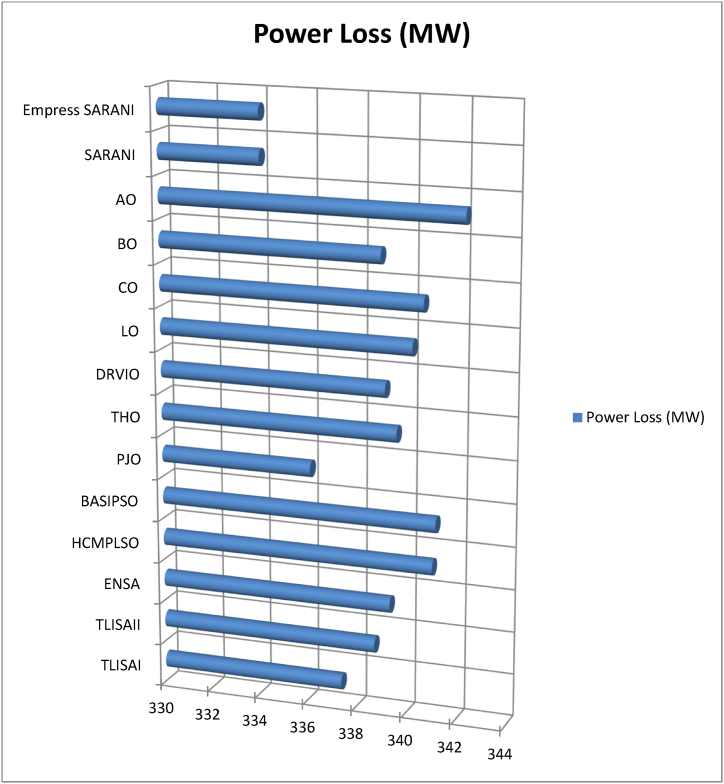
Appraisal of power loss IEEE 354 bus system (Projected algorithms-PJO, THO, DRVIO,LO, CO, BO, AO and Empress SARANI).

**Fig. 19:**
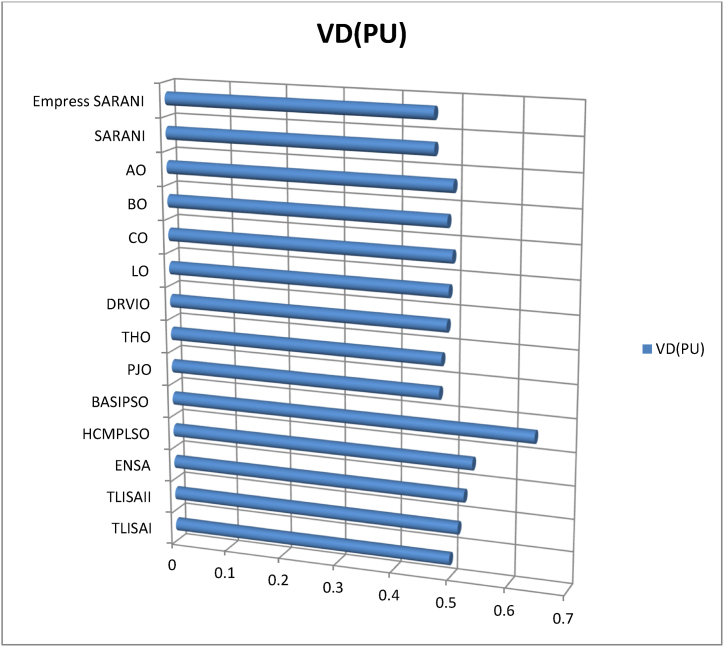
Appraisal of power deviance IEEE 354 bus system (Projected algorithms-PJO, THO, DRVIO,LO, CO, BO, AO and Empress SARANI).

Pomarine jaeger Optimization (PJO) algorithm, Tiger hunting Optimization (THO) Algorithm, Desert Reynard and Vixen Inspired Optimization (DRVIO) Algorithm, Lonchodidae optimization (LO) algorithm, Caracal optimization (CO) algorithm, Barasingha optimization (BO) algorithm, Amur leopard optimization (AO) algorithm and Empress SARANI Optimization Algorithm are corroborated in IEEE 354 bus system [21]. Table 5 shows the comparison and Fig. 18, Fig. 19 give the assessment amongst the approaches.

**Table 5:** Loss appraisal.

Method	Power Loss (MW)	VD (PU)
TLISAI [10]	337.374	0.4978
TLISAII [10]	338.715	0.5117
ENSA [11]	339.325	0.5216
HCMPLSO [12]	341.001	0.5354
BASIPSO [13]	341.123	0.6395
PJO	336.108	0.4776
THO	339.563	0.4811
DRVIO	339.099	0.4896
LO	340.164	0.4923
CO	340.592	0.4978
BO	338.906	0.4891
AO	342.184	0.4985
SARANI	334.112	0.4664
Empress SARANI	334.098	0.4653

**Fig. 20:**
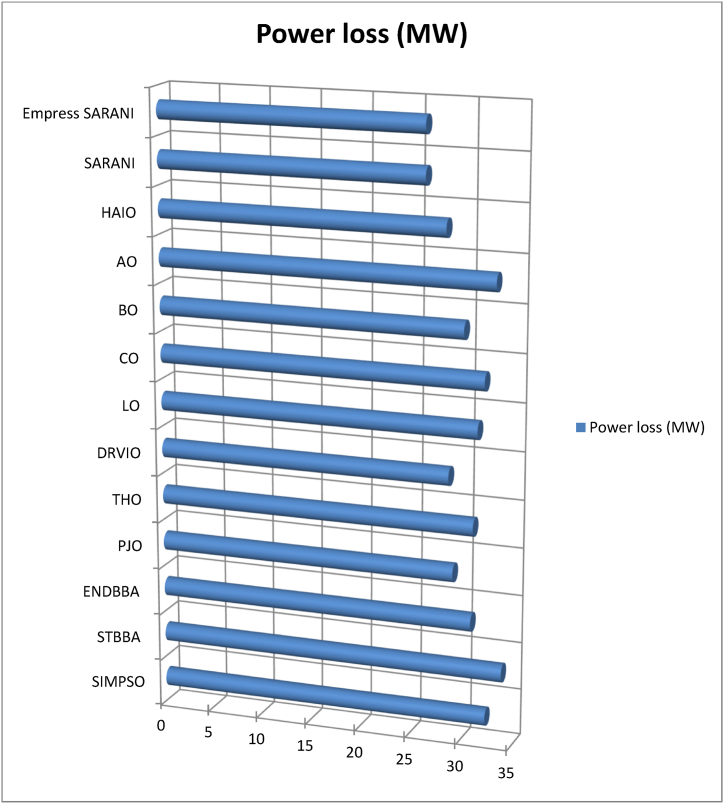
Assessment of loss- WDN 220 KV (Projected algorithms-PJO, THO, DRVIO, LO, CO, BO, AO and Empress SARANI).

**Fig. 21:**
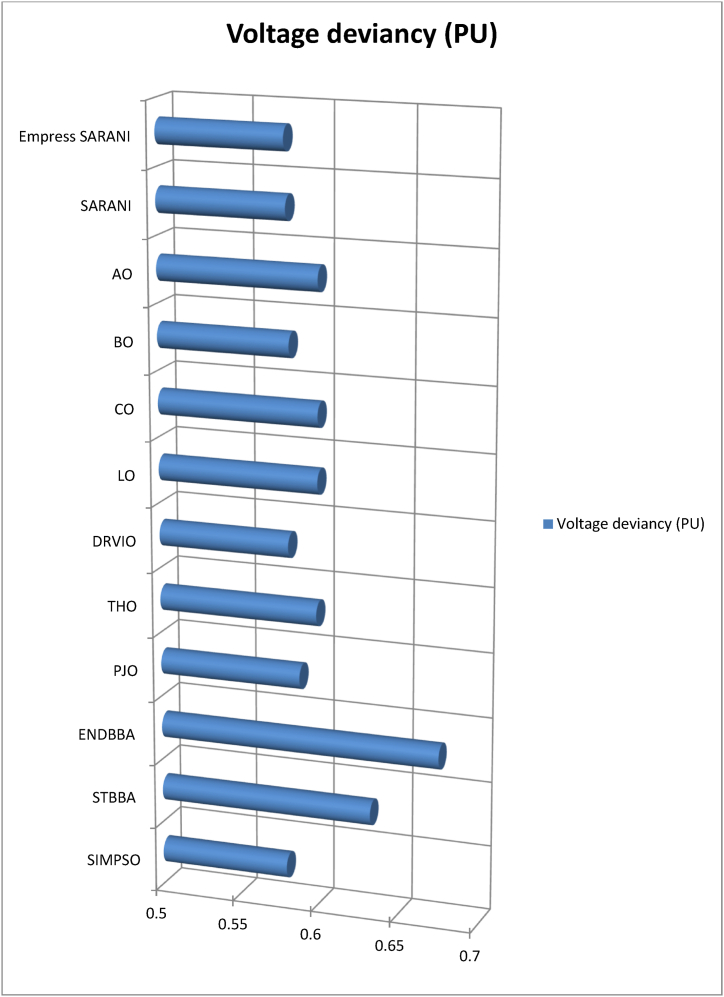
Appraisal of Voltage aberration-- WDN 220 KV (Projected algorithms-PJO, THO, DRVIO, LO, CO, BO, AO and Empress SARANI).

Pomarine jaeger Optimization (PJO) algorithm, Tiger hunting Optimization (THO) Algorithm, Desert Reynard and Vixen Inspired Optimization (DRVIO) Algorithm, Lonchodidae optimization (LO) algorithm, Caracal optimization (CO) algorithm, Barasingha optimization (BO) algorithm, Amur leopard optimization (AO) algorithm and Empress SARANI Optimization Algorithm are reviewed in practical system - WDN 220 KV [60]. Table 6 shows the comparison and Fig. 20, Fig. 21 give the assessment amongst the approaches.

**Table 6:** Loss appraisal.

Method	Power loss (MW)	VD (PU)
SIMPSO [61]	32.314	0.5800
STBBA [61]	33.875	0.6327
ENDBBA [61]	30.786	0.6751
PJO	29. 008	0.5883
THO	30. 929	0.5989
DRVIO	28. 519	0.5814
LO	31. 265	0.5996
CO	31. 893	0.5998
BO	29. 872	0.5820
AO	32. 899	0.5999
SARANI	26. 123	0.5801
Empress SARANI	26. 101	0.5790

**Fig. 22:**
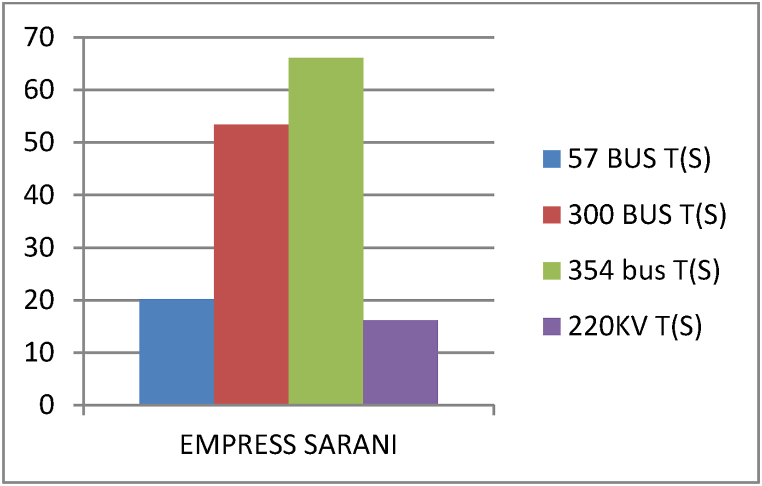
Time taken by Empress SARANI Optimization Algorithm.

Time taken by Empress SARANI Optimization Algorithm is shown in Table 7 and Fig. 22.

**Table 7:** Time taken by Empress SARANI Optimization Algorithm.

Method	57 BUS T(S)	300 BUS T(S)	354 bus T(S)	220 kV T(S)
EMPRESS SARANI	20.21	53.39	66.13	16.09

## 12 Conclusion

Empress SARANI Optimization Algorithm reduced the Active Power Loss competently. Chaotic sequences are integrated in the PJO algorithm and it will augment the Exploration and Exploitation process. Quantum features are reliable pounded of median and it has been integrated with Pomarine jaeger Optimization algorithm. Opposition based integrated in the Pomarine jaeger Optimization algorithm and it applies the Laplace distribution to augment the exploration dexterity. In THO algorithm, populace is organized and engaged in the exploration region by means of a sequence of guidelines and tactics enthused by the projected algorithm. In DRVIO algorithm, Desert Reynard and Vixen burrowing capability and spurt tactic from desolate slayers are imitated to formulate the algorithm. In LO algorithm, convergent progressions for populaces, analogous existing atmospheres are additional probable to yield analogous developments. In CO approach, initially the locations of Caracals are rationalized grounded on the imitation of these twofold schemes. This modernization grounds large alterations in the location of Caracals and tips to a comprehensive look over in examination region. In BO algorithm, the finest attained solution, hitherto, is considered as the optima around in iterations. Naturally resilient Barasingha in environment is extra brilliant in noticing, expressing to other Barasinghas of the delicacies and flee away from slayers. In AO procedure, fresh location of the Amur leopard subsequentto the bout on the prey is replicated. An active modernization is used in which the fresh location is adequate to the procedure associate if the rate of the objective function in the fresh location is extra suitable than the preceding location. SARANI algorithm uniting functions of sine-cosine, antagonizing feint of Red Dhole Optimizer Algorithm with North Sumatra Island Pongo abelii optimization algorithm will act as initial step of the method. Empress SARANI Optimization Algorithm performed well in balancing the Exploration and Exploitation. Empress SARANI Optimization Algorithm is validated in 24 benchmark functions, IEEE and Practical systems. Active Power Loss Power loss reduction, voltage stability enhancement has been accomplished along with minimization of voltage deviation.

## Future scope of work

In future Empress SARANI Optimization Algorithm can be applied to other areas of engineering and projected algorithms can be extended to apply to real times systems. Mainly projected SARANI algorithm can be utilized in the Bio-medical engineering and diagnosis system to identify certain disease and tissue growth.

## Limitations of the proposed algorithm

Proposed algorithm applied for active power loss reduction in electrical power transmission system. The scope of the algorithm can be further extended, such that it can be applied to other engineering problems.

## Declaration and acknowledgment

I.No human and animal are involved in the research
II.No funding obtained
III.No conflict of interest for the author
IV.No competing interest

## CRediT authorship contribution statement

**Lenin Kanagasabai:** Writing – review & editing, Writing – original draft, Visualization, Validation, Supervision, Software, Resources, Project administration, Methodology, Investigation, Formal analysis, Data curation, Conceptualization.

## Consent

•Single author

## Data availability statement

•No data available

## Declaration of competing interest

The authors declare that they have no known competing financial interests or personal relationships that could have appeared to influence the work reported in this paper.

## Nomenclature

**ORPD**: optimal reactive power dispatch
**RPD**: reactive power dispatch
**ΔP**: Incremental change in bus real power
**ΔQ**: Incremental change in bus reactive Power injection
**Δθ**: incremental change in bus voltage angle
**ΔV**: Incremental change in bus voltage Magnitude
$J_{R}$: reduced Jacobian matrix of the system
**ξ**: Right eigenvector matrix of JR
**η**: Left eigenvector matrix of JR
**∧**: diagonal eigenvalue matrix of JR
**n**: Number of transmission lines
**g**_**k**_: conductance of branch k
**V**_**i**_ & **V**_**j**_: are the voltage magnitude at bus i and bus j
**θ**_**ij**_: voltage angle difference between bus i and bus j
**nl**: number of load busses
**V**_**k**_: voltage magnitude at bus k
**N**_**PQ**_: set of number of PQ buses
**N**_**B**_: set of number of total buses
**P**_**G**_ & **Q**_**G**_: are the real and reactive power of the generator
**P**_**D**_ & **Q**_**D**_: are the real and reactive load of the generator
**Pgmin** & **Pgmax**: Lower &upper limits of real power generator
**Qgmin** &**Qgmax**: Lower &upper limits of reactive power generator
**Bus n°**: Bus number
**G**_**ij**_ & **B**_**ij**_: are the mutual conductance and susceptance between bus i and bus j
**Ng, Nt,Nc**: are the number of generators, number of tap transformers and the number of shunt compensators
**g**_**k**_: the conductance of branch between nodes i and j
**Nbr**: the total number of transmission lines in power systems
**P**_**d**_: the total active power demand,
**P**_**gi**_: the generator active power of unit i
**P**_**gsalck**_: the generator active power of slack bus
**ωv**: weighting factor of voltage deviation
**P**_**Di**_: Active power demand at bus i
**Q**_**Di**_: Reactive power demand at bus i
**Q**_**Gi**_: Reactive power generation at bus i
**Q**_**c**_: Reactive power generated by capacitor bank
**V**_**G**_: Generator bus voltage
**V**_**PQ**_: Voltage magnitude of PQ buses
**V**_**PV**_: Voltage magnitude of PV buses
**V**_**S**_: Voltage magnitude of slack bus
**P**_**loss**_: Real power loss of the system
**Q**_**loss**_: Reactive power loss
**Tk**: Tap position of transformer k
**kn**: acceleration coefficient
**ky**: velocity coefficient
**SVSM**: static voltage stability margin
**VSCRPD**: Voltage stability constrained reactive power dispatch
